# Conserved rules govern genetic interaction degree across species

**DOI:** 10.1186/gb-2012-13-7-r57

**Published:** 2012-07-02

**Authors:** Elizabeth N Koch, Michael Costanzo, Jeremy Bellay, Raamesh Deshpande, Kate Chatfield-Reed, Gordon Chua, Gennaro D'Urso, Brenda J Andrews, Charles Boone, Chad L Myers

**Affiliations:** 1 Department of Computer Science and Engineering, University of Minnesota, 200 Union Street SE, Minneapolis, MN 55455, USA; 2 Banting and Best Department of Medical Research, Terrence Donnelly Centre for Cellular and Biomolecular Research, University of Toronto, 160 College Street, Toronto, Ontario M5S 3E1, Canada; 3 Department of Molecular Genetics, Terrence Donnelly Centre for Cellular and Biomolecular Research, University of Toronto, 160 College Street, Toronto, Ontario M5S 3E1, Canada; 4 Institute for Advanced Computer Studies, University of Maryland College Park, 3115 Biolmolecular Sciences Bldg #296, College Park, MD 20742, USA; 5 Institute of Biocomplexity and Informatics, Department of Biological Sciences, University of Calgary, 2500 University Drive NW, Calgary, AB T2N 1N4 Canada; 6 Department of Molecular and Cellular Pharmacology, University of Miami School of Medicine, PO Box 016189, Miami, FL 33101, USA

## Abstract

**Background:**

Synthetic genetic interactions have recently been mapped on a genome scale in the budding yeast *Saccharomyces cerevisiae*, providing a functional view of the central processes of eukaryotic life. Currently, comprehensive genetic interaction networks have not been determined for other species, and we therefore sought to model conserved aspects of genetic interaction networks in order to enable the transfer of knowledge between species.

**Results:**

Using a combination of physiological and evolutionary properties of genes, we built models that successfully predicted the genetic interaction degree of *S. cerevisiae *genes. Importantly, a model trained on *S. cerevisiae *gene features and degree also accurately predicted interaction degree in the fission yeast *Schizosaccharomyces pombe*, suggesting that many of the predictive relationships discovered in *S. cerevisiae *also hold in this evolutionarily distant yeast. In both species, high single mutant fitness defect, protein disorder, pleiotropy, protein-protein interaction network degree, and low expression variation were significantly predictive of genetic interaction degree. A comparison of the predicted genetic interaction degrees of *S. pombe *genes to the degrees of *S. cerevisiae *orthologs revealed functional rewiring of specific biological processes that distinguish these two species. Finally, predicted differences in genetic interaction degree were independently supported by differences in co-expression relationships of the two species.

**Conclusions:**

Our findings show that there are common relationships between gene properties and genetic interaction network topology in two evolutionarily distant species. This conservation allows use of the extensively mapped *S. cerevisiae *genetic interaction network as an orthology-independent reference to guide the study of more complex species.

## Background

Most genes are not essential for eukaryotic life under standard laboratory conditions, which may reflect that organisms are highly buffered from genetic and environmental perturbations [1]. However, rare combinations of singly benign genetic variation can lead to synergistic effects, such as synthetic lethality, where mutations in two genes, neither of which is lethal independently, combine to generate an inviable double-mutant phenotype [2]. Because natural variations that distinguish two people occur relatively frequently [3] and complex genetic interactions may underlie most individual phenotypes [1], understanding the general principles that govern genetic networks may be critical for solving the genotype-to-phenotype problem and implementing personal medicine [4].

Recently, we tested approximately 5.4 million *Saccharomyces cerevisiae *gene pairs for genetic interactions, mapping an extensive network of more than 100,000 interactions by synthetic genetic array (SGA) analysis [5]. The study discovered both negative genetic interactions, instances in which a double mutant exhibits a more extreme phenotype than the expected combined effect of the single mutants, as well as positive genetic interactions, instances in which a double mutant exhibits a less-pronounced phenotype than expected [6]. This study revealed the distribution of genetic interactions with respect to gene function, highlighting a central role for chromatin-related, transcription, and secretory functions. Additionally, it identified several fundamental physiological and evolutionary gene properties that are significantly correlated with genetic interaction degree in the *S. cerevisiae *genetic interaction network [5]. For example, we showed that the genetic interaction degree of a gene is highly correlated with single mutant fitness, such that genes with a substantial fitness defect show a large number of genetic interactions.

While genetic interactions have been the most extensively studied in the yeast *S. cerevisiae*, there is intense interest in developing and applying large-scale screening technologies in other species. For example, large studies have already been completed in *Escherichia coli *[7,8], *Schizosaccharomyces pombe *[9,10], *Caenorhabditis elegans *[11,12], *Drosophila melanogaster *[13,14], and human cell lines [15-17]. Although definitive comparative analysis of these networks across species would be premature given the sparsity of known interactions in species other than *S. cerevisiae*, there have been preliminary comparative studies. In particular, the yeast *S. pombe *provides an attractive setting for this analysis due to the availability of a genome-wide deletion mutant collection [18] and scalable technology for automated genetic analysis [2]. Furthermore, *S. cerevisiae *and *S. pombe *are estimated to have diverged approximately 500 million years ago and display markedly different physiological properties [19] but share 75% of their gene content [19,20]. The two comparative studies to date estimated approximately 30% conservation of individual negative genetic interactions, but also found substantial differences between the two species [21,22]. These studies demonstrate the power and necessity of comparative analysis of genetic interaction networks, but have conducted only limited sampling of genetic interactions in *S. pombe*. The properties of these networks that are conserved across species and the rules governing their evolution remain largely open questions, making further characterization of the evolution of genetic interaction networks important.

In this study, we build predictive models capturing the relationship between gene properties and genetic interaction network connectivity, demonstrating that a small set of properties can accurately predict degree in the *S. cerevisiae *genetic interaction network. We show that models built from the genome-scale *S. cerevisiae *genetic interaction network also successfully predict genetic interaction degree of *S. pombe *genes, the vast majority of which have not previously been screened for interactions. Finally, we use our model to predict differences between the *S*. *pombe *and the *S. cerevisiae *networks, some of which may be reflective of functional rewiring and physiological differences between the species, and show that these differences are independently supported by the divergence of co-expression networks based on comparative analysis of gene expression.

## Results and discussion

### Modeling interaction degree in the *S. cerevisiae *genetic interaction network

**Figure 1:**
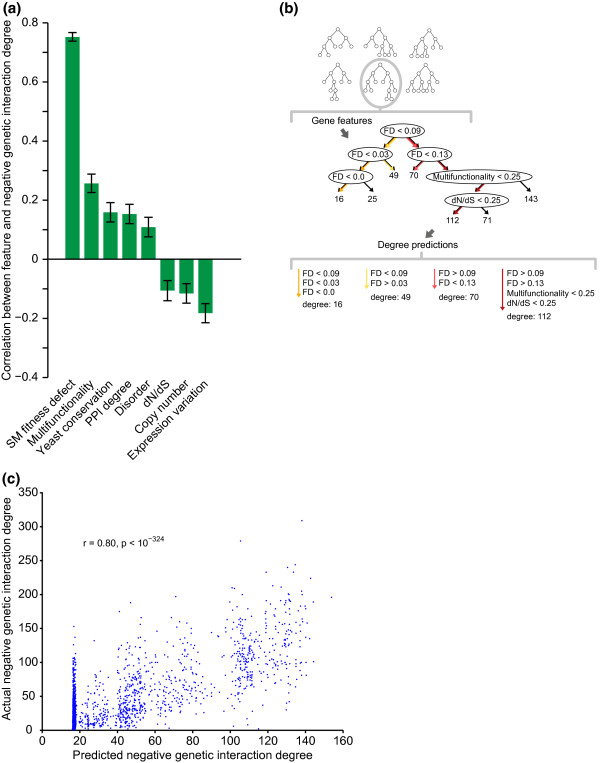
**Physiological and evolutionary gene features are predictive of genetic interaction degree**. **(a) **Gene features are significantly correlated with negative genetic interaction degree. We measured the Pearson correlation coefficients between gene feature values and negative genetic interaction degree for 3,456 non-essential *S. cerevisiae *genes. Error bars show 95% confidence intervals. A complete set of features and their correlations is given in Table 1; see Materials and methods for descriptions of gene features. **(b) **Overview of the regression tree model for genetic interaction degree. An ensemble of 100 decision trees was built from bootstrap samples of genes. Combinations of values of features are represented as paths from the root to the leaves of a tree. Internal nodes each split data (sets of genes) according to values for a single feature; leaf nodes are associated with predicted genetic interaction degrees. **(c) **Scatter plot of negative genetic interaction degree and degrees predicted by the bagged decision tree model on held-out genes shows the significant relationship between predicted and actual degrees (Pearson's r = 0.80, *P *< 10^-324^). FD, fitness defect; PPI, protein-protein interaction; SM, single mutant.

Highly connected genes in the *S. cerevisiae *genetic interaction network are often associated with slow-growing single mutants, protein products with disordered structure, gene pleiotropy as indicated by multiple Gene Ontology (GO) annotations, high connectivity in the physical interaction network, slower rates of evolution, and low expression variation (Figure 1a; Materials and methods) [5], as well as a number of other sequence- and experimental-based gene features (Table 1). We reasoned that these correlations could serve as the basis for predictive modeling of interaction degree, enabling the prediction of interaction degrees for genes that have not yet been screened.

**Table 1:** Pearson correlations between features and negative genetic interaction degree in *S. pombe *(*pom*) and *S. cerevisiae *(*cer*) are observed to be significant in many cases

	Pearson's r	*P*-value	95% CI
SM fitness defect			
*pom*	0.48	9.90E-31	[0.41, 0.54]
*cer*	0.75	0.00E+00	[0.77, 0.74]
Multifunctionality			
*pom*	0.3	1.01E-12	[0.22, 0.37]
*cer*	0.26	4.52E-53	[0.29, 0.23]
Conservation			
*pom*	0.07	1.06E-01	[-0.01, 0.15]
*cer*	0.16	7.11E-21	[0.19, 0.13]
Broad conservation			
*pom*	0	9.30E-01	[-0.09, 0.08]
*cer*	0.16	9.90E-19	[0.19, 0.12]
PPI degree			
*pom*	0.2	5.84E-03	[0.06, 0.33]
*cer*	0.15	1.49E-19	[0.19, 0.12]
Expression level			
*pom*	-0.05	2.42E-01	[-0.13, 0.03]
*cer*	0.11	7.40E-10	[0.14, 0.08]
Disorder			
*pom*	0.13	3.05E-03	[0.04, 0.21]
*cer*	0.11	1.91E-10	[0.14, 0.08]
Codon Adaptation Index			
*pom*	-0.02	6.49E-01	[-0.1, 0.06]
*cer*	0.09	1.91E-07	[0.12, 0.06]
Protein length			
*pom*	0	9.18E-01	[-0.08, 0.09]
*cer*	0.05	3.57E-03	[0.08, 0.02]
Co-expression degree			
*pom*	0	9.38E-01	[-0.08, 0.09]
*cer*	0.05	3.75E-03	[0.08, 0.02]
Number of domains			
*pom*	-0.01	7.37E-01	[-0.1, 0.07]
*cer*	0.01	4.54E-01	[0.05, -0.02]
Number of unique domains			
*pom*	-0.02	6.86E-01	[-0.1, 0.07]
*cer*	0.01	6.98E-01	[0.04, -0.03]
Nc			
*pom*	-0.01	7.56E-01	[-0.1, 0.07]
*cer*	-0.08	2.95E-06	[-0.05, -0.11]
dN/dS			
*pom*	-0.05	2.48E-01	[-0.14, 0.04]
*cer*	-0.11	1.58E-09	[-0.07, -0.14]
Copy number			
*pom*	-0.08	5.26E-02	[-0.17, 0]
*cer*	-0.12	1.01E-11	[-0.08, -0.15]
Expression variation			
*pom*	-0.15	4.11E-04	[-0.23, -0.07]
*cer*	-0.18	3.87E-27	[-0.15, -0.21]

CI, confidence interval; PPI, protein-protein interaction; SM, single mutant

To this end, we applied a regression tree approach to model combinations of 16 gene features that are predictive of negative genetic interaction degree (Figure 1b). Regression trees are built by repeatedly splitting sets of training genes, according to the values of gene features, until genes are sorted into small sets that each contain genes with similar genetic interaction degrees. The hierarchy of gene sets produced is visualized as a binary tree and the final sets of genes are each associated with linear regression models that assign predictions to query genes (Figure 1b). Bootstrapped subsets of the training data were used to build an ensemble of regression trees; this use of multiple models, bootstrap aggregation, is a typical method for building a robust predictive model [23] (Materials and methods).

To validate our approach, we used our model to predict negative genetic interaction degree for all genes in the *S. cerevisiae *genetic interaction network (Figure 1c; Materials and methods). A high correlation (r = 0.80, *P *< 10^-324^) was observed between predicted and actual genetic interaction degrees of genes not used in training the models, indicating that our model accurately reflects topological features of the *S. cerevisiae *genetic interaction network (Figure 1c). A strength of this type of model, in addition to providing degree predictions for previously unseen genes, is that the learned tree structures highlight rules consisting of combinations of gene features that explain variation in degree (Figure 1b).

### Predicting genetic interaction degree in a distantly related species

**Figure 2:**
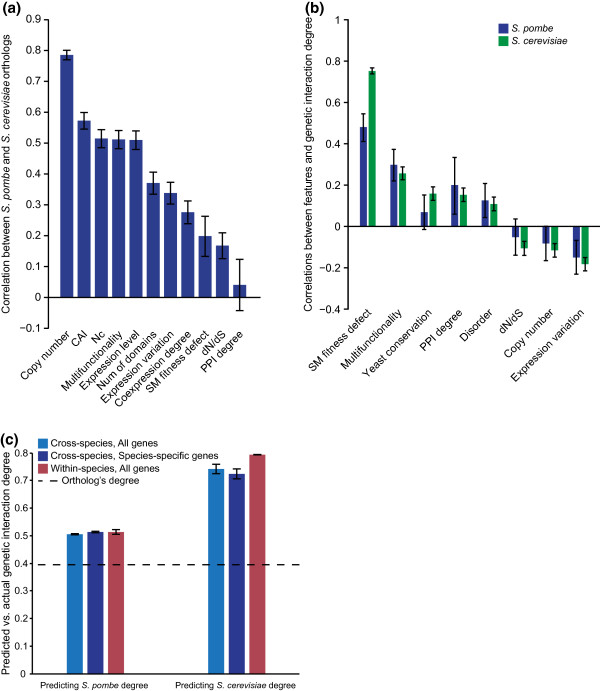
**Cross-species analysis of the predictive model for genetic interactions**. **(a) **Pearson correlations between one-to-one *S. cerevisiae *and *S. pombe *orthologs for their values of gene features. Note that a number of features are sequence-based and are thus not independent of the sequence-based ortholog identification; features that appear to have trivial correlations are not included here. Error bars show 95% confidence intervals. **(b) **Pearson correlations between features and degree in *S. pombe *are observed to be significant in many cases and similar to those in *S. cerevisiae*. A complete set of features and their correlations is given in Table 1; see Materials and methods for descriptions of gene features. Error bars show 95% confidence intervals. **(c) **Predictive abilities of bagged regression tree models were evaluated by measuring Pearson correlations between predicted and actual degrees. The left set of bars shows the performance of predictions made for approximately 550 *S. pombe *genes and the right set of bars shows the performance of predictions made for all non-essential deletion mutants in *S. cerevisiae*. For each scenario, models were trained both on data from the same species (red bar) as well as data from the other species (blue bars). The light blue bars correspond to predicting degrees of all genes in the test species, while the dark blue bars correspond to predicting degrees of genes in the subset of genes lacking orthologs in the training species. Error bars show standard deviations of bootstrapped predictions. For a baseline, the dashed line shows the correlation between observed degrees of one-to-one orthologous genes (a simple prediction method that can be applied to only orthologs). CAI, Codon Adaptation Index; PPI, protein-protein interaction; SM, single mutant.

If the rules governing genetic network topology are conserved, then a model based on *S. cerevisiae *gene features should be predictive of genetic interaction degree in other organisms. To test this, we examined the same gene features of *S. pombe *genes that we found to be predictive of *S. cerevisiae *interaction degree, including a quantitative measurement of single mutant fitness defects across the genome (Materials and methods). Surprisingly, comparative analysis of the various features between pairs of orthologs revealed that a number of non-sequence-based features are only modestly conserved between the two yeast species [24] (Figure 2a; Materials and methods). For example, we found a significant but relatively weak correlation in single mutant fitness (Pearson's r = 0.20, *P *< 10^-8^) between 1,100 one-to-one orthologous gene pairs for which we could derive fitness measurements in both yeasts. The lack of strong conservation of deletion mutant fitness is somewhat surprising given that approximately 80% of *S. pombe *orthologs of *S. cerevisiae *essential genes have conserved essentiality [18]. Thus, while *S. cerevisiae *and *S. pombe *share a common set of genes that are indispensable for viability, our findings suggest that the severity of fitness defects imposed by the deletion of orthologous non-essential genes for growth under standard laboratory conditions is not well conserved. Other gene properties, including protein-protein interaction degree, dN/dS, and multifunctionality, exhibit a similar lack of conservation (Figure 2a).

Despite the low conservation of single mutant fitness and the varying correlations between individual gene properties for orthologs, we found that relationships between *S. pombe *gene features and genetic interaction degree were strikingly similar to those observed in *S. cerevisiae *(Figure 2b, Table 1). Consistent with *S. cerevisiae *trends (Figure 1a, Table 1), fitness defect was the strongest predictor of *S. pombe *genetic interaction degree. That is, *S. pombe *strains with severe fitness defects often exhibit high numbers of genetic interactions. The observed trends suggested that in addition to correlation with individual gene features, higher-level combinations of features that are predictive of connectivity in the *S. cerevisiae *genetic interaction network [5] (Figure 1a) may also be informative of *S. pombe *genetic interaction degree.

To test this hypothesis, we built a predictive model relating the combination of available gene features to genetic interaction degree in *S. cerevisiae *and then applied the resulting model to predict genetic interaction degree in *S. pombe *(Materials and methods). Interestingly, we observed significant correlation (r = 0.51, *P *< 10^-36^) between interaction degree predicted by our model and the number of interactions previously determined [10] for 548 *S. pombe *genes (Figure 2c, left side, light blue bar).

Our ability to predict genetic interaction degree from a small set of gene-specific properties is evidence that rules governing genetic interaction network topology are conserved across a large evolutionary distance (Figure 2c). Importantly, there is no significant decrease in correlation between predicted and actual interaction degree when predictions were restricted to genes unique to *S. pombe *(Figure 2c, left side, dark blue bar), indicating that the model performs equally well when applied to genes lacking orthologs in the species used to learn relationships in the model.

As a baseline comparison for our cross-species predictive model, we built a model from *S. pombe *gene features and genetic interaction degrees instead of from *S. cerevisiae *data. Within-species predictions for *S. pombe *interaction degrees are not significantly more accurate than predictions made by the cross-species model (Figure 2c, left side, compare red and light blue bars). We also note that although a simplistic predictor that maps the degree of a *S. cerevisiae *gene directly to its *S. pombe *ortholog provides reasonable performance (Pearson correlation approximately 0.4), this strategy is out-performed by our cross-species model and is limited to conserved genes. Strikingly, the models trained on *S. pombe *interactions and features were also able to predict interaction degree in the *S. cerevisiae *network with high accuracy (Figure 2c, right side, compare red and light blue bars). In general, interaction degree predictions for *S. pombe *genes were weaker than *S. cerevisiae *interaction degree predictions, which may reflect the limited functional diversity of available *S. pombe *genetic interaction studies [9,10]. Nonetheless, the ability to predict interaction degree using features measured in either yeast species is evidence that relationships between genetic interactions and fundamental physiological and evolutionary properties are generally conserved.

The strong correlation between single mutant fitness defect and negative genetic interaction degree has the unsurprising consequence that the models are considerably influenced by this feature. To explore the reliance of our model on fitness defect, we constructed two types of bootstrapped regression tree models that were trained on all features except fitness defect. The first of these additional models is trained to predict negative genetic interaction degrees and is able to successfully make both within- and cross-species predictions (Figure S1 in Additional file 1). The second model was trained to predict the residual negative genetic interaction degree that remained after subtracting degree predictions made from a regression tree model that was trained on the single feature single mutant fitness defect. The prediction of these residuals by the remaining features was also significant (Figure S2 in Additional file 1). We therefore consider the inclusion of many other features to be a worthwhile part of our model, since they capture aspects of genetic interaction degree that fitness defect alone does not.

### Validating predictions with *S. pombe *whole-genome screens

**Figure 3:**
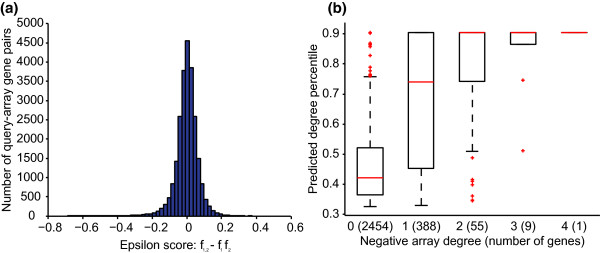
**Observed genetic interactions between *S. pombe *genes support degree predictions**. **(a) **Model predictions were validated on a second, whole-genome set of interaction screens in *S. pombe *that are independent of the training data. Eight query deletion mutants were crossed with the entire non-essential deletion collection in *S. pombe*. In total, genetic interaction (epsilon) scores were measured for approximately 23,000 gene pairs. Epsilon scores are tightly centered at 0, thus interactions called for scores of ± 0.08 or more extreme are rare. **(b) **The collection of non-essential *S. pombe *genes (*n *= 2,907) were grouped by the number of interactions each has with the eight query genes for which full-genome screens were performed. Numbers in parentheses give the number of genes for which this degree was observed. For each degree, the box plot shows the distribution of predicted degrees, which are expressed as percentiles. There is a strong positive correlation (Pearson's r = 0.40, *P *< 10^-111^) between predicted and actual degree.

As an independent validation of our model, we conducted genome-wide *S. pombe *genetic interaction screens. Eight query gene-deletion mutants spanning diverse cellular functions were crossed into an array of 2,907 non-essential *S. pombe *deletion mutants [2,18], making approximately 23,000 double mutant strains (Figure 3a; Materials and methods).

Consistent with our results for a published dataset [22] (Figure 2c), we observed a significant correlation (r = 0.40, *P *< 10^-111^) between the predicted number of interactions and the total number of experimentally derived interactions observed for a given array mutant in this genome-wide deletion set. Grouping genes with the same observed degree, we found that the distributions of our predictions were reflective of actual degrees (Figure 3b). For example, the median degree percentile predicted for genes with a degree of one was approximately 0.72, while the median prediction for genes with zero interactions was approximately 0.42. Importantly, the significance of the correlation was robust to the choice of interaction cutoff and persisted for a higher-confidence, sparser network (Materials and methods).

### Identifying network rewiring suggested by cross-species predictions

Although many individual genes are conserved, yeast genetic interaction networks may have undergone substantial rewiring, as only approximately 30% of the interactions are conserved [9]. Similarly, a low conservation of genetic interactions has also been observed between *S. cerevisiae *and *C. elegans *[25]. To examine the extent of network rewiring, we first inferred interaction degrees for the entire *S. pombe *genome using our cross-species model. Because the predictions do not depend on sequence orthologs (Figure 2a, c), they can be used to compare the topologies of the *S. cerevisiae *and *S. pombe *networks even though only a small fraction of the *S. pombe *network has been screened.

**Figure 4:**
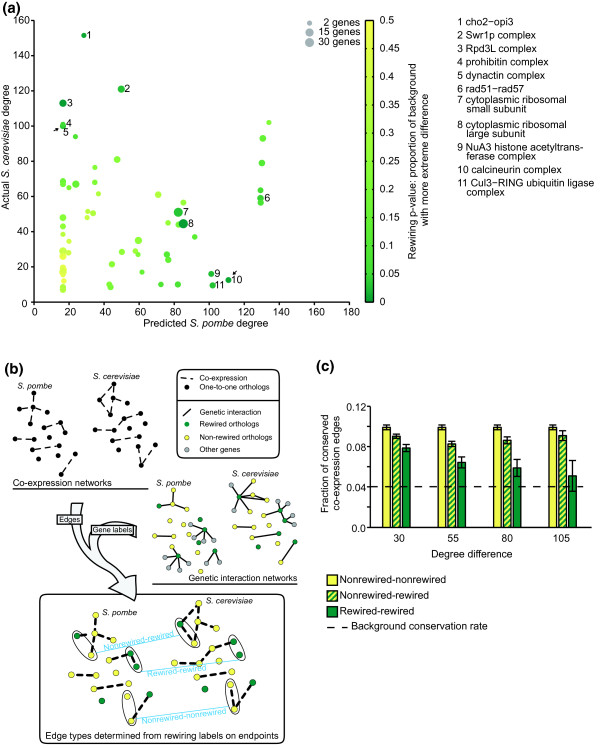
**Global analysis of rewiring based on whole-genome predictions in *S. pombe***. **(a) **Points in the scatter plot each represent groups of between 2 and 22 genes whose protein products are in the same protein complex (Materials and methods). Darker color represents complexes that are predicted to have significant rewiring. Generally, genes in complexes that fall on the diagonal are predicted to have conserved degrees, while those that fall off-diagonal show evidence for large degree differences between the two species. Significantly rewired complexes (at a threshold of 0.05) are labeled by their names. **(b) **To validate our predicted rewired genes, we constructed separate networks of co-expression relationships among genes for each yeast species, then labeled genes according to our rewiring designation. Only one-to-one orthologs that are non-essential in both species were included in the networks. Edges in the co-expression network were classified by whether involved genes were both rewired, only one was rewired, or neither was rewired. We then calculated fractions of conserved co-expression relationships between species within each of these classes. **(c) **There is a clear relationship between these classes of edges and their conservation across the two yeast species. For rewiring at four levels of magnitude, we counted the number of conserved edges (among all edges in the union of the two networks). A conserved edge appears in the networks of both species and a non-conserved edge appears in exactly one. The magnitude of rewiring increases along the x-axis for the rewired class (differences of > 30, > 55, > 80, > 105 interactions), but the non-rewired class is defined as the set of ortholog pairs with less than a 30-edge difference in degree. Edges in the two rewired classes consistently showed significantly lower levels of conservation than edges in the non-rewired class (*P *< 0.01, Fisher's exact test). Error bars show the binomial proportion 95% confidence interval. The dashed line is the expected rate of conservation if edges are randomized in one of the co-expression networks. There are 12,472 edges among 509 genes in the conserved-conserved network. Numbers of edges and genes at rewiring thresholds, in bold, are as follows, where the conserved-rewired case is given as the first pair and the rewired-rewired case is given second: **30: **(14532, 832), (4684, 323); **55: **(8730, 695), (1659, 186); **80: **(5358, 620), (644, 111); **105: **(2822, 565), (176, 56).

We found several instances where the predicted interaction degree for a given *S. pombe *gene was quite different from the observed degree of its *S. cerevisiae *ortholog, suggesting that the gene acquired or lost interactions differentially as the species diverged. To identify larger functional modules that were targets of this rewiring, we grouped functionally related genes according to a catalog of 65 annotated protein complexes [6] and 545 GO biological process annotations [26] (Materials and methods), and compared the median interaction degrees determined for orthologous protein complexes and functional groups (Figure 4a; Figure S3 in Additional file 1). Many groups of functionally related genes and several complexes were statistically indistinguishable in terms of network connectivity, indicating that these modules act either as network hubs in both species or non-hubs in both species.

However, we also identified many examples of possible rewiring, in which a significant difference in network connectivity, observed in *S. cerevisiae *and inferred in *S. pombe*, was found for orthologous modules (Figure 4a; Figure S3 in Additional file 1; Materials and methods). These predicted-rewired groups represent complexes or biological processes that may have evolutionarily diverged in terms of their importance in the genetic interaction network, acting as hubs in one species but not in the other. In particular, we found that 11 of 65 (17%) protein complexes and 44 of 545 (8%) GO biological processes may have undergone significant rewiring (Figure 4a; Figure S3 in Additional file 1) at a level of significance expected to identify only 3 and 27 (5%) rewired modules, respectively. For example, components of the dynactin complex are hub genes in the *S. cerevisiae *genetic interaction network (complex average of 85th percentile; Figure 4a) whereas the orthologous genes were predicted to exhibit average connectivity in the *S. pombe *genetic interaction network (complex average of approximately 50th percentile; Figure 4a). Dynactin, a multi-subunit protein complex known for interacting with dynein and enabling long-range movement along microtubules (reviewed in [27]), has been implicated in a *S. cerevisiae *cell cycle checkpoint pathway that arrests cell cycle progression in response to perturbations in cell wall synthesis [28]. A similar checkpoint has not been reported in *S. pombe*, suggesting that the difference in the number of genetic interactions observed across species may reflect a dynactin-specific role in monitoring *S. cerevisiae *cell wall integrity.

In addition to *S. cerevisiae*-specific genetic interaction hubs, we also identified gene groups predicted to be hubs in the *S. pombe *but not observed as such in the *S. cerevisiae *genetic network. One such case is the calcineurin-associated protein complex (Figure 4a). A difference in network connectivity might reflect a unique role for calcineurin in the regulation of bi-polar growth activation in *S. pombe *[29]. Unlike an *S. cerevisiae *cell, which grows predominantly via an actin-dependent budding mechanism, an *S. pombe *cell grows in a highly polarized bi-polar manner from its two ends. Following cell division, cell growth is initiated from the old end first, and later, after completion of S phase, from the newer end that forms at the site of cell septation (referred to as new end take off, or NETO). Calcineurin has been shown to play an important role in the delay of NETO by directly dephosphorylating critical targets involved in microtubule dynamics at the site of cell growth. This mechanism is dependent on activation of Cds1 kinase, best known for its role in the intra-S phase DNA replication checkpoint [30]. A connection between the intra-S phase checkpoint and inhibition of bipolar growth activation is so far unique to *S. pombe *and distinct from the checkpoint controls operating in *S. cerevisiae*. Additionally, calcineurin is dispensable for growth in *S. cerevisiae *[31]; in *S. pombe*, its deletion leads to defects in cell growth, cytokinesis, cell polarity, mating, and spindle pole body positioning, which are widespread effects consistent with its hub-like activity [32].

While our method of identifying rewired modules reports several statistically significant differences, we note two caveats in interpreting these results. First, since degrees of genes within functional modules may be systematically poorly predicted, our procedure may incorrectly identify modules as significantly rewired in cases where our test statistic would also have indicated that the within-species difference between predicted and observed degree was significant. Therefore, as a control, a version of this rewiring experiment that compares observed and predicted *S. cerevisiae *degrees will enable identification of cases that do not reflect true cross-species rewiring (Figure S4a, b in Additional file 1). Second, due to variations in the experimental protocol for measuring genetic interactions, there are differences in the media on which fitness defects were measured in *S. cerevisiae *and *S. pombe*, which may also contribute to apparent rewiring [33].

Functional properties of genes can be captured by many types of biological networks, so we turned to an independent dataset for confirmation of our rewiring predictions. To enable a comparative analysis of gene expression profiles across the two yeasts, we constructed a species-specific *S. pombe *co-expression network using a previously published approach [34] and large collections of publicly available expression data (Materials and methods), and obtained a previously published *S. cerevisiae *network [35]. Each species' network contains 832 genes that are one-to-one orthologs between the two yeasts and connected genes are those pairs that have high co-expression values surpassing a threshold of the 95th percentile. At our selected density of 0.05, there are approximately 17,000 edges in each network. In general, we found evidence of conservation between the *S. cerevisiae *and *S. pombe *networks: co-expression edges between two genes occurred in both networks for 9.2% of the gene pairs that were co-expressed in at least one network. This is about twice the background conservation rate of approximately 4.3%, as determined through comparison to a randomized network produced by a degree-preserving procedure.

To explore the connection between genes predicted to be rewired in the genetic interaction networks and differences between the co-expression networks, rewiring predictions were overlaid on the co-expression networks. Specifically, all non-essential one-to-one orthologs were classified as either rewired or non-rewired based on our prediction of genetic interaction degree (Figure 4b). Using this rewiring labeling, we measured the conservation rate of three types of co-expression edges: co-expression edges connecting two non-rewired genes, connecting two rewired genes, and connecting rewired and non-rewired genes.

We found that co-expression edges involving predicted rewired genes are consistently less conserved than edges with exclusively non-rewired endpoints (Figure 4c), a trend that is robust over different co-expression thresholds used for network sparsification (Figure S5 in Additional file 1). For example, when genes whose degrees differ by 55 interactions or more are considered rewired, 6.9% of the co-expression relationships connecting rewired genes are conserved (107 of 1,659), in contrast to the significantly higher 10.1% of co-expression relationships that are conserved between non-rewired genes (1,238 of 12,472, Fisher's exact test *P *< 10^-6^). This trend grows stronger when considering genes that were predicted to have even larger differences between *S. pombe *and *S. cerevisiae*. This analysis independently confirms predictions of highly rewired genes between the two species and suggests that changes at the level of gene expression regulation are at least one mechanistic factor that contributes to these differences.

## Conclusions

Although individual interactions and gene-specific properties may not be strongly conserved between species, our findings suggest that these properties influence genetic interaction networks in a similar manner. For example, while the genes important for normal growth may vary, the relationship between a gene's fitness contribution and the genetic interactions it exhibits appears to be conserved. Indeed, models trained on both *S.cerevisiae*- and *S.pombe*-derived gene properties were significantly predictive of cross-species genetic interaction degree (Figure 2c), suggesting that the general principles governing genetic interaction network structure are retained through evolution. Thus, a complete genetic interaction network for an organism such as *S. cerevisiae *should serve as a reference network to guide studies to uncover genetic interactions in more complex systems. Predicting specific pairwise interactions across species is of course the next (more difficult) challenge, but models that can accurately predict the variation in number of interactions across the genome provide a foundation for cross-species interaction analysis. Our results also demonstrate that integrative comparisons leveraging multiple functional genomic datasets across species may be one approach to build confidence in differential network analysis. As more data become available, both the extent and nature of network conservation should reveal how functional conservation and divergence can be recognized and utilized in distantly related species.

## Materials and methods

### Gene features

Additional file 2 contains values of gene features for all *S. pombe *genes and Additional file 3 contains values of gene features for all *S. cerevisiae *genes.

### Yeast conservation

Yeast conservation is a count of how many of 23 different species of Ascomycota fungi possess an ortholog of a given gene. This measure was first described in [36], and ortholog data were downloaded from [37]. The 23 species are an expanded set of the 17 species described in the study, with the additions of *Schizosaccharomyces octosporus*, *Schizosaccharomyces japonicus*, *Lodderomyces elongosporus*, *Candida parapsilosis*, *Candida tropicalis*, and *Candida guilliermondii*.

### Broad conservation

Similar, though complementary, to yeast conservation, broad conservation is a count of how many out of a set of 86 non-yeast species possess an ortholog of a given gene. To count this, we obtained orthogroup designations from InParanoid [38]. For each gene, we considered it to have an ortholog in another species only if it appeared in a cluster with the other species and was given a score of 1.0 by the InParanoid clustering method; that is, we considered a yeast gene to have an ortholog in species *x *if it was a seed gene for a gene cluster that had an orthologous cluster in species *x*. Although Ostlund *et al. *[38] considered 100 species, we disregarded the yeast species, since the yeast conservation measure already captures information from these species.

### Codon Adaptation Index

The Codon Adaptation Index, a measure of bias in the usage of synonymous codons, was calculated with the cai tool in the EMBOSS suite [39]. For each gene, the index is based on a comparison between codon frequencies in the gene and frequencies observed in a set of highly expressed genes; for both *S. pombe *and *S. cerevisiae*, EMBOSS included a default codon usage table that was used.

### Copy number

Copy number is a count of the number of paralogs a gene has. This was determined from clusters identified by the InParanoid algorithm [40] run on *S. cerevisiae *and *S. pombe*. All genes that appear in the same cluster were considered copies.

### Disorder

The protein disorder measure is the percent of unstructured residues in a gene's protein product as predicted by the Disopred2 software [41].

### dN/dS

dN/dS is the ratio between nonsynonymous and synonymous mutations in coding regions of genes. For *S. pombe *genes, dN/dS was calculated twice, using *S. japonicus*, *S. octosporus *as out-group species, and averaged to produce a final dN/dS estimate. Orthologous protein sequences were globally aligned with EMBOSS [39] using default parameters. For each *S. pombe *gene, only the out-group ortholog that produced the highest alignment score was used for dN/dS calculations; dN/dS ratios were calculated with the PAML package's implementation of the Yang and Nielsen method for estimating substitution rates [42,43].

Similarly, we computed the average dN/dS ratio for *S. cerevisiae *in comparison to the *sensu strictu *yeast species (*Saccharomyces paradoxus*, *Saccharomyces bayanus *and *Saccharomyces mikatae*). Protein sequences were aligned using MUSCLE [44] and dN/dS ratios were computed using PAML [42].

### Number of domains

The number of domains for a gene is the number of regions that Pfam has identified as domains in the protein sequence of the gene. Domain matches for each protein were obtained online from the Pfam database [45].

### Number of unique domains

Since the same domain is often repeated multiple times in a single protein, this feature modifies number of domains by counting the number of unique domains present in each protein.

### Nc

This measure is a simple statistic of codon usage bias and expresses the effective number of codons used in a gene. The chips tool of EMBOSS [39] was used to calculate this feature.

### Protein length

Protein length is simply the number of amino acids in the corresponding protein.

### Co-expression degree

This measure is derived from the co-expression network, the construction of which is described in its own section. The network contains a level of co-expression for all pairs of genes. We therefore sparsified the network by considering only edges between gene pairs whose co-expression levels were above the 95th percentile. The co-expression degree of a gene is the number of genes with which its co-expression value is retained in this restricted network.

### Expression level

Expression levels of all *S. cerevisiae *genes were downloaded from [46]. Expression levels of all *S. pombe *genes are measured RNAseq abundance that corresponds to [47] and were downloaded from [48].

### Expression variation

We estimated the amount of variability in a gene's expression level by measuring the variance of its expression across a number of different microarray experiments, which included microarray data from different growth conditions and replicates. Within each study, we found each gene's percentile of variation. The final value assigned to each gene is its average percentile across all studies. These datasets were obtained from a number of different studies that deposited data in the Gene Expression Omnibus (GEO) [49]. *S. pombe *data used in this analysis are the same as those used in construction of the *S. pombe *co-expression network.

### Fitness defect

*S. pombe *fitness defect measurements were obtained by conducting a series of control SGA experiments as described elsewhere [2,9,33]. Briefly, a *S. pombe *SGA query strain harboring a dominant drug-resistance marker (*natMX4*) inserted at a neutral genomic locus (*h- *leu1Δ::*natMX4 ade6-M210 ura4-Δ18 leu1-32*) was crossed against the *S. pombe *non-essential deletion mutant collection (*h+ geneX*Δ::*kanMX4 ade6-M210 ura4-Δ18 leu1-32*). Following mating and sporulation, haploid meiotic progeny harboring both the *kanMX4 *and *natMX4 *markers are selected and colony sizes are measured after applying standard normalization procedures. We have previously shown that colony sizes derived from these control screens reflect fitness defect of the *kanMX4*-marked single mutant strains that comprise the deletion mutant array. Fitness estimates were based on four control screens as described above and combined with five mutant screens (prz1, res2, SPAC1687.22c, SPCC1682.08, and SPAC6G9.14), which contained the dominant drug-resistance marker (*natMX4*) [9].

*S. cerevisiae *fitness defect values, defined quantitatively in [6], were published in [5] and experimental procedures are detailed in [33]. As in the *S. pombe *protocol described above, SGA was used to insert a neutral query marker into mutant strains so that we could observe colony growth for each mutant in the deletion collection under the effects of only the single deletion. Fitness estimates are based on a large number of replicate screens.

### Protein-protein interaction degree

The protein-protein interaction degree of each gene's protein is the number of physical interactions reported in BioGRID, version 2.0.58 [50]. Interactions considered physical were restricted to those identified by the following terms: Affinity Capture-MS, Affinity Capture-RNA, Affinity Capture-Western, Biochemical Activity, Co-crystal Structure, Co-fractionation, Co-localization, Co-purification, Far Western, FRET, PCA, Protein-peptide, Protein-RNA, Reconstituted Complex, and Two-hybrid.

### Multifunctionality

Multifunctionality is a measure of the number of GO terms that are annotated to a gene [26]. From GeneDB [51] and Saccharomyces Genome Database [52] gene association files (download in November 2009) for *S. pombe *and *S. cerevisiae*, respectively, redundant terms - one term from pairs of terms that are considered 'alternative ids' - were removed before totaling the number of GO term annotations for each gene.

### Genetic interaction degrees

Negative genetic interaction degrees of *S. pombe *genes were derived from interactions reported in [10] (Additional file 2). Only those interactions with S-scores ≤ -2.5 were considered. This dataset contains 551 genes that are involved in chromosome function; intentionally included are approximately 100 genes that participate in processes present in both *S. pombe *and human, but importantly, are not present in *S. cerevisiae *(for example, RNA interference machinery).

Negative genetic interaction degrees of *S. cerevisiae *genes (Additional file 3) were collected from the measurements reported in [5], which screened for interactions involving 3,456 array genes, 1,438 of which have *S. pombe *orthologs. As suggested by the authors, only negative interactions with an epsilon value of ≤ -0.08 and a *P*-value cutoff < 0.05 were considered. This dataset includes degree measurements for most non-essential genes.

### Orthologs

Orthology mappings (Additional files 4 and 5) are from the InParanoid eukaryotic ortholog database [24]. Although the InParanoid algorithm produces clusters, our analysis depends on ortholog pairs. To calculate correlations between *S. cerevisiae *and *S. pombe *for each of the gene features (Figure 2a), only genes in one-to-one orthology mappings were used. When holding out orthologs for degree prediction in a set of 'species-specific' genes (Figure 2c), all genes that had any number of orthologs were removed. Since InParanoid may not report orthologs that other algorithms have detected, we took a conservative approach by additionally removing any genes that had an ortholog in the pombe database GeneDB [53], which includes manually curated orthologs.

### Models and evaluation

Our models are bagged regression trees that use the 16 features described above (Additional file 6). Breiman [23] suggests that using an ensemble of only 25 classifiers can result in nearly all improvement gains that bagging can produce over a single classifier; however, we used 100 trees because the computation required in training is relatively low and we were interested in analyzing the tree structures. Individual trees were trained by MATLAB's classregtree function, which minimizes node impurity according to mean squared error. For each tree, a bootstrap sample was used to select, with replacement, a set of training genes the same size as the set of total genes (therefore each tree is trained on approximately 63.2% of all genes) and held out genes. The final prediction for a single gene of the species used to train the model (that is, the within-species prediction) is the median of all predictions from trees for which the gene was not in the training set (Additional file 7). The final prediction for a gene of the species not used to train the model (that is, the cross-species prediction) is the median of predictions from all trees (Additional file 8).

To assess the performance of the model, we calculated the Pearson correlation coefficient between predicted and actual degrees of genes with known degrees. To estimate stability of performance, we repeated the model construction and evaluation 25 times and reported predictive ability as the mean Pearson correlation coefficient and its standard deviation across all 25 repetitions for within- and cross-species cases (Figure 2c).

### *S. pombe *genetic interaction screens

Eight whole-genome *S. pombe *genetic interaction screens were completed using the method described in [9]. The query strains were deletion mutants for each of the following genes: SPCC1682.08c, SPBC21D10.12, SPBC13E7.09, SPAC4G8.13c, SPAC3A11.13, SPAC27D7.13c, SPAC22F3.09c, SPAC16A10.07c. The resulting double mutant colonies were processed as described in [6]. Negative interactions were derived from the scores by applying an interaction cutoff of ≤ -0.08 and a *P*-value cutoff of < 0.05. Degree measurements were then derived for all non-essential genes by counting the number of significant interactions across the set of eight queries (Additional file 9). Significant correlation with the predicted degrees was also observed when a stricter cutoff was applied (interaction score ≤ -0.12, *P*-value < 0.05 yielded a correlation r = 0.41, *P*-value < 10^-117^).

### Rewiring groups and significance assessment

To make comparisons between degrees of orthologs in the genetic interaction networks of the two yeast species, we considered genetic interaction degree to be predicted percentile for all *S. pombe *genes, while percentiles of actual degrees were used for *S. cerevisiae*.

To search for groups of functionally related genes that have been rewired since the divergence of *S. pombe *and *S. cerevisiae*, we defined gene groups in two ways. The first simply grouped genes whose protein products form a complex in a set of complexes defined in [6] (Additional file 10). The number of proteins per complex ranges from 2 to 81, with the vast majority having 6 or fewer proteins.

The second method for making sets of functionally related genes grouped genes that share a biological process GO term annotation [26] (Additional file 11). We considered GO terms that are annotated to greater than 3 and fewer than 50 genes in either of the two species. Additionally, a group of *S. cerevisiae *genes was required to have a minimum number of two genes with known genetic interaction degrees; a group of *S. pombe *genes was required to have a minimum of two genes with known fitness defect. Since GO terms tend to be highly redundant, we filtered gene groups so that no pair of groups overlapped by more than 50% of either group's genes.

To determine orthologous pairs of groups that have significantly different average degrees, we calculated the difference between the median degrees of genes in each species' group, and then compared the differences to a distribution of differences produced from randomly grouped genes. We generated this background by creating groups of randomly selected genes in one species, then identifying orthologous groups in the other species composed of the selected genes' orthologs. A query gene-group pair was compared to a background containing only random gene-group pairs whose group sizes were identical to the query groups. For example, a protein complex of five individual *S. cerevisiae *proteins may contain four genes that have *S. pombe *orthologs; this query gene-group pair would be compared with a background of groups with five random *S. cerevisiae *genes matched with a group of four of their *S. pombe *orthologs.

### Comparative analysis of co-expression networks

To independently validate genetic interaction degree differences across species, we performed a comparative analysis of co-expression networks of *S. cerevisiae *and *S. pombe *genes. The *S. cerevisiae *network was previously published [34] and is based on integration of a large collection of expression datasets. To construct the *S. pombe *network (Additional file 12), data from nine expression studies were collected from the GEO database [54] (Additional file 13). Genes with missing values for more than 30% of the samples were removed, and the remaining missing values in each dataset were imputed using KNNImpute [55]. Datasets reflecting probe intensities (rather than relative ratios) were log-transformed. After processing, the nine *S. pombe *expression datasets were integrated as described in [34,56]. The naive Bayes approach for dataset integration requires a gold standard set of positives, for which we used direct gene co-annotation to any term in the GO that contained between 2 and 100 genes. *S. pombe *gene annotations were downloaded from the GO website [26,57] in May 2011. All analysis and integration of expression data were completed using the Sleipnir library [56].

We applied a 95th percentile cutoff to edges in both the *S. cerevisiae *and *S. pombe *co-expression networks, such that only the highest scoring 5% of edges were retained.

To estimate the overlap between the *S. cerevisiae *and *S. pombe *networks in the absence of biological conservation, we randomized the edges of the *S. cerevisiae *network and considered the background conservation to be the overlap between this randomized network and the *S. pombe *network. The randomizing procedure repeatedly chose two random edges and exchanged an endpoint of one edge with an endpoint of the other edge, thus maintaining the degrees of genes in the network. The number of endpoint swaps performed was 20 times the number of edges in the network, which is a sufficient number of swaps to remove the original relationships between genes.

## Abbreviations

GEO: Gene Expression Omnibus; GO: Gene Ontology; NETO: new end take off; SGA: Synthetic Genetic Array.

## Competing interests

The authors declare that they have no competing interests.

## Authors' contributions

EK, JB, CLM, MC, CB and BJA conceived the study and planned the analysis. EK and JB gathered all gene features for both species and carried out all predictive modeling and cross-species comparison. GC, KC-R and MC performed the *S. pombe *interaction screens and fitness measurements. RD constructed the co-expression networks for *S. pombe*. EK, MC, GD, CB and CLM wrote the manuscript. All authors read and approved the final manuscript.

## Supplementary Material

Additional file 1 **Supplemental figures**. Supplemental figures and legends are given. Click here for file

Additional file 2 **Gene features of *S. pombe *genes**. Gene features and observed genetic interaction degrees are given for all *S. pombe *genes. Click here for file

Additional file 3 **Gene features of *S. cerevisiae *genes**. Gene features and observed genetic interaction degrees are given for all *S. cerevisiae *genes. Click here for file

Additional file 4 **Orthology mapping from *S. pombe *genes to *S. cerevisiae *genes**. Orthologs obtained from InParanoid and GeneDB for each *S. pombe *gene are given. Click here for file

Additional file 5 **Orthology mapping from *S. cerevisiae *genes to *S. pombe *genes**. Orthologs obtained from InParanoid and GeneDB for each *S. cerevisiae *gene are given. Click here for file

Additional file 6 **Regression trees**. Regression trees trained on bootstrap samples of *S. cerevisiae *gene features and negative genetic interaction degree are pictured. Click here for file

Additional file 7 **Negative genetic interaction degree predictions for *S. cerevisiae *genes**. Predictions of negative genetic interaction degree produced by each of the 100 regression trees that were trained on *S. cerevisiae *data are given for each *S. cerevisiae *gene. For a gene, a predicted degree is given from each tree for which the gene was held out from training; otherwise, NaN indicates that the gene was used for training. Columns correspond to trees pictured and ordered in Additional file 6. Click here for file

Additional file 8 **Negative genetic interaction degree predictions for *S. pombe *genes**. Predictions of negative genetic interaction degree produced by each of the 100 regression trees that were trained on *S. cerevisiae *data are given for each *S. pombe *gene. Columns correspond to trees pictured and ordered in Additional file 5. Click here for file

Additional file 9 **Genetic interaction degrees of non-essential *S. pombe *genes**. The number of genes that interact with each non-essential *S. pombe *gene are given. The eight queries that were screened for genetic interactions with non-essential genes are SPCC1682.08c, SPBC21D10.12, SPBC13E7.09, SPAC4G8.13c, SPAC3A11.13, SPAC27D7.13c, SPAC22F3.09c, and SPAC16A10.07c. Click here for file

Additional file 10 **Cross-species degree comparisons of protein complexes**. Orthologous sets of genes, the predicted and observed average degrees of the sets, and the results of our cross-species comparison are given. Click here for file

Additional file 11 **Cross-species degree comparisons of genes annotated by GO terms**. Orthologous sets of genes, the predicted and observed average degrees of the set, and the results of our cross-species comparison are given. GO terms are prefixed by a '(p)' or '(c)' to indicate that the term originated from *S. pombe *or *S. cerevisiae *annotations, respectively. Click here for file

Additional file 12 **The *S. pombe *co-expression network**. The co-expression network is symmetric and is represented as a comma-delimited lower triangle of a matrix. Click here for file

Additional file 13 ***S. pombe *GEO co-expression studies**. The listed co-expression studies were used to construct a co-expression network. Citations are for original publications. Click here for file
