# Supplementary material for: Conserved rules govern genetic interaction degree across species
Source: Genome Biol. 2012 Jul 2;13(7):R57. doi: 10.1186/gb-2012-13-7-r57 (PMC3491379; doi:10.1186/gb-2012-13-7-r57)
Supplement: Additional file 1 — Supplemental figures. Supplemental figures and legends are given. [file gb-2012-13-7-r57-S1.PDF]

Supplementary figures

Conserved rules govern genetic interaction degree across species

Elizabeth Koch (koch@cs.umn.edu)[1]
Michael Costanzo (michael.costanzo@utoronto.ca)[2,3]
Jeremy Bellay (bellay@umiacs.umd.edu)[1,4]
Raamesh Deshpande (rdeshpan@cs.umn.edu)[1]
Kate Chatfield-Reed (kechatfi@cpsc.ucalgary.ca) [5]
Gordon Chua (gchua@ucalgary.ca)[5]
Gennaro D'Urso (GDUrso@med.miami.edu)[6]
Brenda Andrews (brenda.andrews@utoronto.ca)[2,3]
Charles Boone (charlie.boone@utoronto.ca)[2,3]
Chad L. Myers, corresponding author, (cmyers@cs.umn.edu)[1]

[1]Department of Computer Science and Engineering, University of Minnesota, 200 Union Street SE, Minneapolis, MN 55455, USA.

[2]Banting and Best Department of Medical Research, Terrence Donnelly Centre for Cellular and Biomolecular Research, University of Toronto, 160 College Street, Toronto, Ontario M5S 3E1, Canada.

[3]Department of Molecular Genetics, Terrence Donnelly Centre for Cellular and Biomolecular Research, University of Toronto, 160 College Street, Toronto, Ontario M5S 3E1, Canada.

[4]Institute for Advanced Computer Studies, University of Maryland College Park, 3115 Biolmolecular Sciences Bldg #296, College Park, MD 20742, USA.

[5]Institute of Biocomplexity and Informatics, Department of Biological Sciences, University of Calgary, 2500 University Drive NW, Calgary, AB T2N 1N4 Canada.

[6]Department of Molecular and Cellular Pharmacology, University of Miami School of Medicine, P.O. Box 016189, Miami, FL 33101, USA.

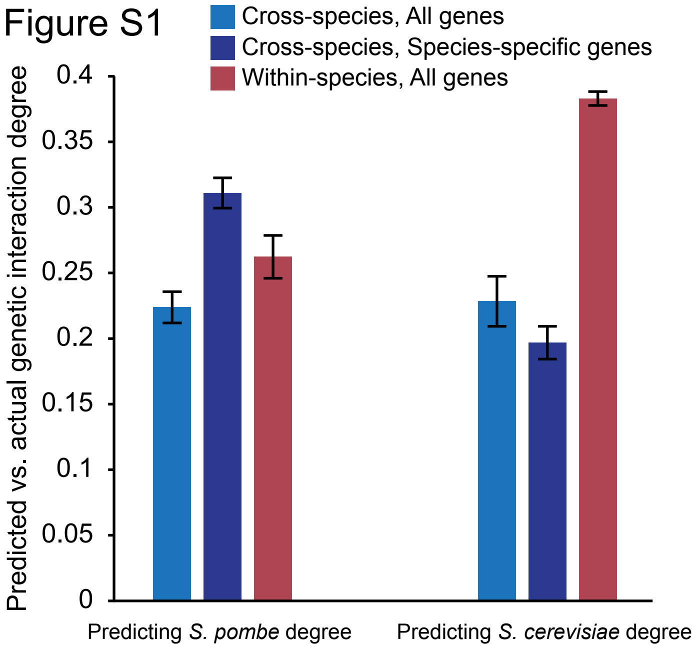

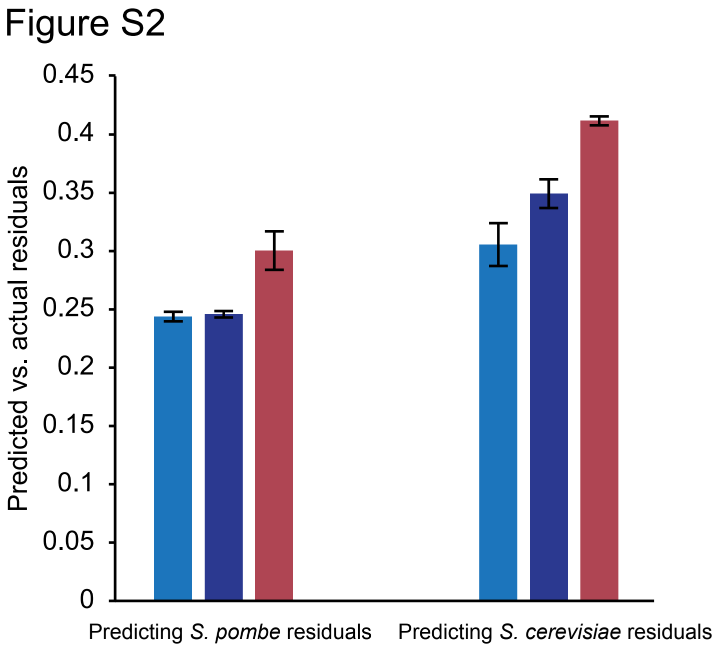

Figure S1. Evaluation of prediction performance excluding the SM fitness defect feature from bagged regression tree models. Models were trained on all features listed in Table 1 *except* for SM fitness defect. Pearson correlation coefficients between predicted and actual negative genetic interaction degrees were averaged across 25 repetitions of model construction, shown here with error bars of standard deviation. The left set of bars shows the performance of predictions made for ~550 *S. pombe* genes and the right set of bars shows the performance of predictions made for all non-essential deletion mutants in *S. cerevisiae*. For each scenario, models were trained both on data from the same species (red bar) as well as data from the other species (blue bars). The light blue bars correspond to predicting degrees of all genes in the test species, while the dark blue bars correspond to predicting the subset of genes lacking orthologs in the training species.

Figure S2. Patterns in gene features other than SM fitness defect show predictive ability not captured by SM fitness defect alone. Using all features except SM fitness defect, models were trained to predict the residual negative genetic interaction degree that remained after subtracting degree predictions made from a regression tree model that was trained on the single feature SM fitness defect. Details of this bar chart are the same as those specified above for Figure S1.

Figure S3

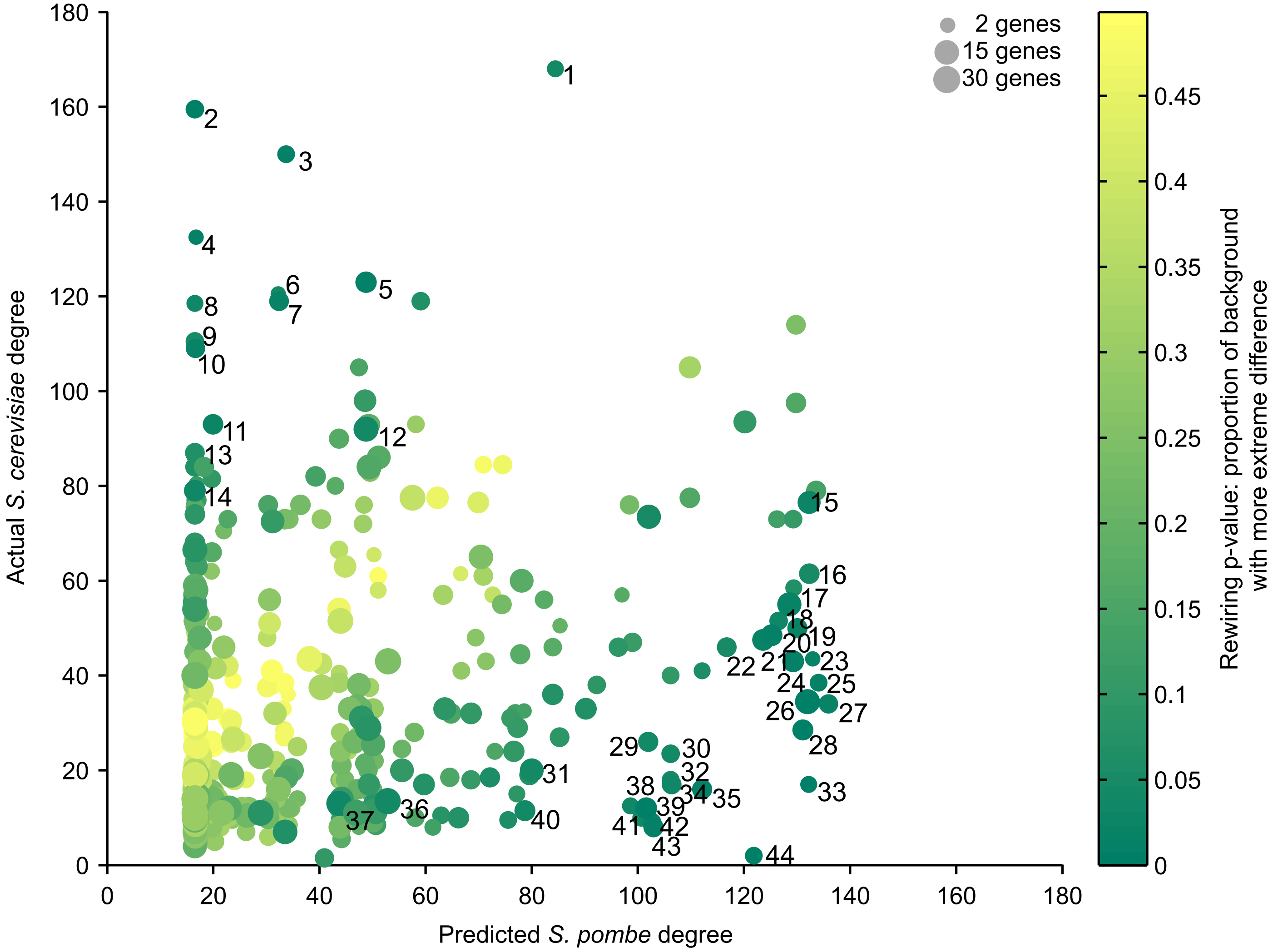

1 (c) histone ubiquitination
2 (p) phospholipid dephosphorylation
3 (p) cytokinesis checkpoint
4 (c) mitochondrial DNA metabolic process
5 (c) histone exchange
6 (p) cellular response to osmotic stress
7 (p) regulation of calcium−mediated signaling
8 (p) proton−transporting ATP synthase complex assembly
9 (p) positive regulation of transcription from RNA polymerase II promoter, meiotic
10 (p) protein insertion into membrane raft
11 (c) establishment of mitotic spindle orientation
12 (c) histone deacetylation
13 (c) acetyl−CoA biosynthetic process from pyruvate
14 (c) hyperosmotic response
15 (p) CVT pathway
16 (p) meiotic gene conversion
17 (c) double−strand break repair via homologous recombination
18 (p) positive regulation of histone methylation
19 (c) mitochondrial electron transport, ubiquinol to cytochrome c
20 (p) mating type switching
21 (c) ribosomal small subunit assembly
22 (p) regulation of cellular response to stress
23 (c) regulation of nuclease activity
24 (c) double−strand break repair via single−strand annealing
25 (c) meiotic DNA double−strand break processing
26 (c) CVT pathway
27 (c) C−terminal protein lipidation
28 (c) meiotic DNA double−strand break formation
29 (c) nucleotide−excision repair, DNA damage recognition
30 (c) L−serine biosynthetic process
31 (c) adaptation of signaling pathway by response to pheromone involved in conjugation with cellular fusion
32 (c) donor selection
33 (c) positive regulation of lipid metabolic process
34 (c) regulation of lipid biosynthetic process
35 (c) response to hexose stimulus
36 (c) lipid transport
37 (p) response to reactive oxygen species
38 (p) regulation of intracellular protein kinase cascade
39 (p) positive regulation of protein modification process
40 (c) maintenance of cell polarity
41 (p) phospholipid translocation
42 (c) activation of protein kinase activity
43 (p) chromatin silencing at rDNA
44 (c) pyrimidine salvage

Figure S3. Global analysis of rewiring based on whole-genome predictions in *S. pombe.* Points in the scatter plot each represent groups of between two and 23 genes that are annotated with the same GO term (see Methods in main text). Darker color represents complexes that are predicted to have significant rewiring. Generally, genes in GO-term groups that fall on the diagonal are predicted to have conserved degrees, while those that fall far off-diagonal show evidence for large degree differences between the two species. Significantly rewired groups are labeled by their GO terms.

Figure 4A

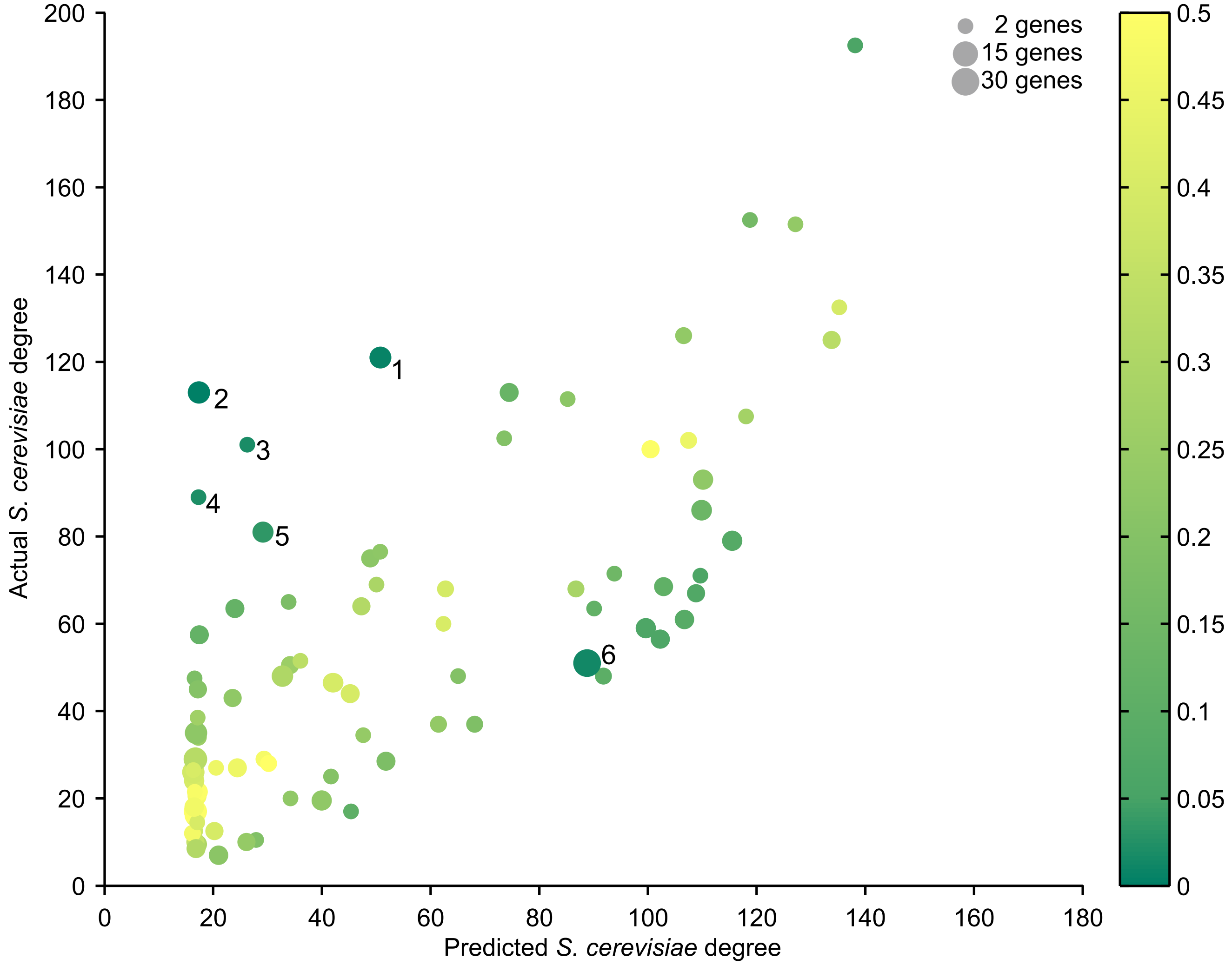

1 Swr1p complex
2 Rpd3L complex
3 prohibitin complex
4 ubiquitin conjugating enzyme complex
5 Set3p complex
6 cytoplasmic ribosomal small subunit

Figure 4B

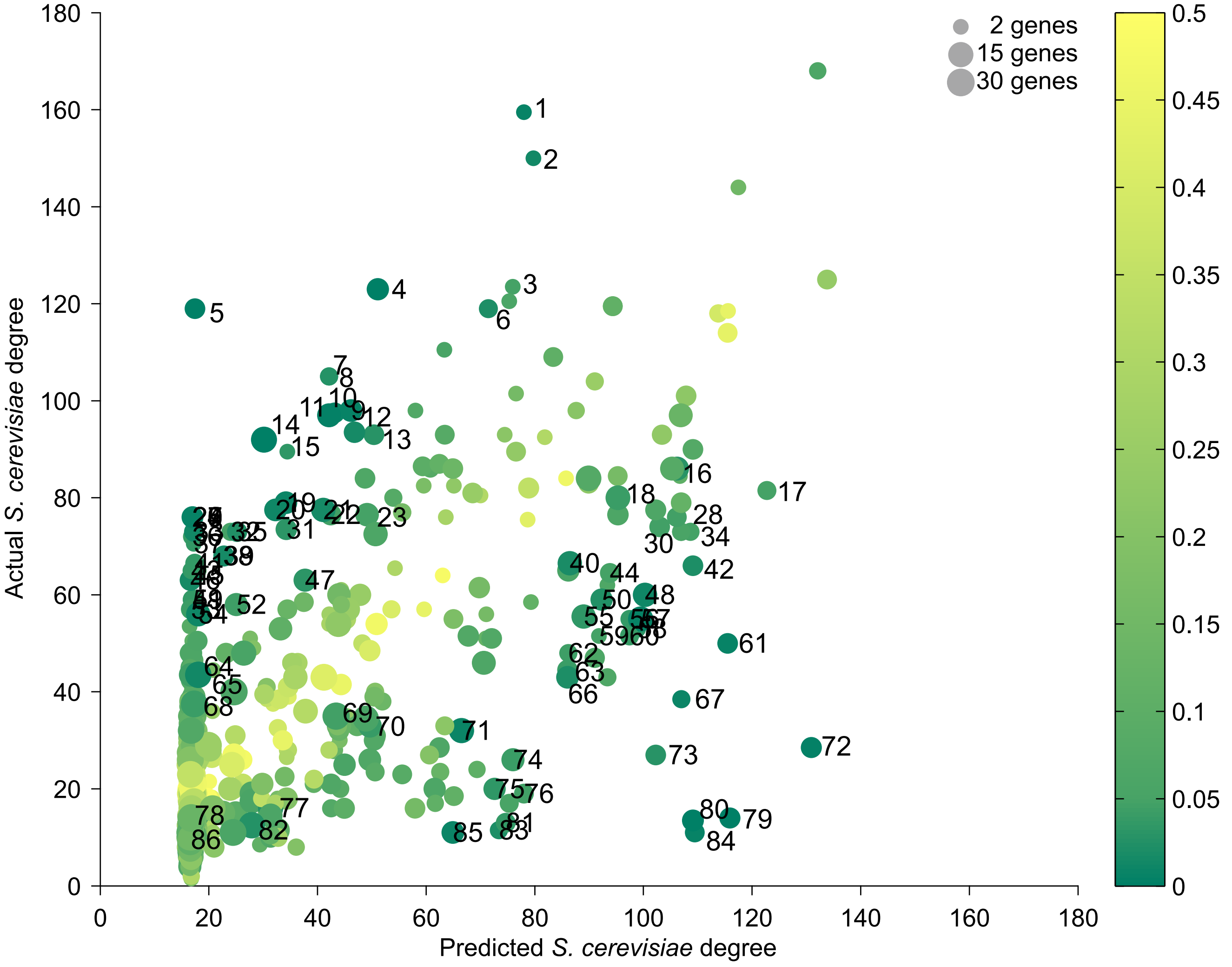

1 (p) phospholipid dephosphorylation
2 (p) cytokinesis checkpoint
3 (p) mitotic spindle elongation
4 (c) histone exchange
5 (p) regulation of calcium−mediated signaling
6 (c) barrier septum formation
7 (c) positive regulation of intracellular protein kinase cascade
8 (c) regulation of MAPKKK cascade
9 (p) positive regulation of cell communication
10 (c) positive regulation of response to stimulus
11 (p) protein amino acid deacetylation
12 (p) chromatin silencing at telomere
13 (c) establishment of mitotic spindle orientation
14 (c) histone deacetylation
15 (c) phosphatidylinositol biosynthetic process
16 (p) retrograde transport, endosome to Golgi
17 (c) polyamine biosynthetic process
18 (c) retrograde transport, endosome to Golgi
19 (c) hyperosmotic response
20 (p) regulation of transcription, mitotic
21 (p) chromatin silencing at centromere
22 (p) protein insertion into membrane
23 (p) CVT pathway
24 (p) mRNA export from nucleus in response to heat stress
25 (p) stress−activated MAPK cascade
26 (p) cellular sodium ion homeostasis
27 (p) response to arsenic
28 (p) DNA synthesis involved in DNA repair
29 (c) autophagic vacuole assembly
30 (p) protein targeting to peroxisome
31 (p) chromatin silencing at silent mating−type cassette
32 (p) negative regulation of transcription from RNA polymerase II promoter, mitotic
33 (p) endoplasmic reticulum unfolded protein response
34 (p) meiotic DNA double−strand break formation
35 (c) DNA replication−independent nucleosome assembly
36 (p) regulation of cell shape
37 (c) protein homooligomerization
38 (c) protein insertion into ER membrane
39 (c) response to arsenic
40 (p) peroxisome organization

41 (c) regulation of ubiquitin homeostasis
42 (p) pantothenate metabolic process
43 (c) septin checkpoint
44 (c) protein import into peroxisome matrix, docking
45 (c) misfolded or incompletely synthesized protein catabolic process
46 (c) protein insertion into membrane
47 (c) nuclear migration along microtubule
48 (c) double−strand break repair via nonhomologous end joining
49 (p) protein retention in ER lumen
50 (p) double−strand break repair via homologous recombination
51 (c) protein retention in ER lumen
52 (p) microtubule−based movement
53 (p) autophagic vacuole assembly
54 (c) endoplasmic reticulum unfolded protein response
55 (c) protein import into peroxisome matrix
56 (p) DNA replication−dependent nucleosome assembly
57 (c) double−strand break repair via homologous recombination
58 (p) negative regulation of translation
59 (p) positive regulation of histone methylation
60 (c) DNA replication−dependent nucleosome assembly
61 (c) mitochondrial electron transport, ubiquinol to cytochrome c
62 (p) regulation of chromatin silencing at centromere
63 (c) heteroduplex formation
64 (c) postreplication repair
65 (c) cellular response to organic substance
66 (c) double−strand break repair via single−strand annealing
67 (c) meiotic DNA double−strand break processing
68 (c) response to biotic stimulus
69 (c) microautophagy
70 (p) protein deubiquitination
71 (c) protein deubiquitination
72 (c) meiotic DNA double−strand break formation
73 (c) flocculation via cell wall protein−carbohydrate interaction
74 (c) response to salt stress
75 (c) karyogamy involved in conjugation with cellular fusion
76 (c) phospholipid dephosphorylation
77 (p) dicarboxylic acid metabolic process
78 (p) cellular amino acid derivative metabolic process
79 (c) polyamine metabolic process
80 (c) pantothenate metabolic process
81 (c) sulfur amino acid transport
82 (c) cellular amino acid derivative biosynthetic process
83 (c) FAD transport
84 (c) isocitrate metabolic process
85 (c) aromatic amino acid family biosynthetic process
86 (p) nicotinamide nucleotide metabolic process

Figure S4. Within-species control for cross-species rewiring analysis. The rewiring-discovery procedure was applied to *S. cerevisiae* genes and their predicted and actual genetic interaction degrees (substituting *S. cerevisiae* predictions for *S. pombe* predictions in the rewiring-discovery pipeline). (A) This within-species evaluation revealed six out of 91 complexes that appeared significantly rewired ($p < 0.05$). While this is fewer than was identified in the *S. pombe*-*S. cerevisiae* comparison, this is more than expected by chance, which likely reflects or complexes for which we are systematically over- or under-predicting actual degrees. Of the 11 rewired complexes (Figure 4A), four of these are among the six complexes significant in the control experiment. (B) 14 of the 44 predicted rewired GO terms in the *S. pombe*-*S. cerevisiae* comparison (Figure S3) also showed significance in the within-species control. We suggest that these cases should be excluded from further analysis, as they likely reflect systematic prediction errors, not true cases of cross-species differences.

Figure S5

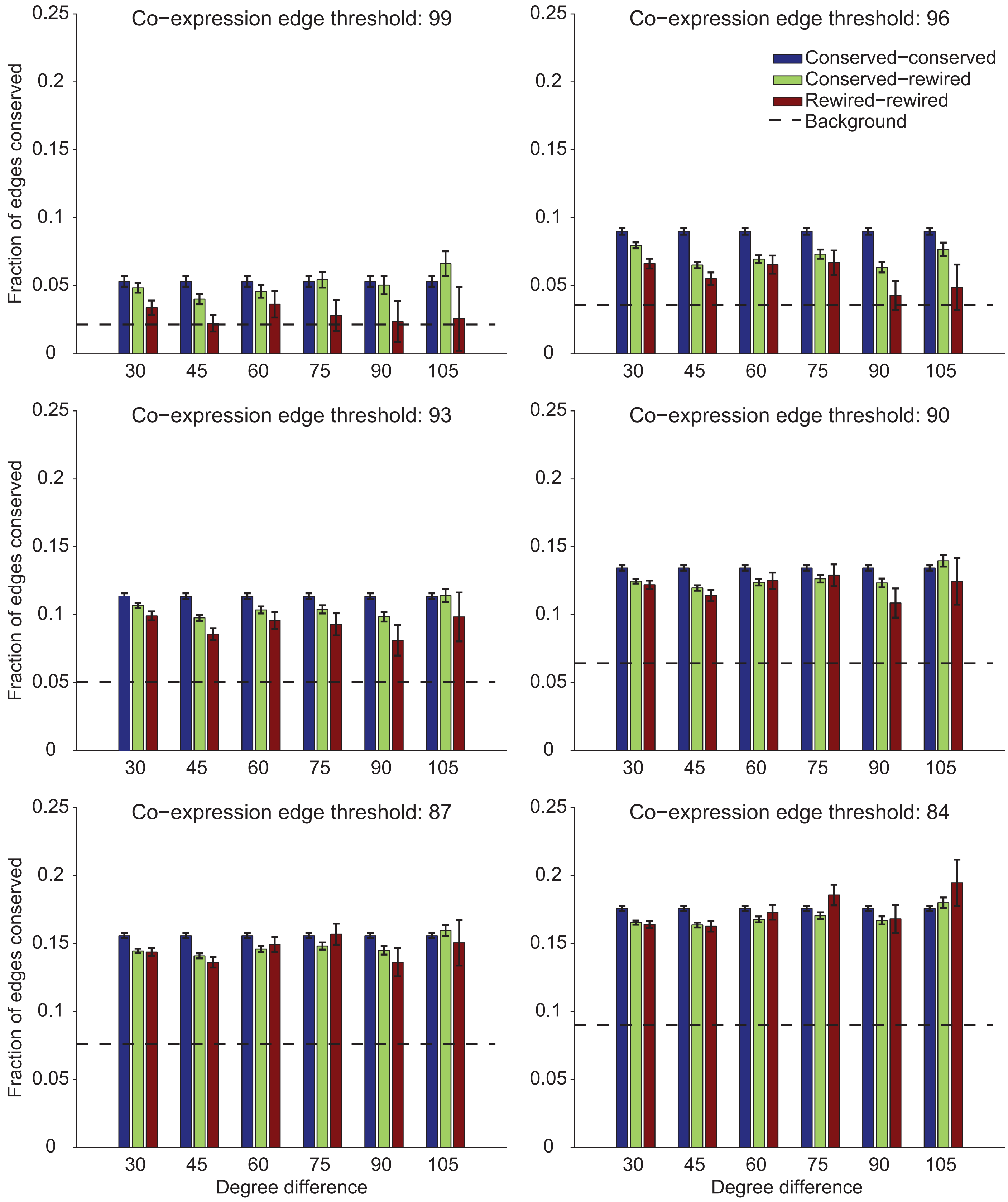

Co−expression edge threshold: 99
Co−expression edge threshold: 96
Co−expression edge threshold: 93
Co−expression edge threshold: 90
Co−expression edge threshold: 87
Co−expression edge threshold: 84
Conserved−conserved
Conserved−rewired
Rewired−rewired
Background
Fraction of edges conserved
Degree difference
0
0.05
0.1
0.15
0.2
0.25
30
45
60
75
90
105

Figure S5. Validation of rewiring predictions is robust across a range of co-expression percentile thresholds used to define networks. As described in the main text and Figure 4, we constructed networks of co-expression relationships among genes for each yeast species, then labeled genes according to our rewiring designation. Edges in the co-expression network were classified by whether involved genes were both rewired, only one was rewired, or neither was rewired. Bars show fractions of conserved co-expression relationships between species within each of these classes and errorbars are 95% confidence intervals for the binomial proportion test. Panels show conservation results given from co-expression networks that differ in their confidence and density, both of which are affected by placing a threshold, which is a percentile, on co-expression levels and retaining only edges corresponding to levels that exceed the threshold. Note that we observe a significant difference between the conserved-conserved and rewired-rewired classes for a range of cutoffs. Also, the significance of the difference diminishes for weaker thresholds, likely due to an abundance of spurious co-expression edges allowed at these cutoffs.
