# Supplementary figures and images for: Conserved rules govern genetic interaction degree across species
Source: Genome Biol. 2012 Jul 2;13(7):R57. doi: 10.1186/gb-2012-13-7-r57 (PMC3491379; doi:10.1186/gb-2012-13-7-r57)

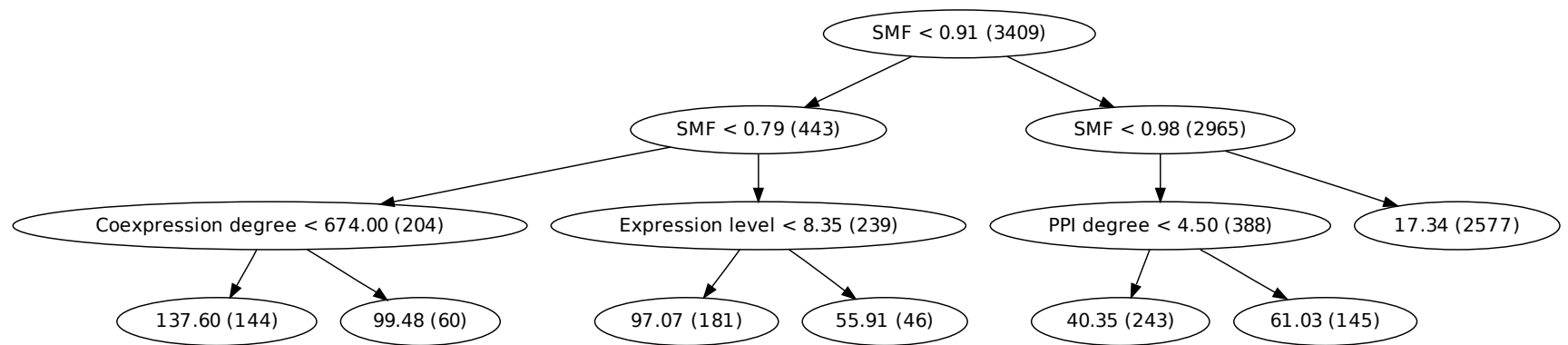

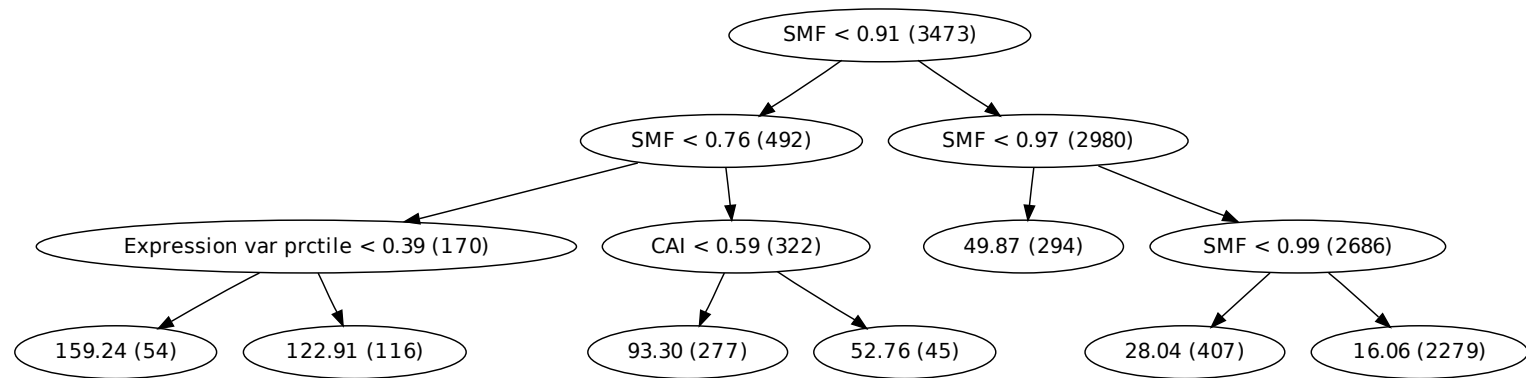

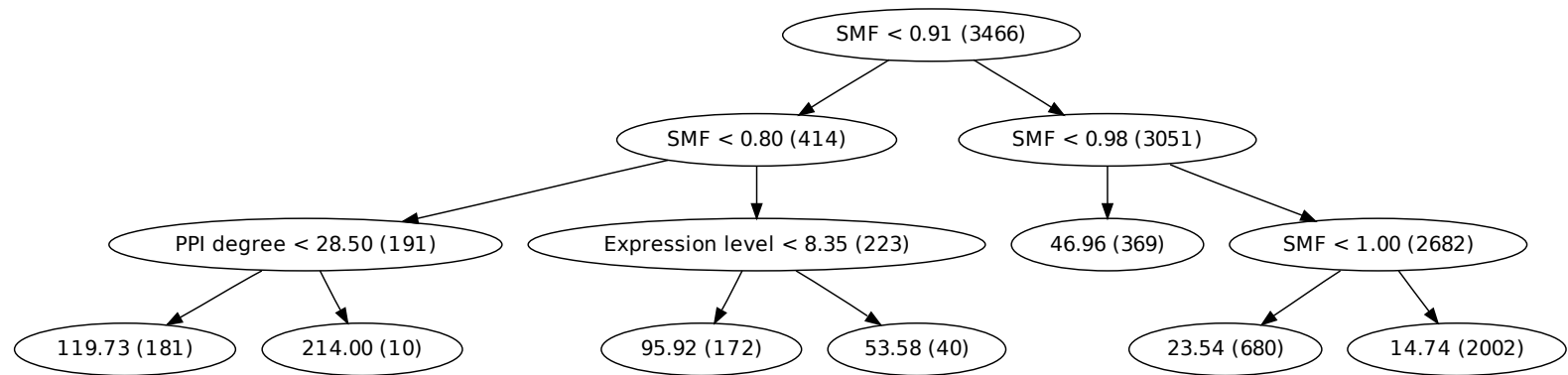

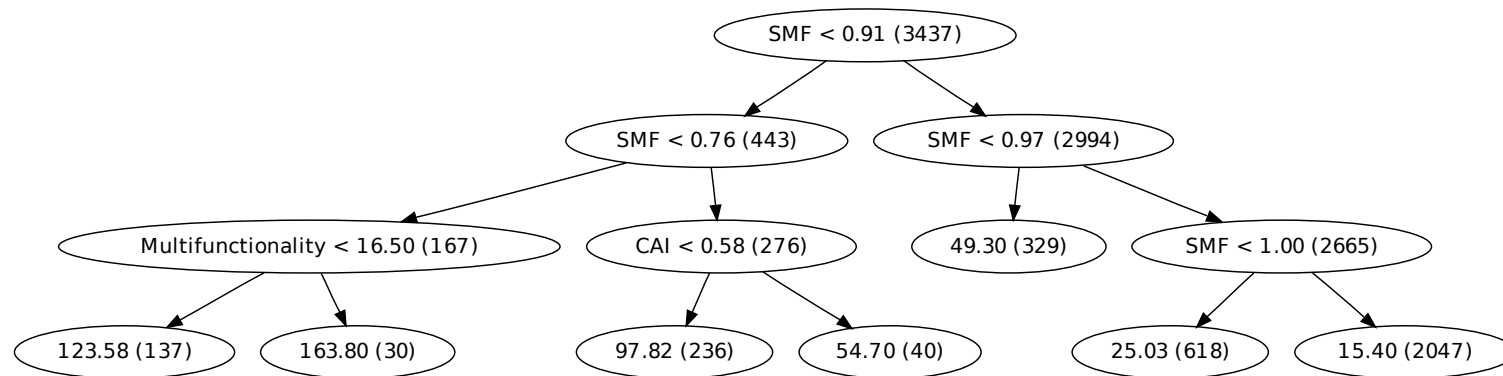

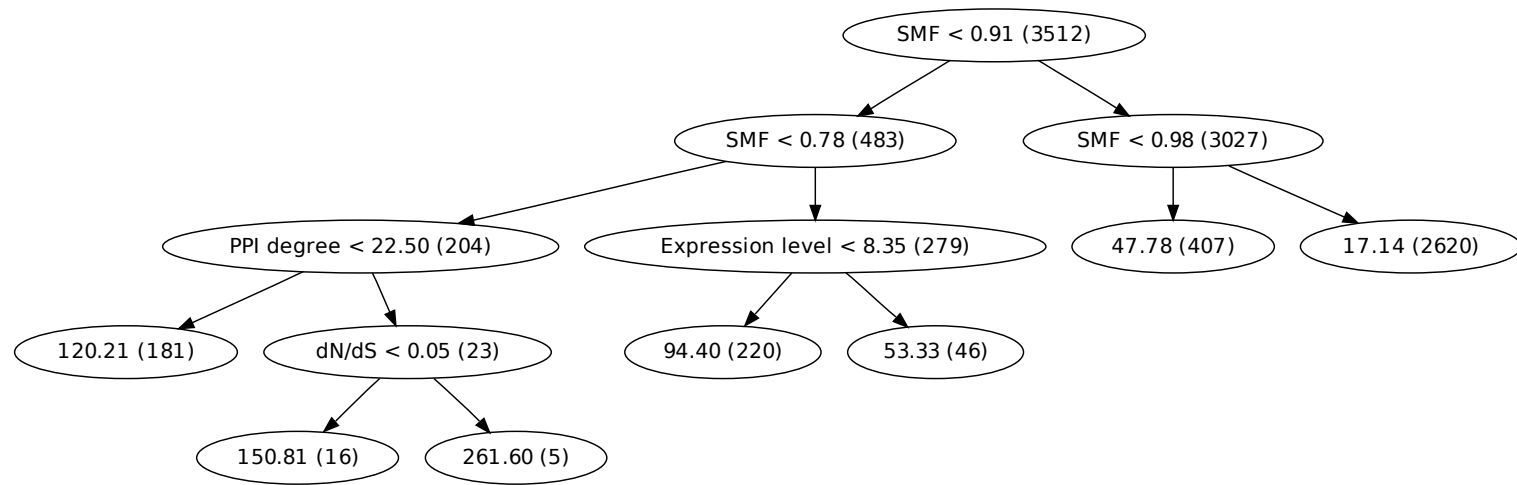

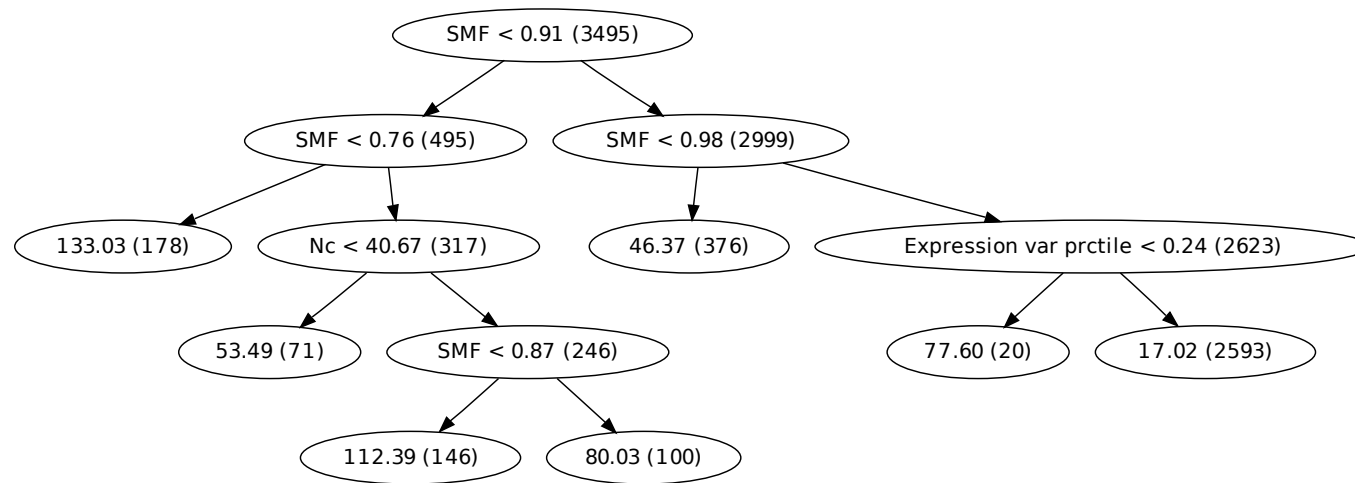

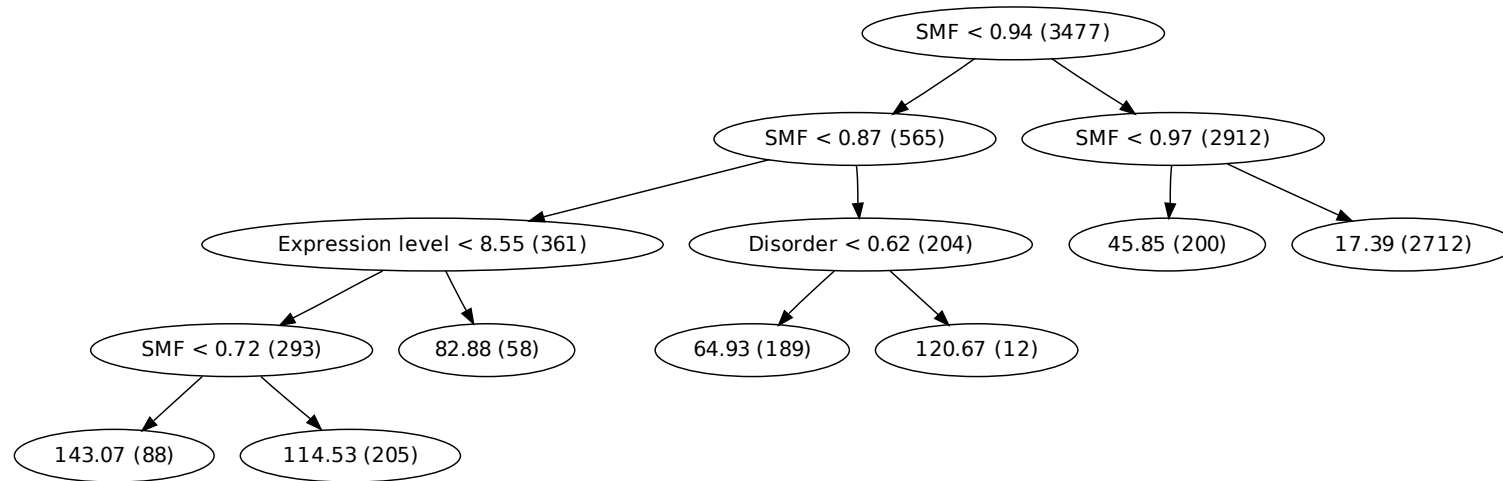

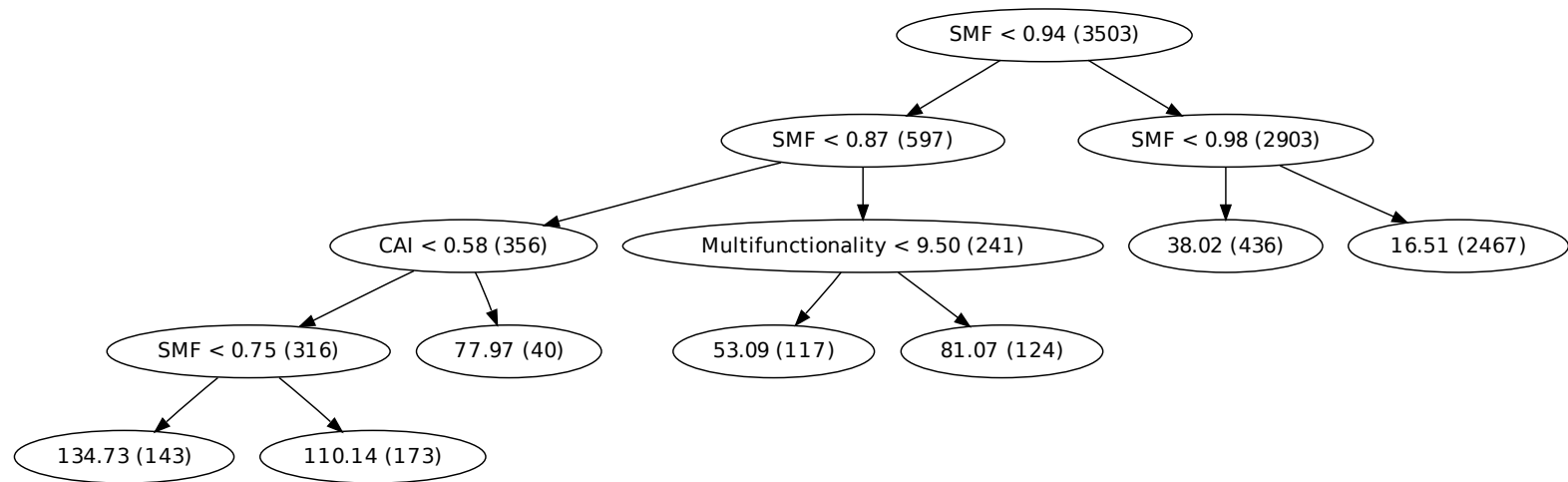

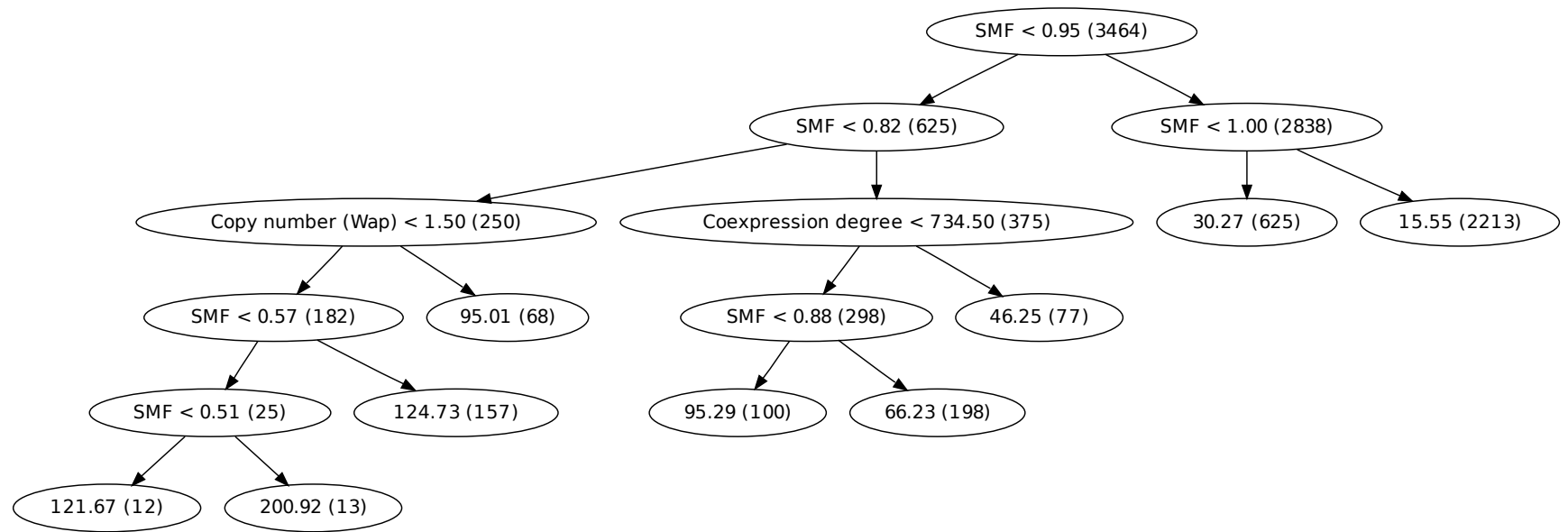

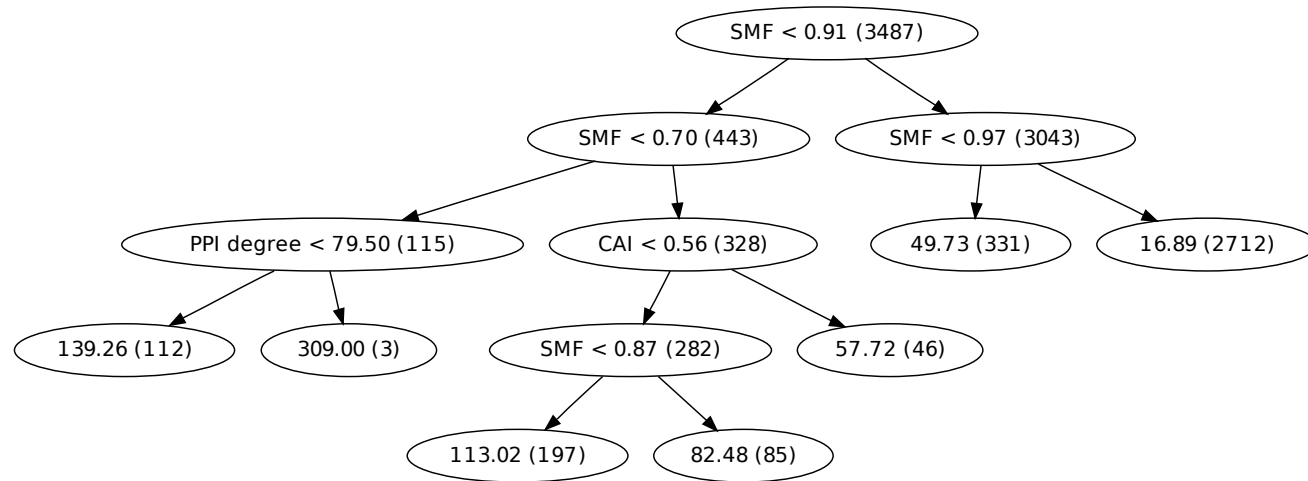

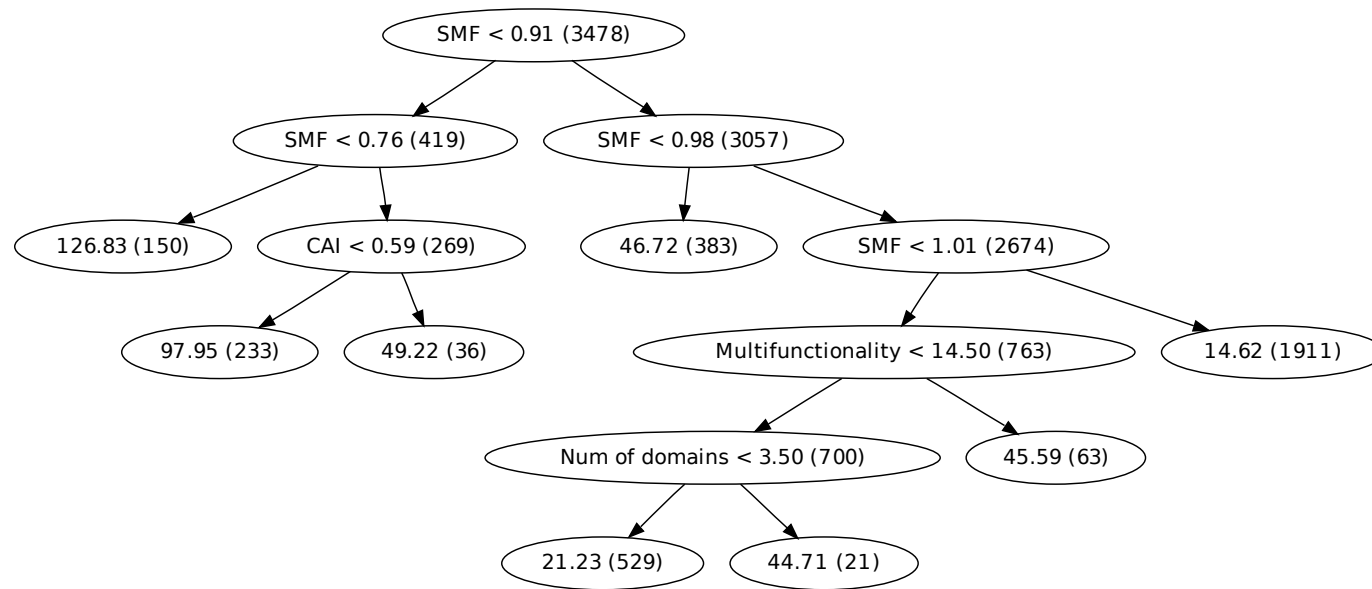

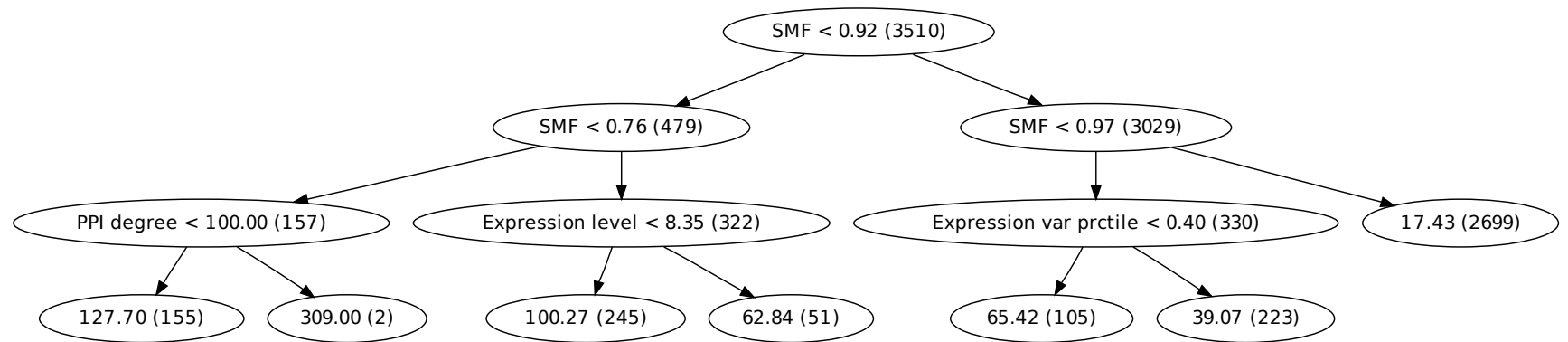

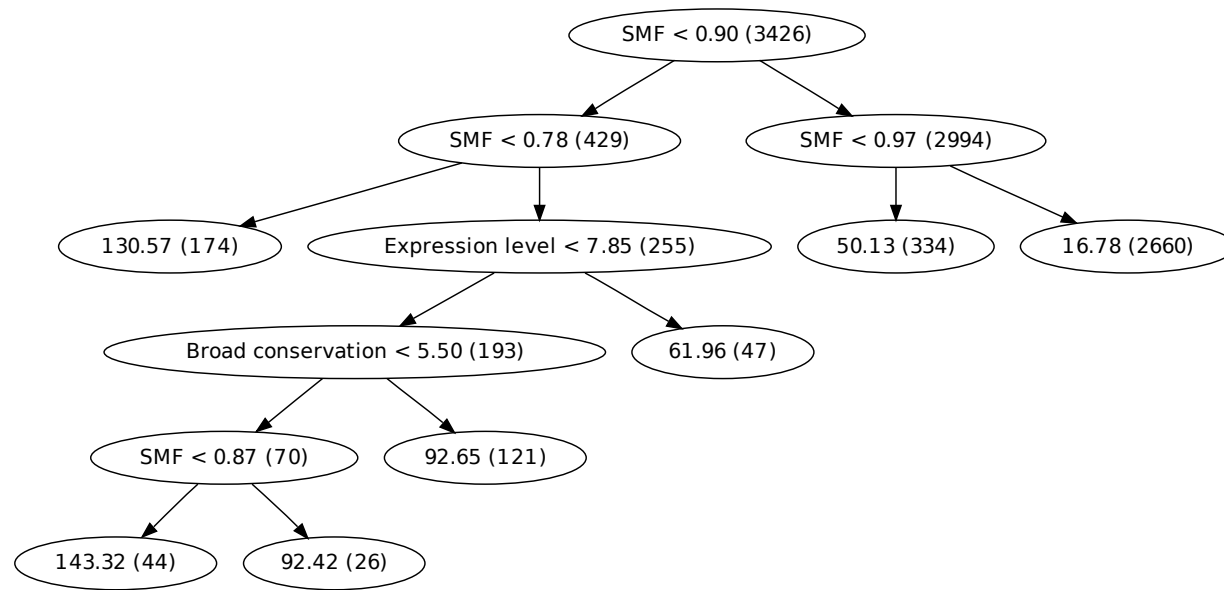

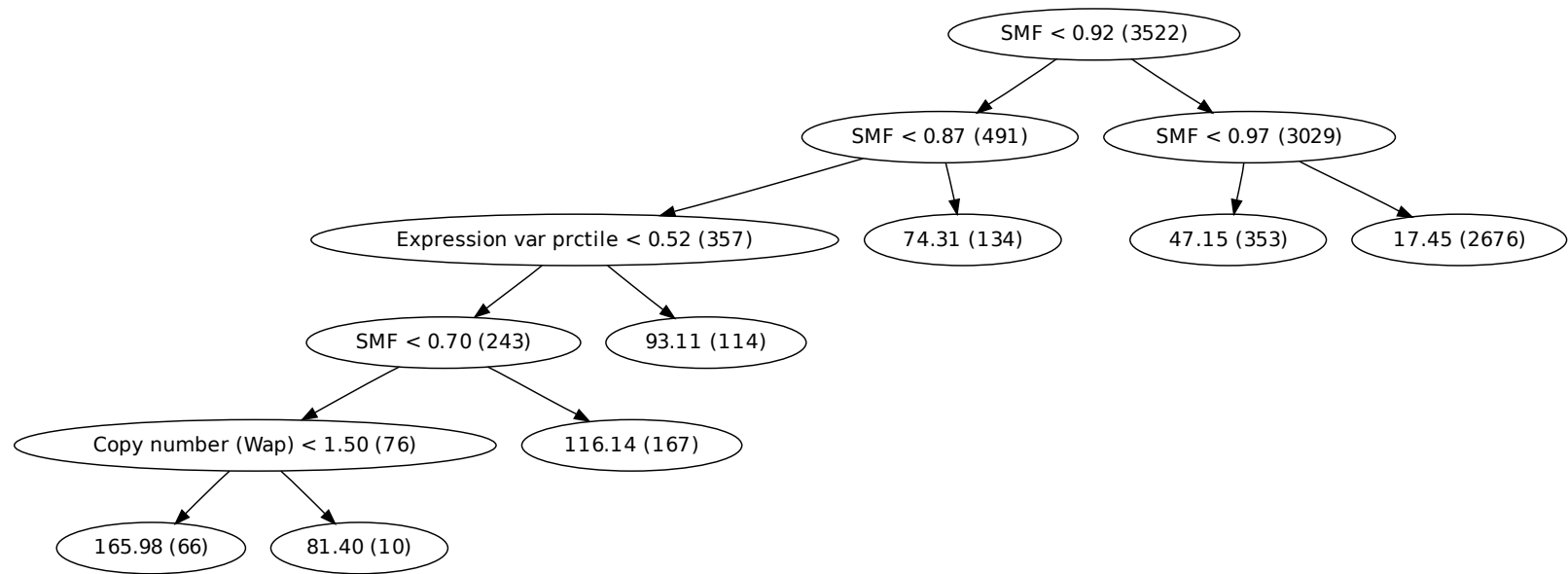

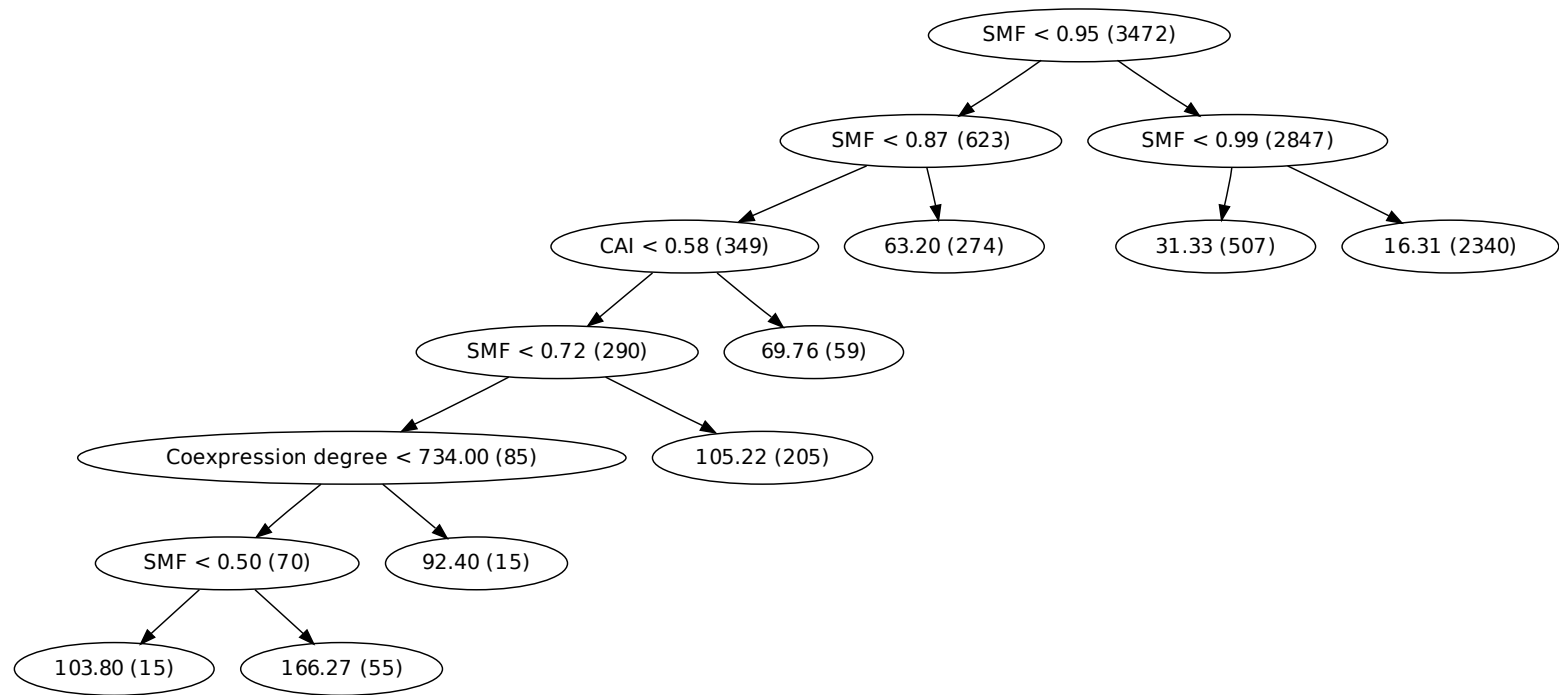

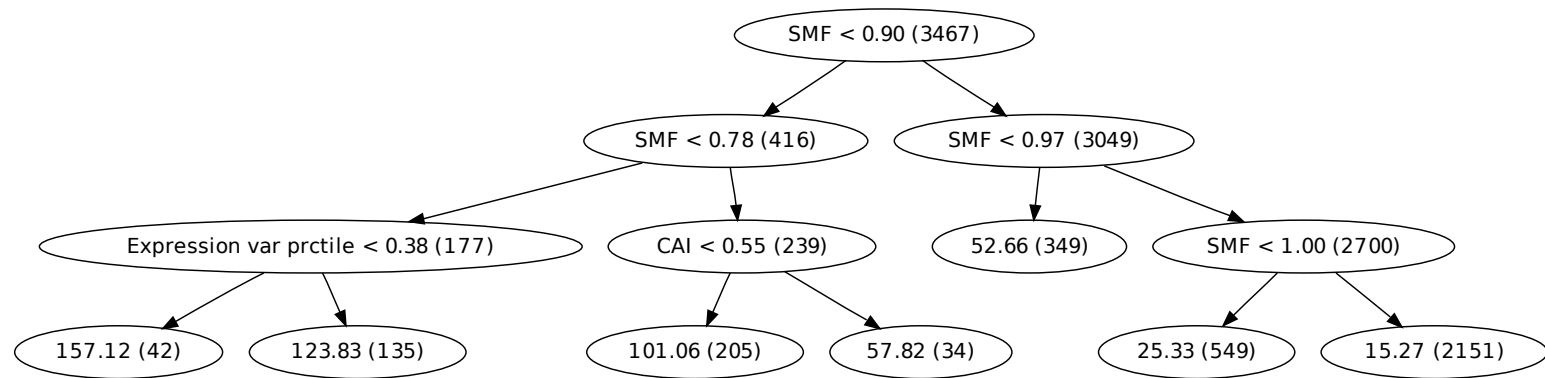

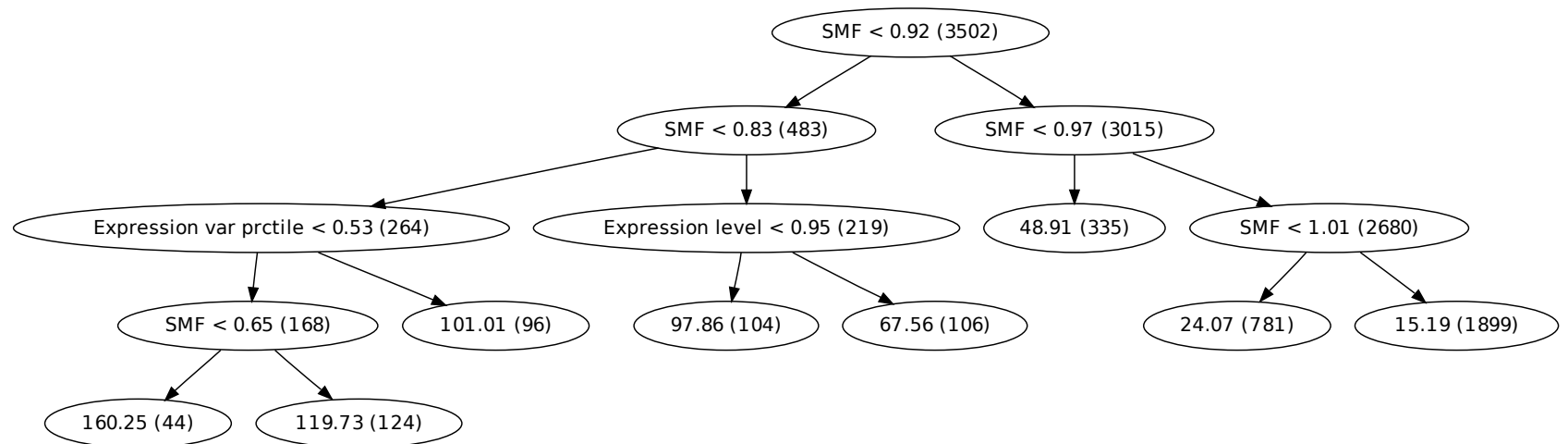

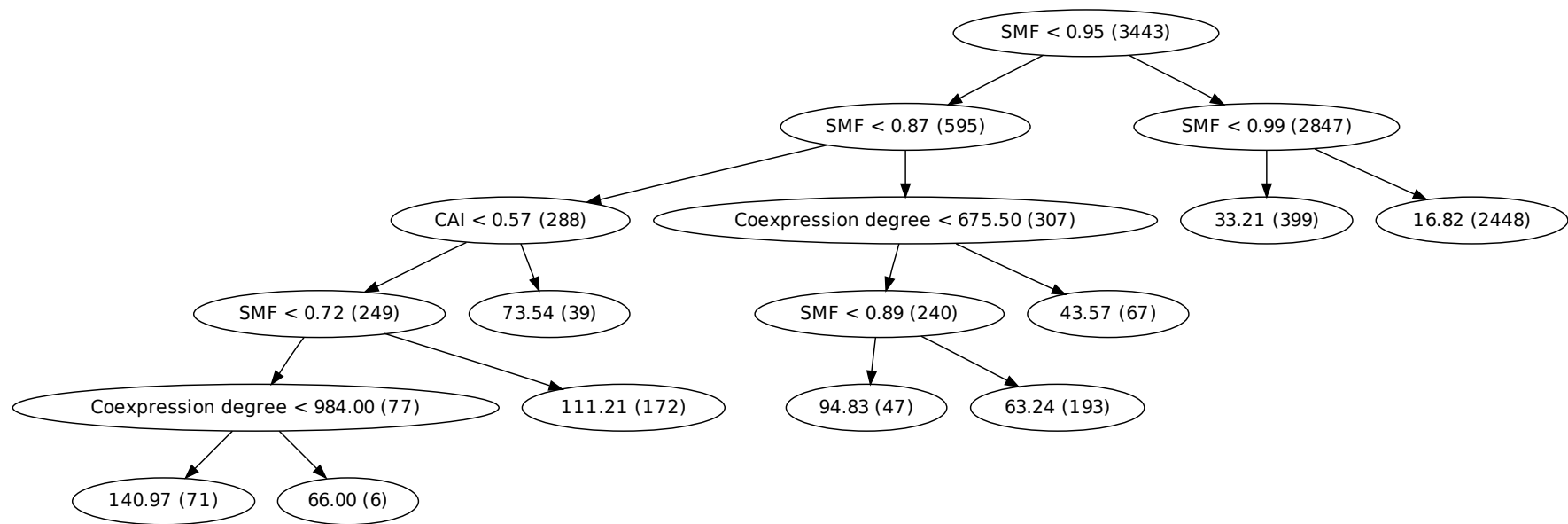

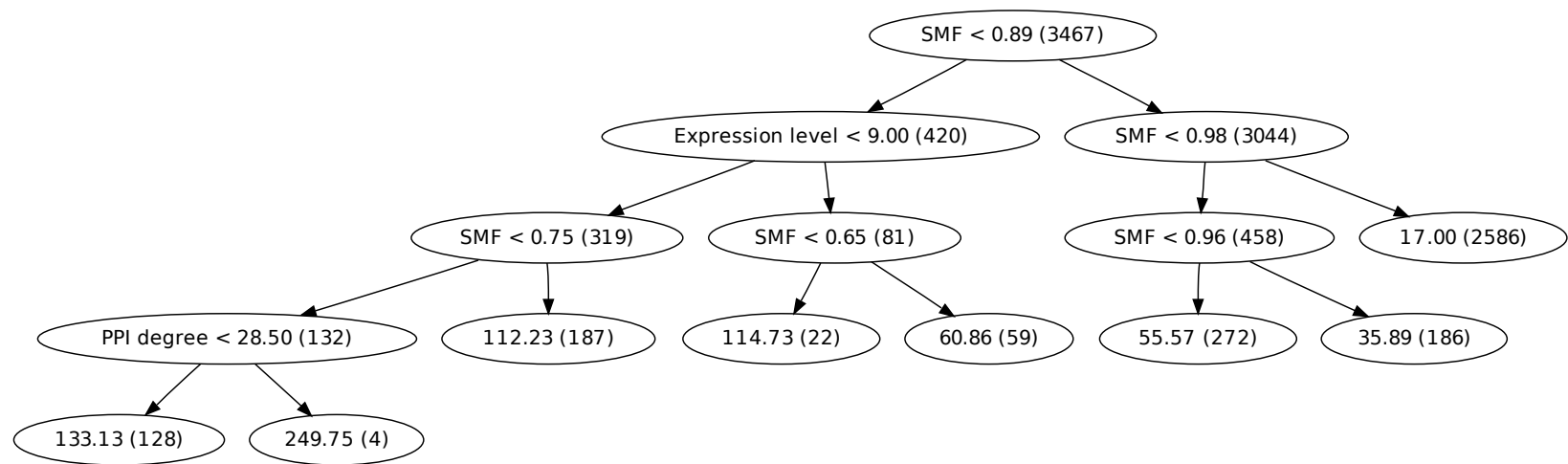

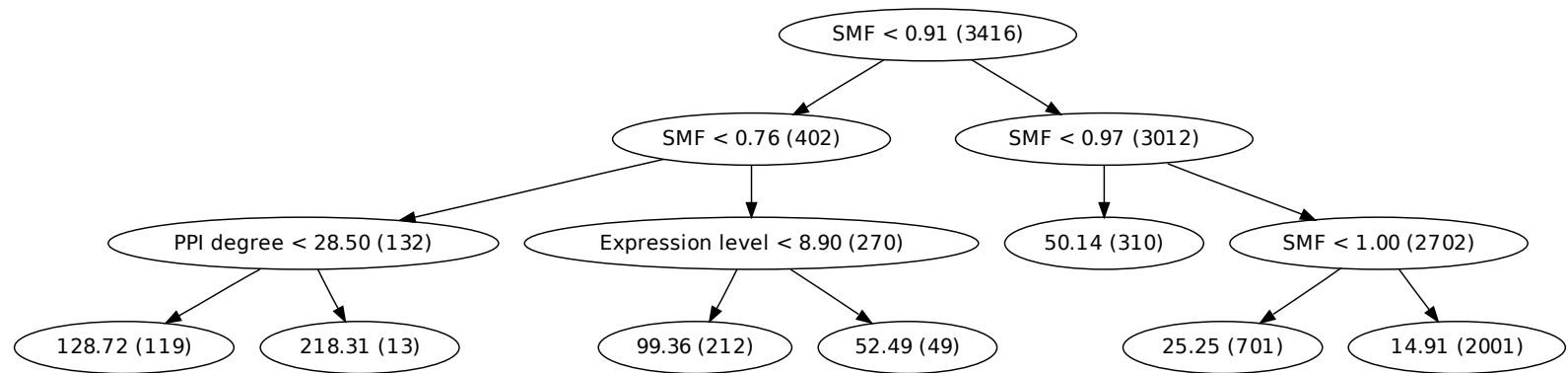

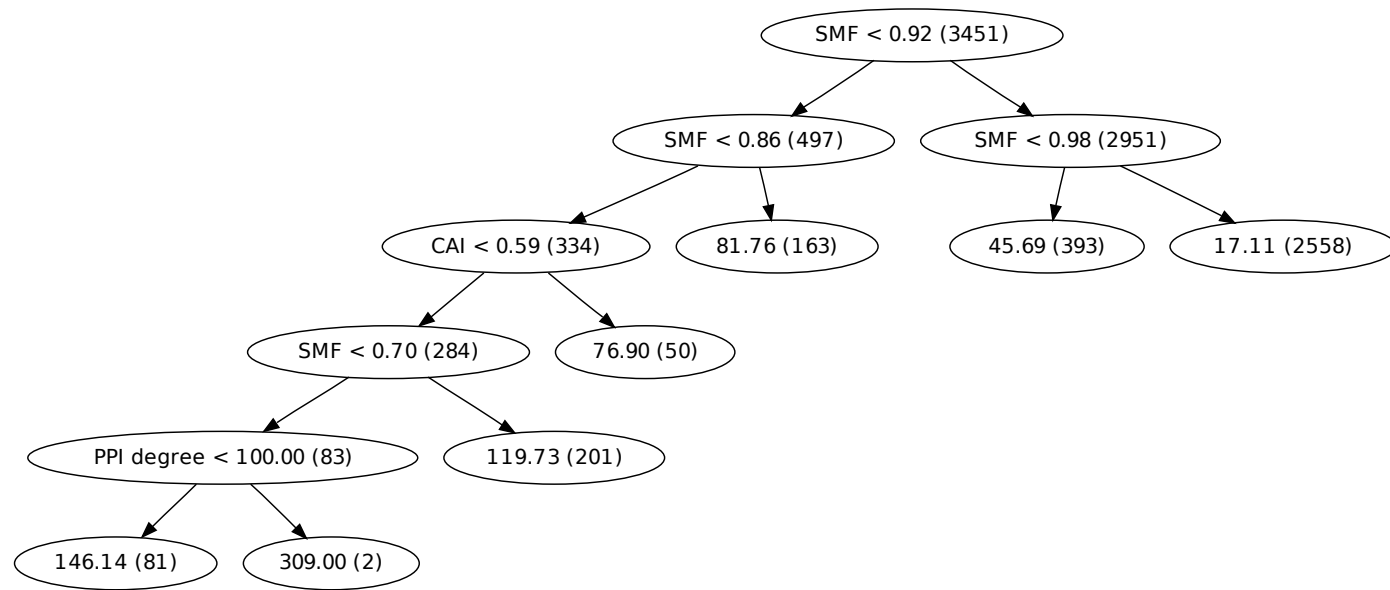

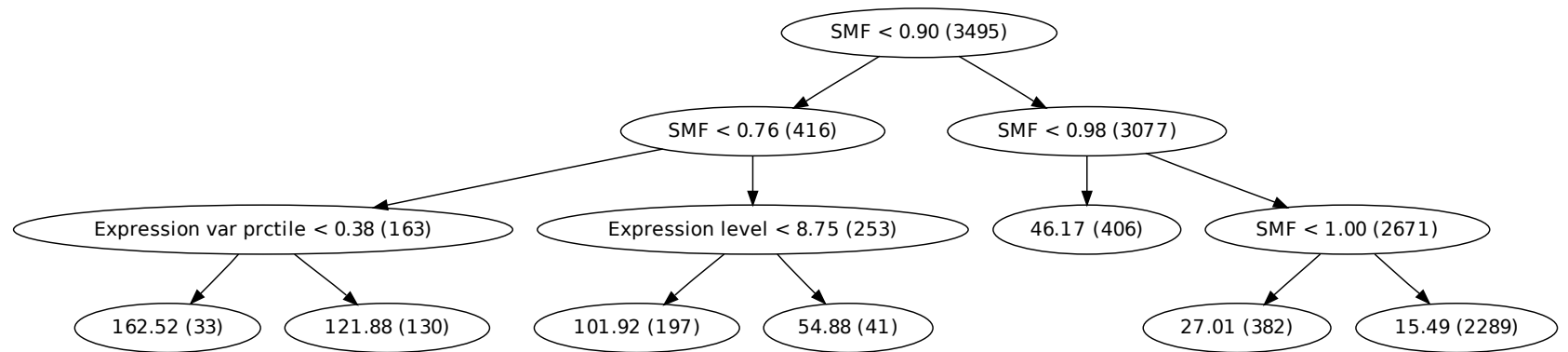

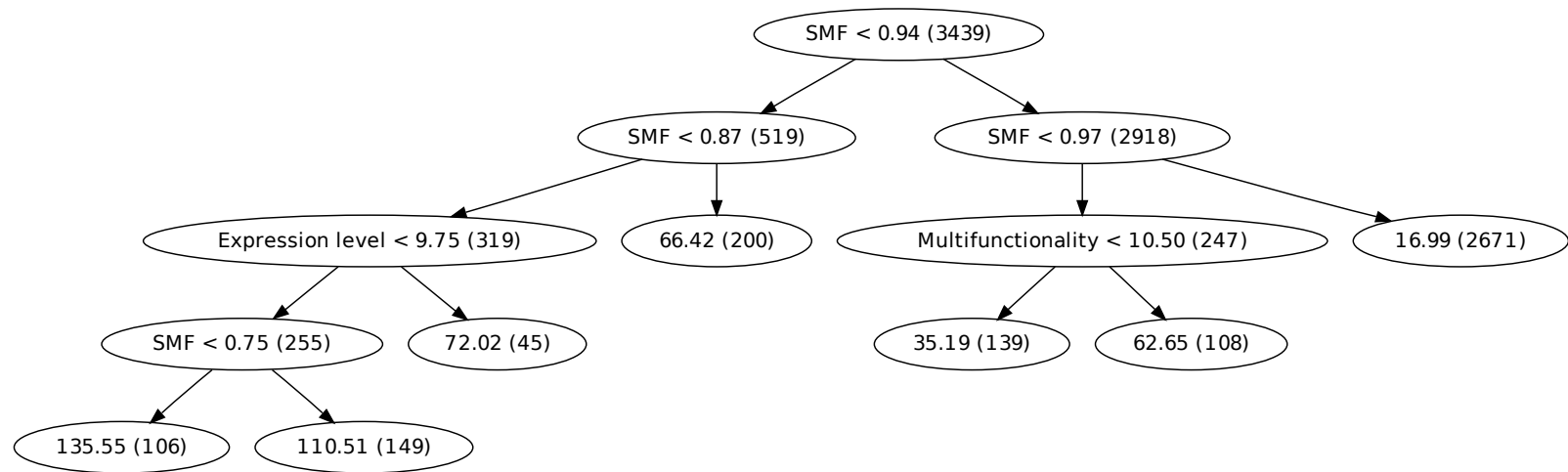

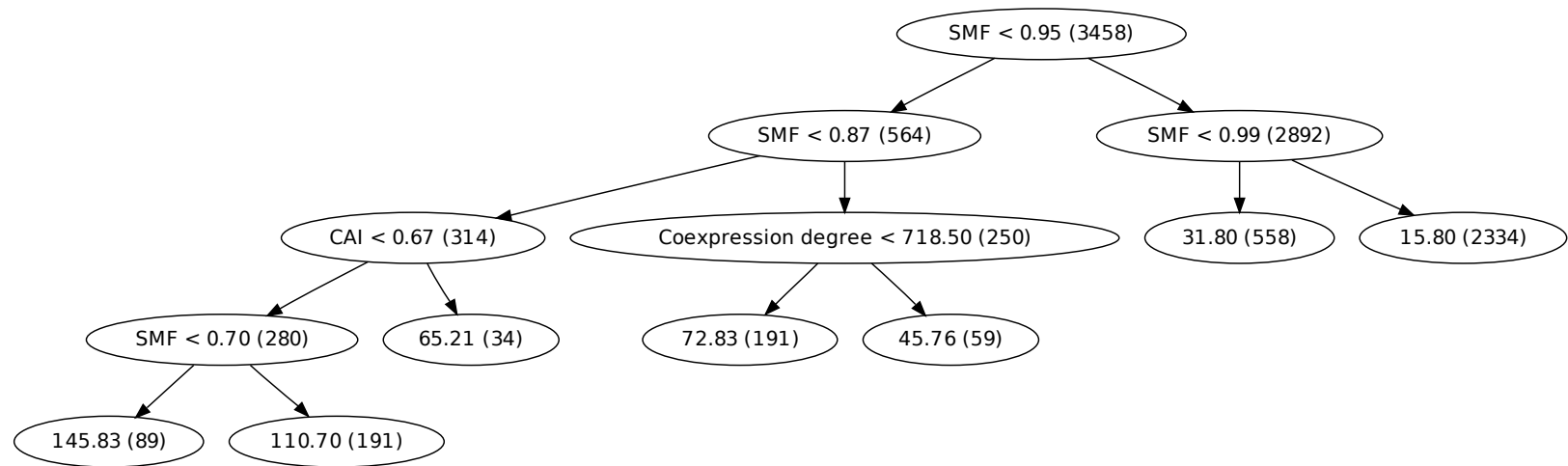

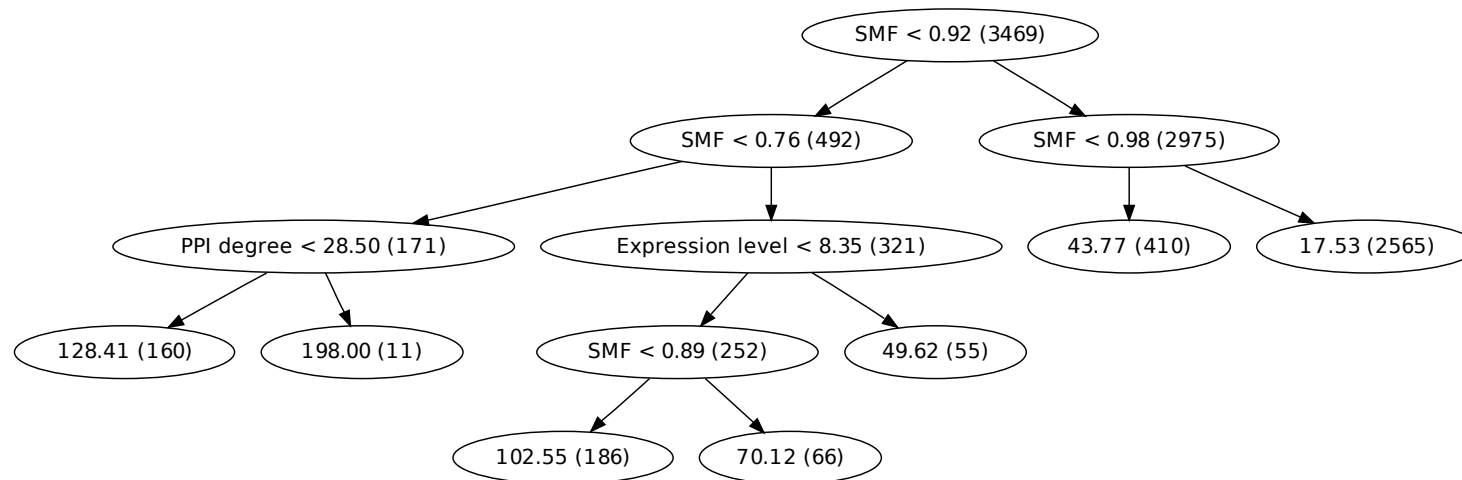

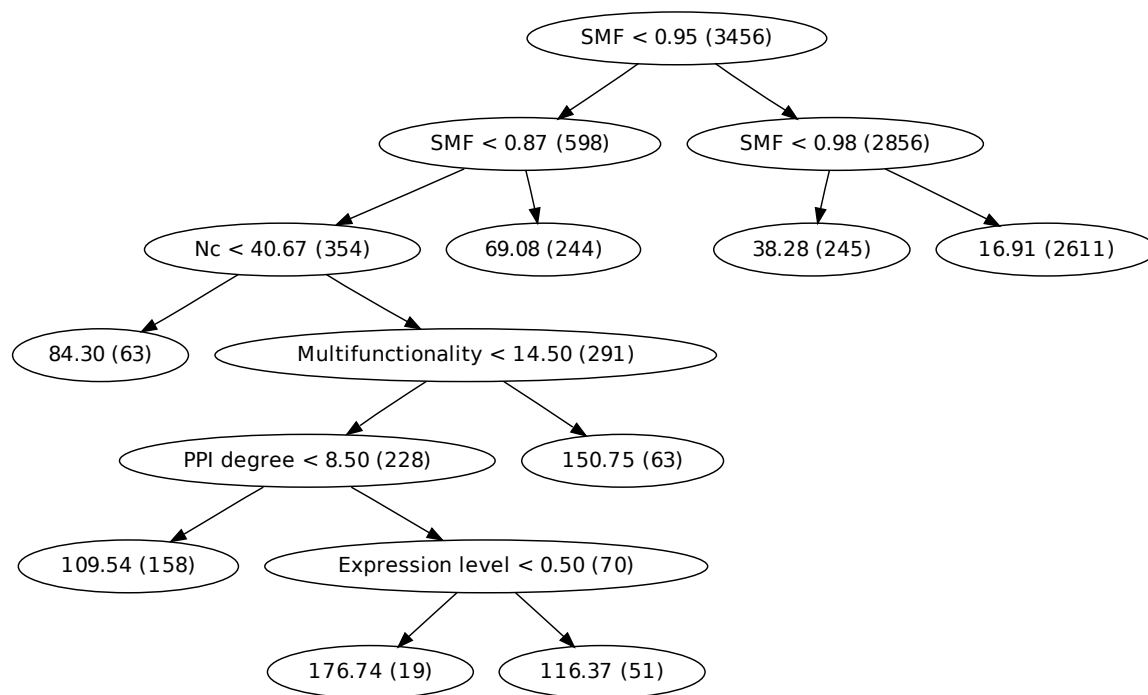

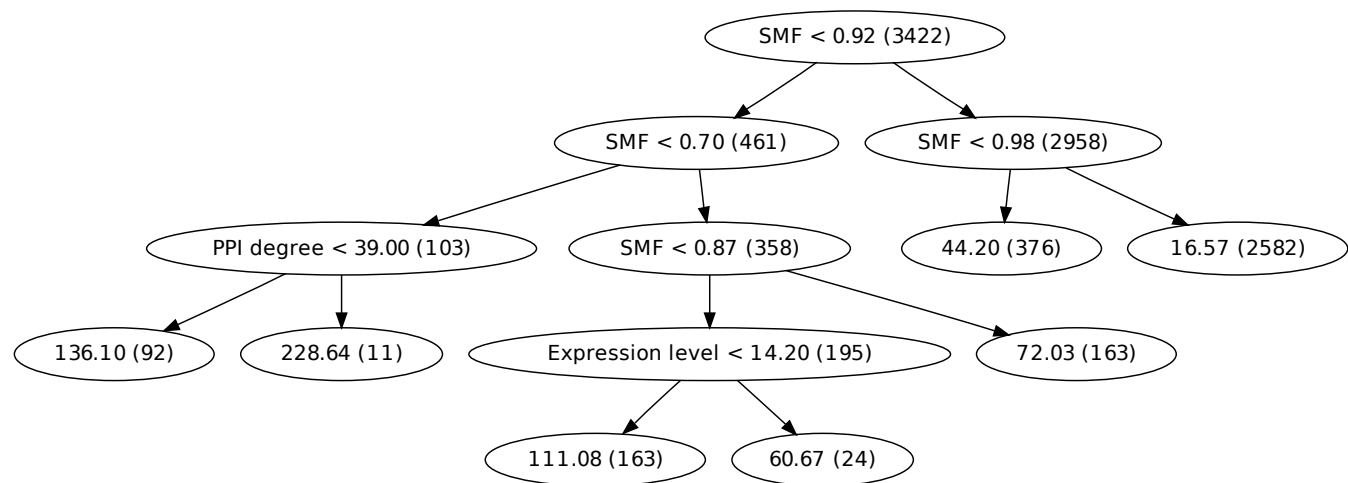

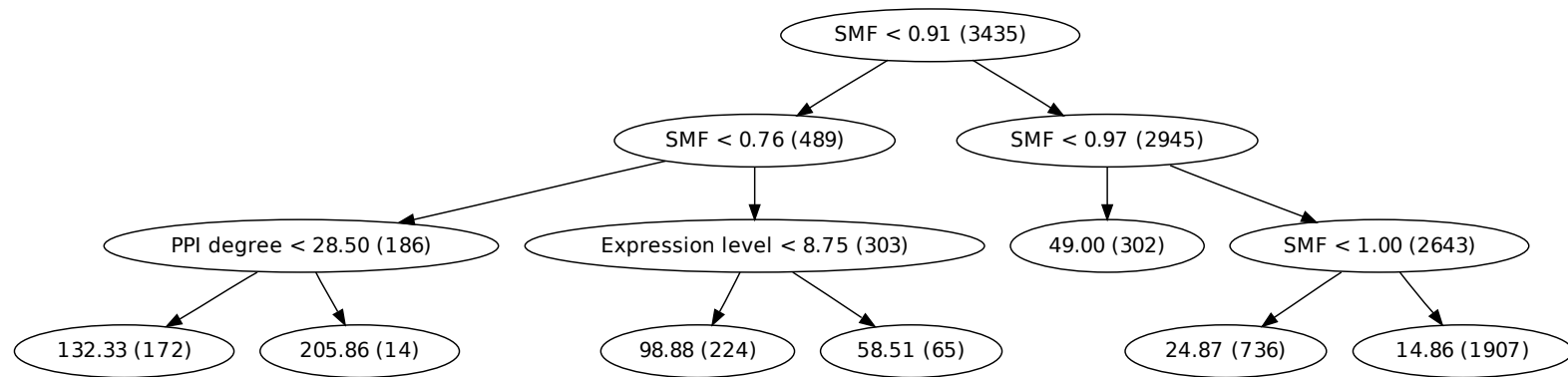

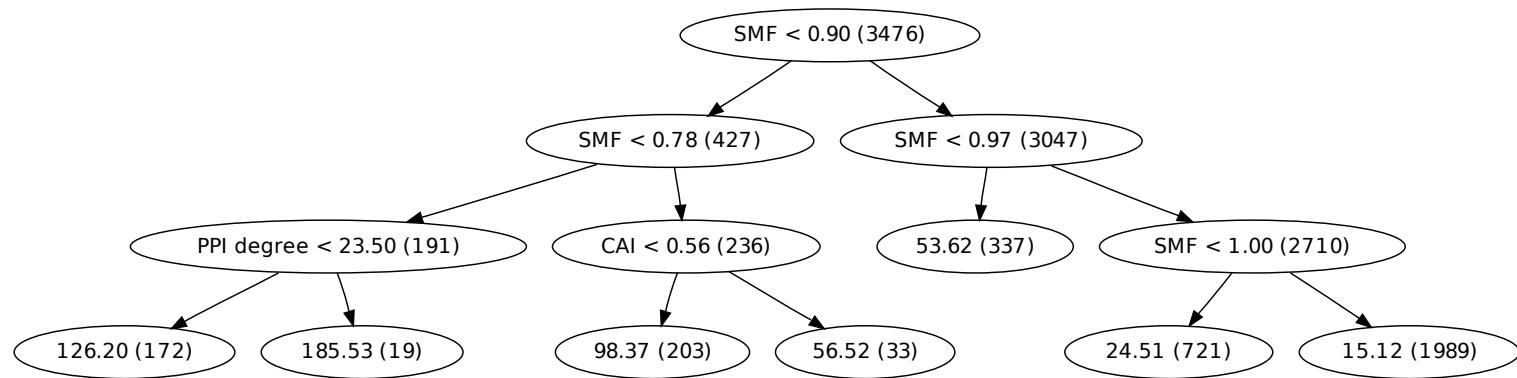

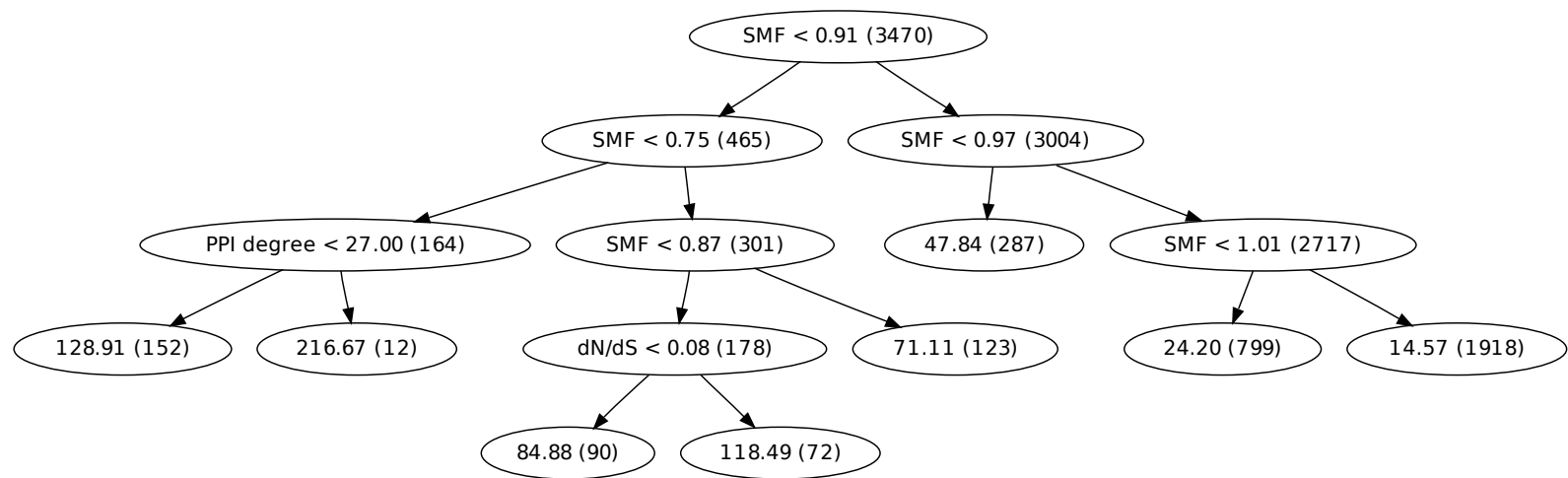

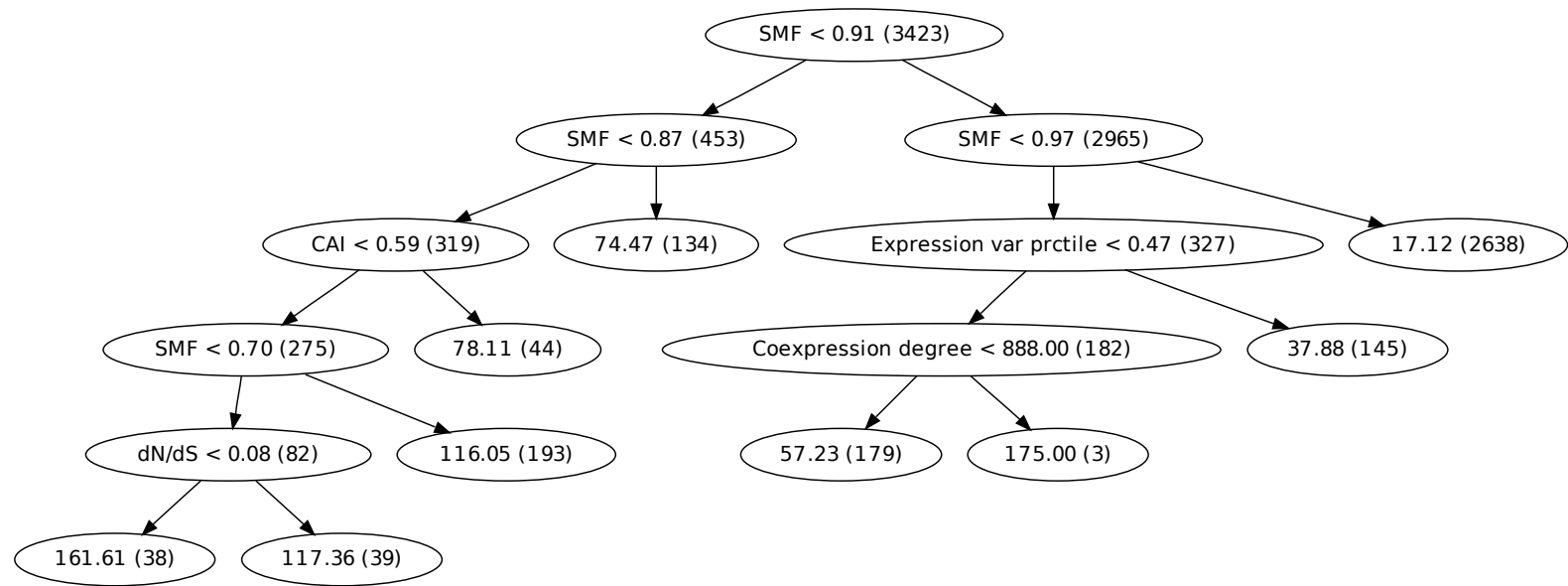

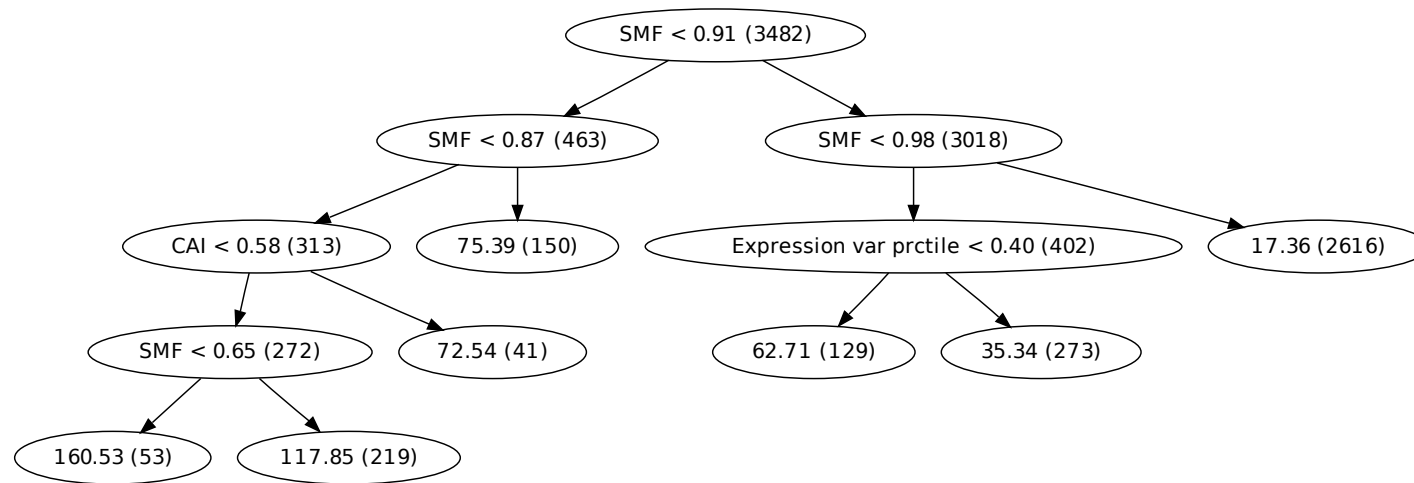

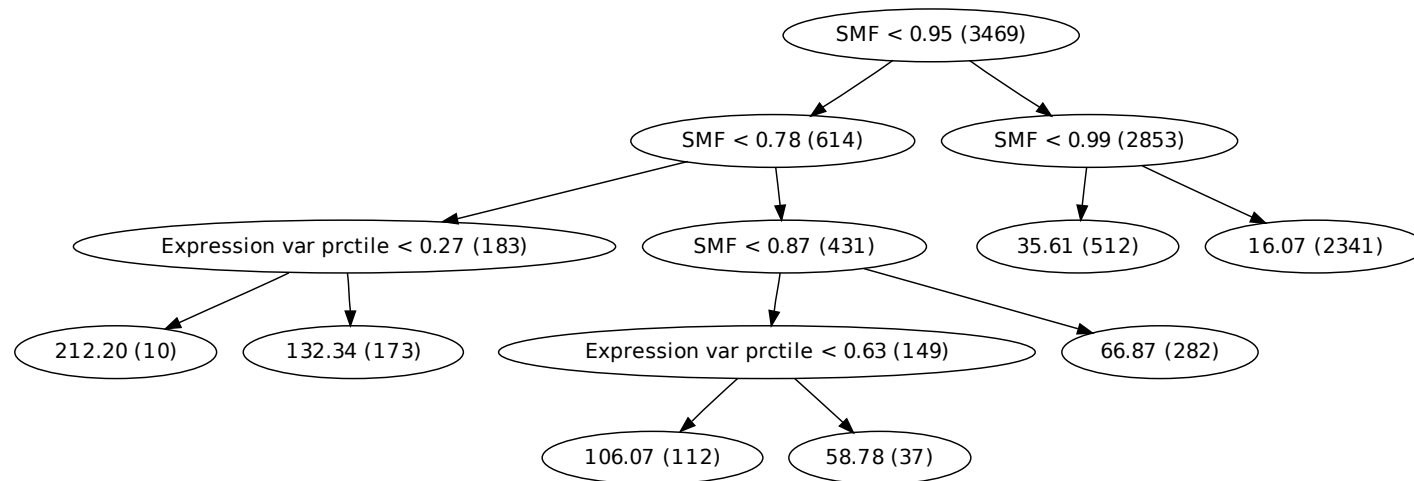

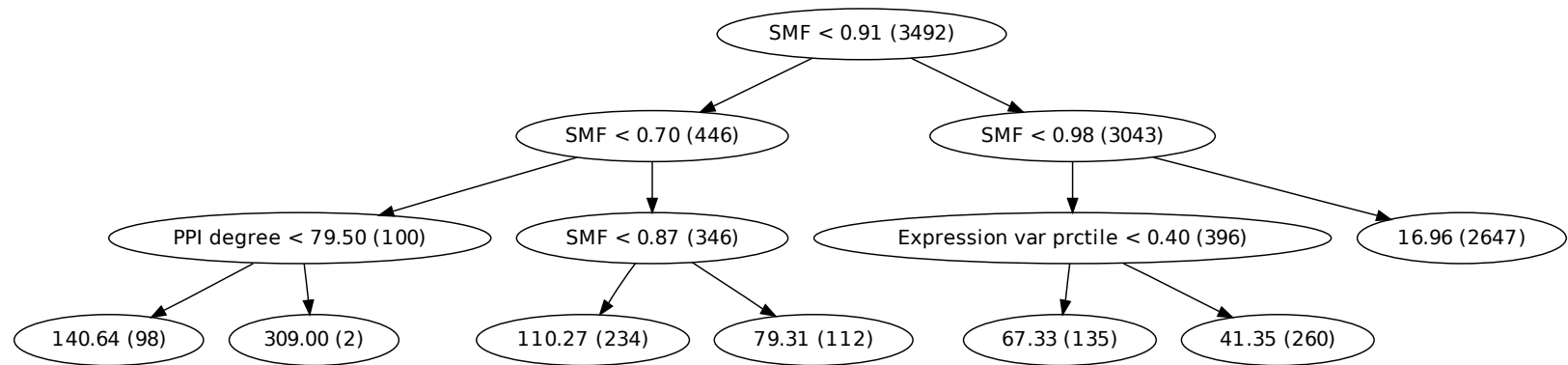

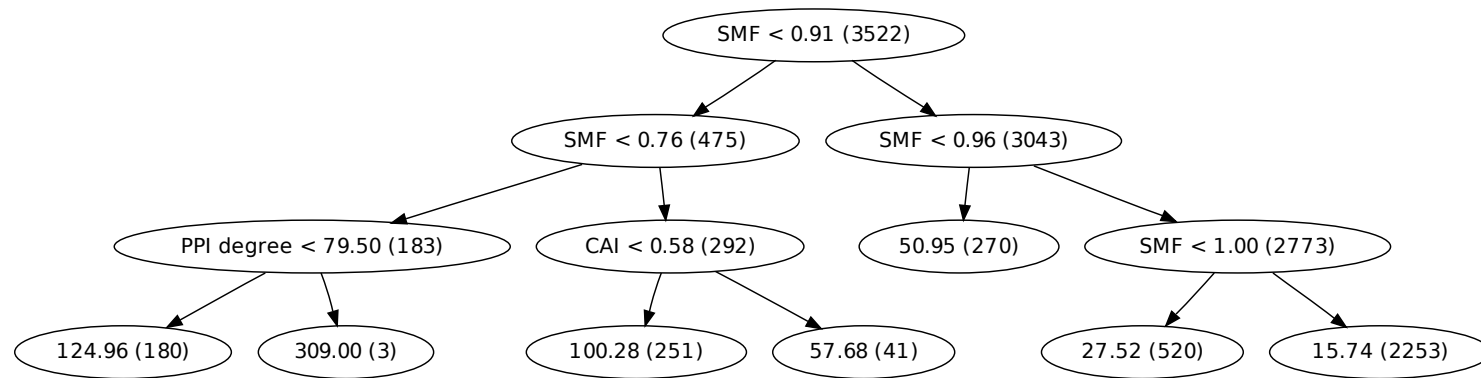

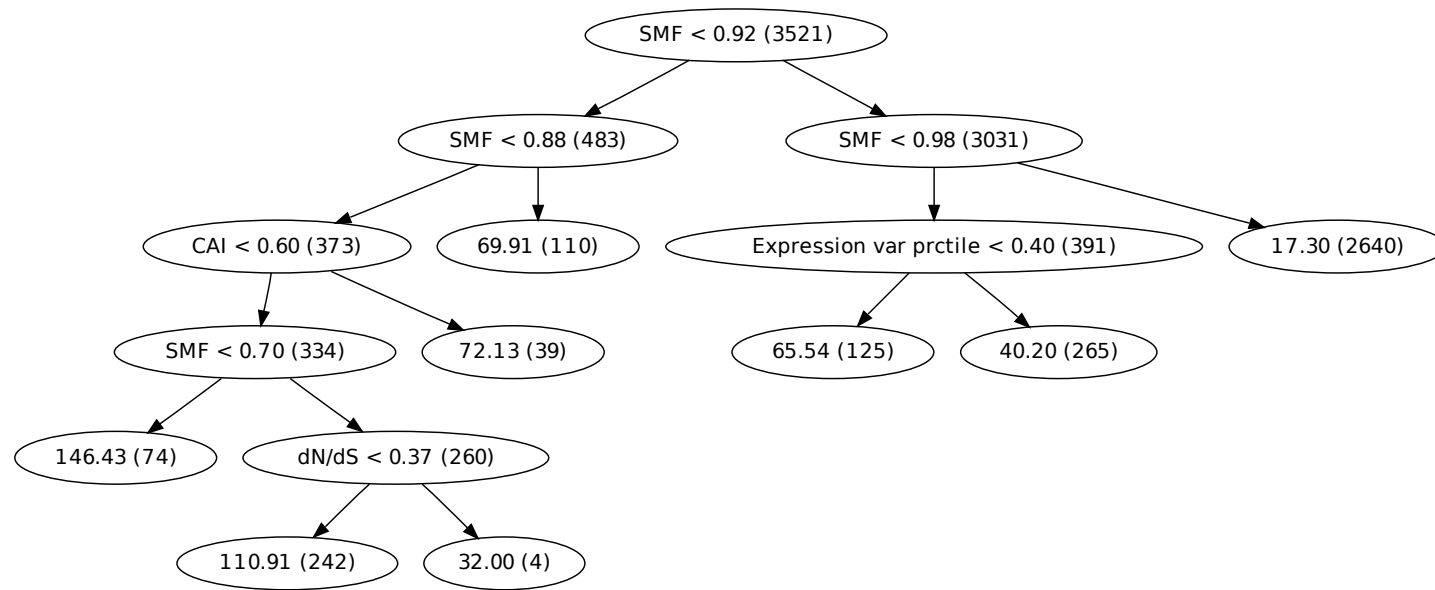

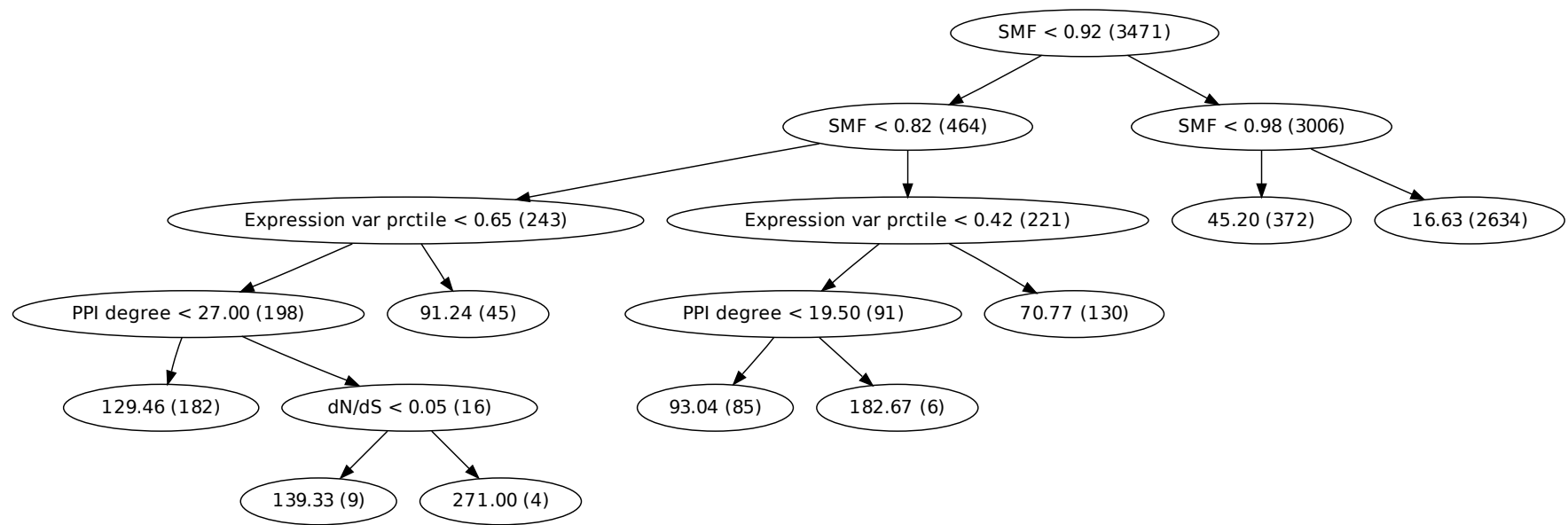

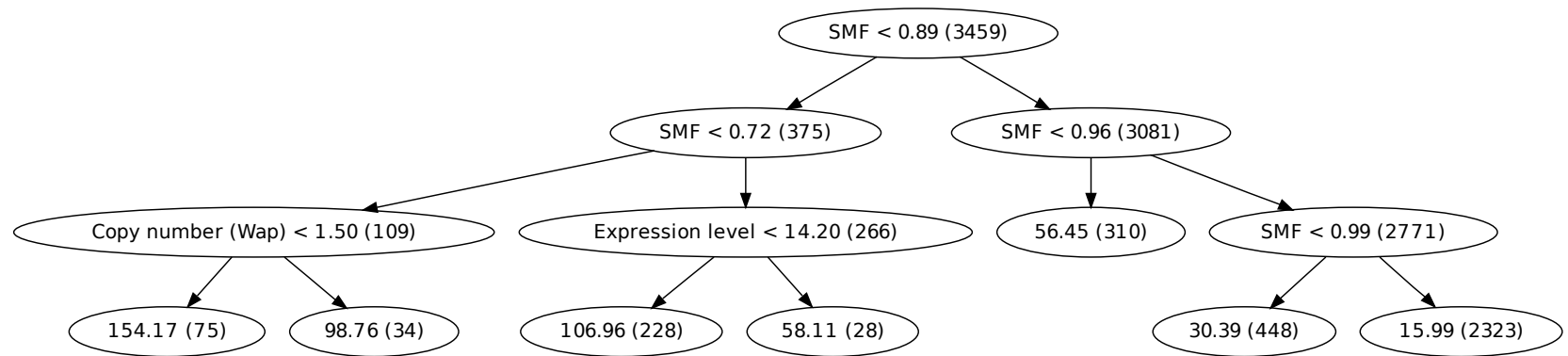

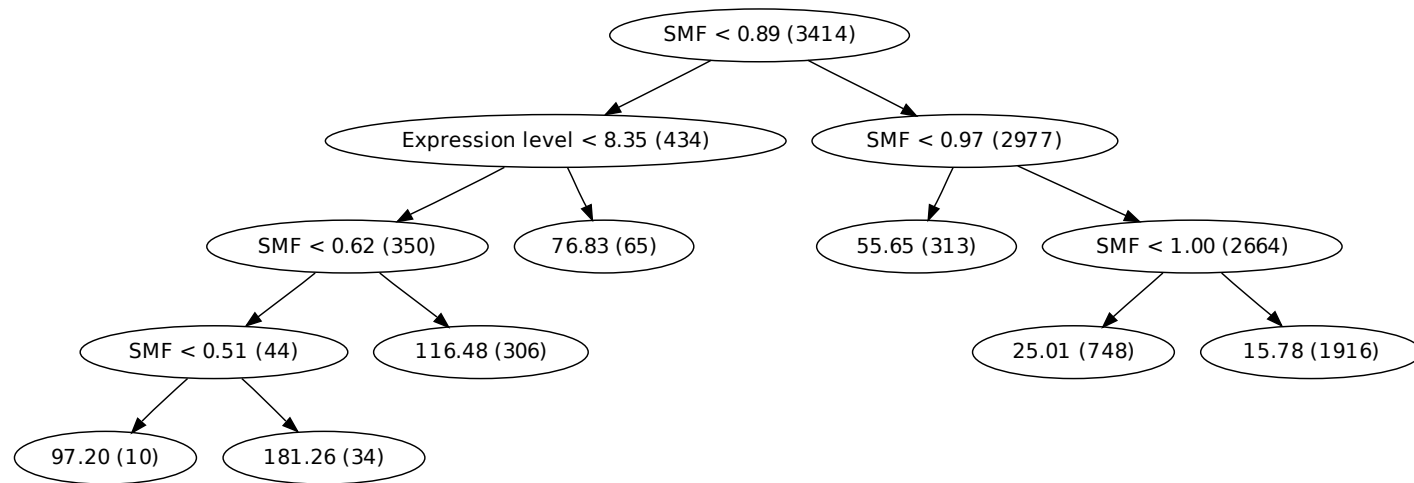

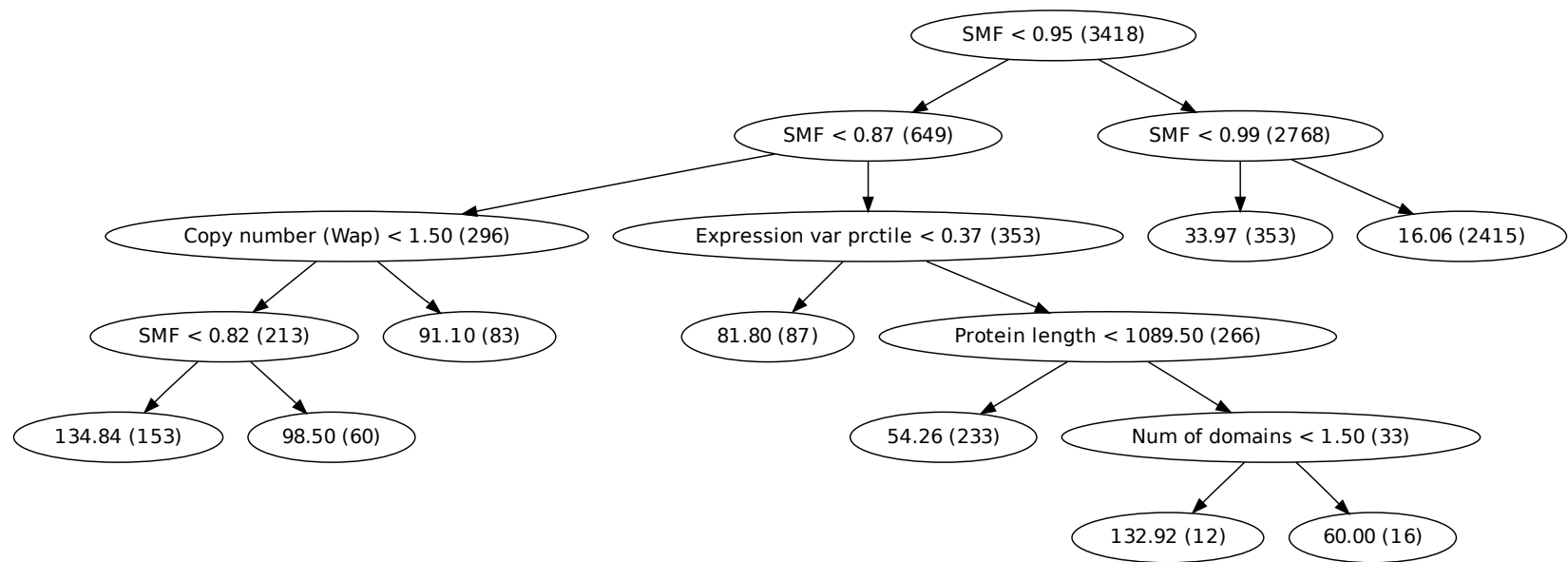

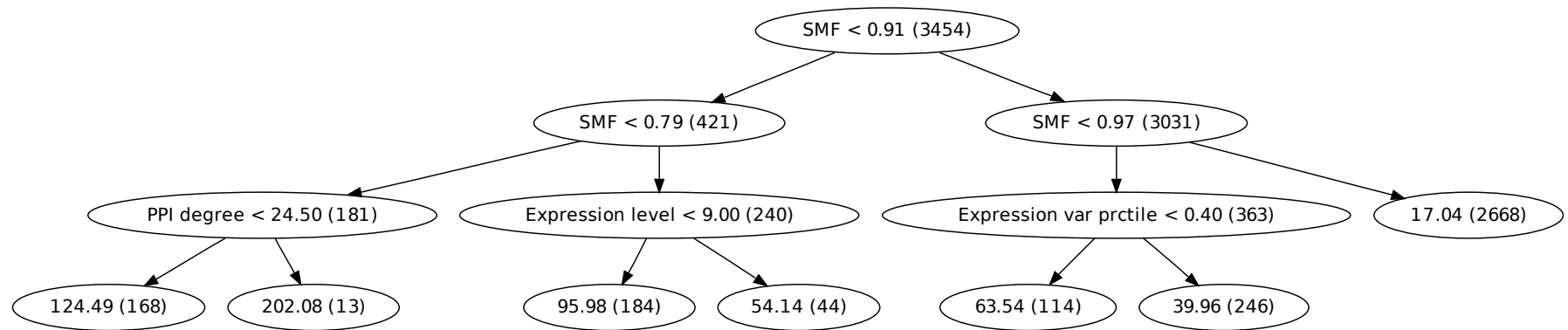

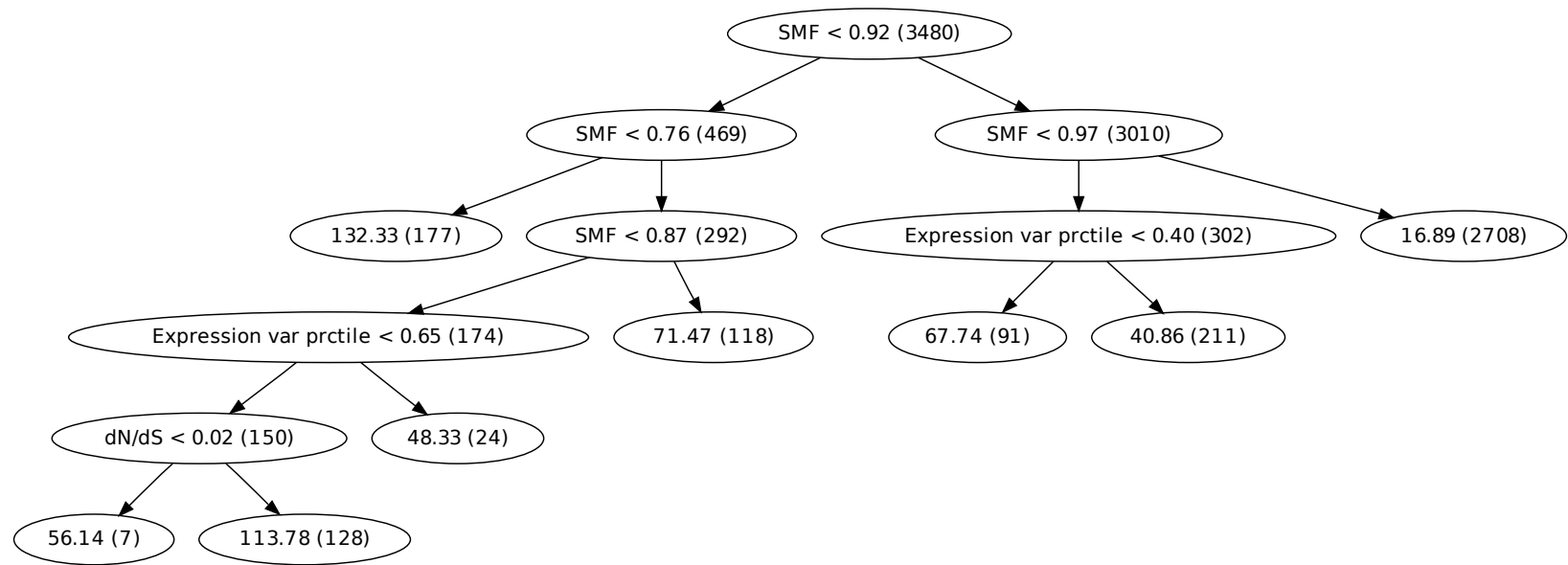

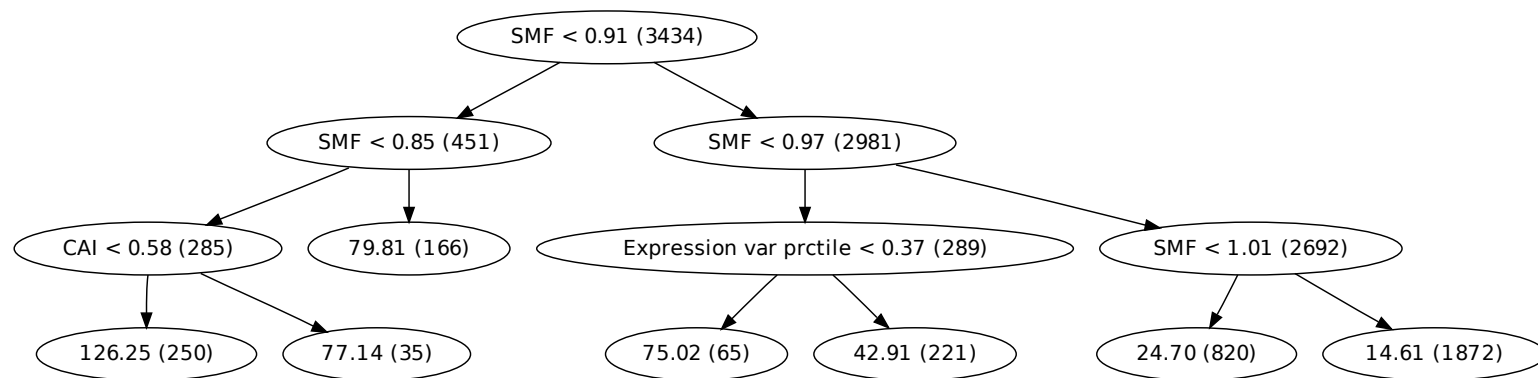

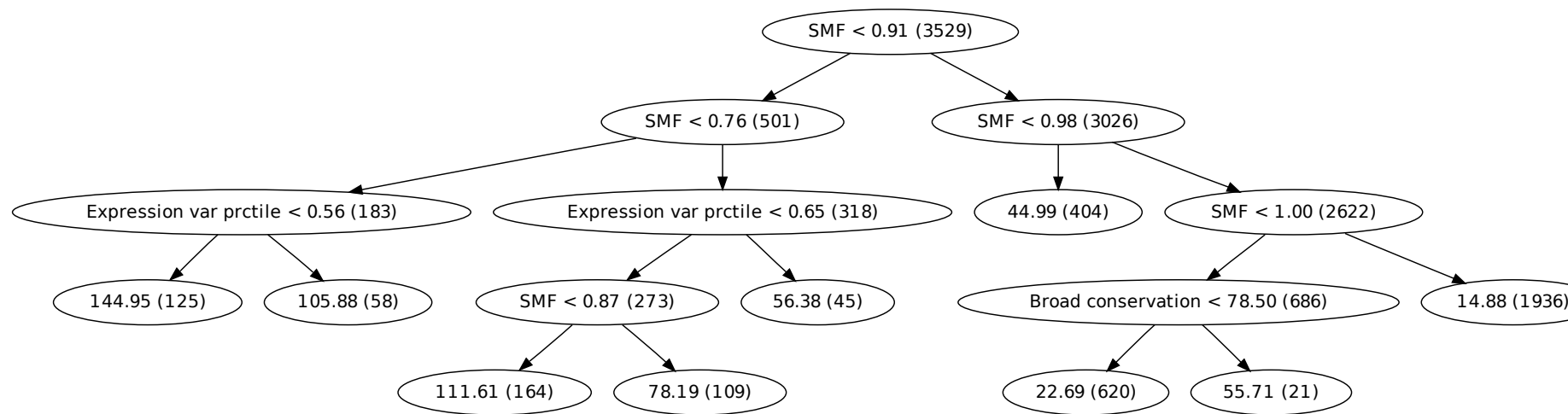

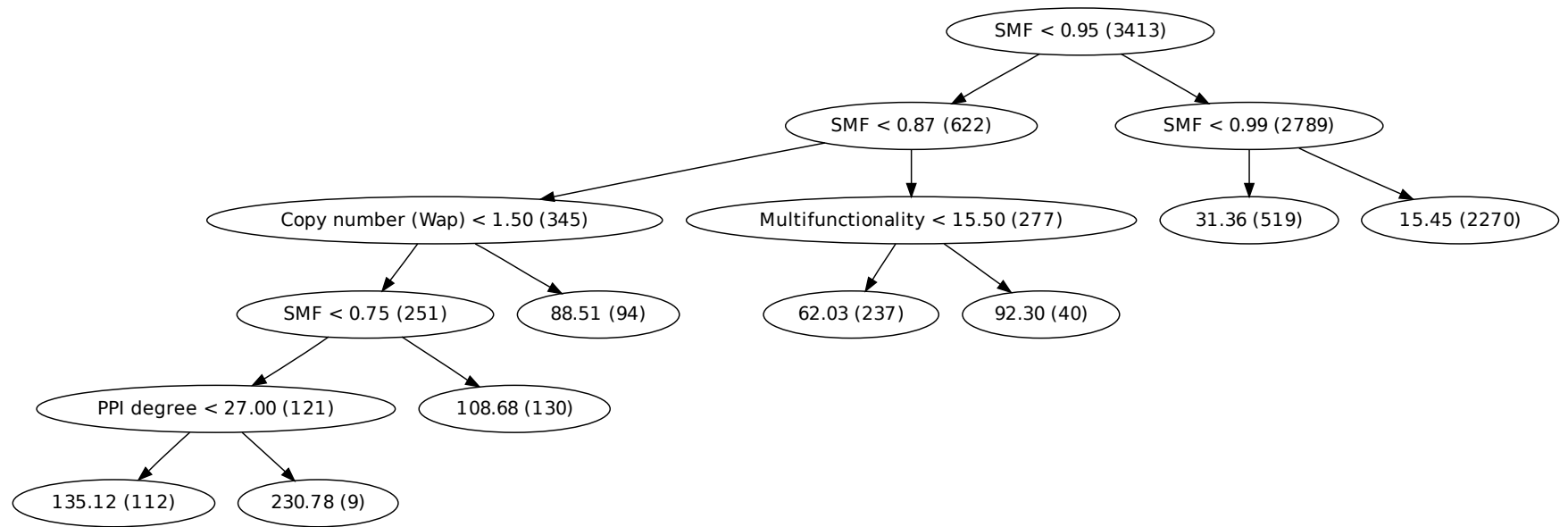

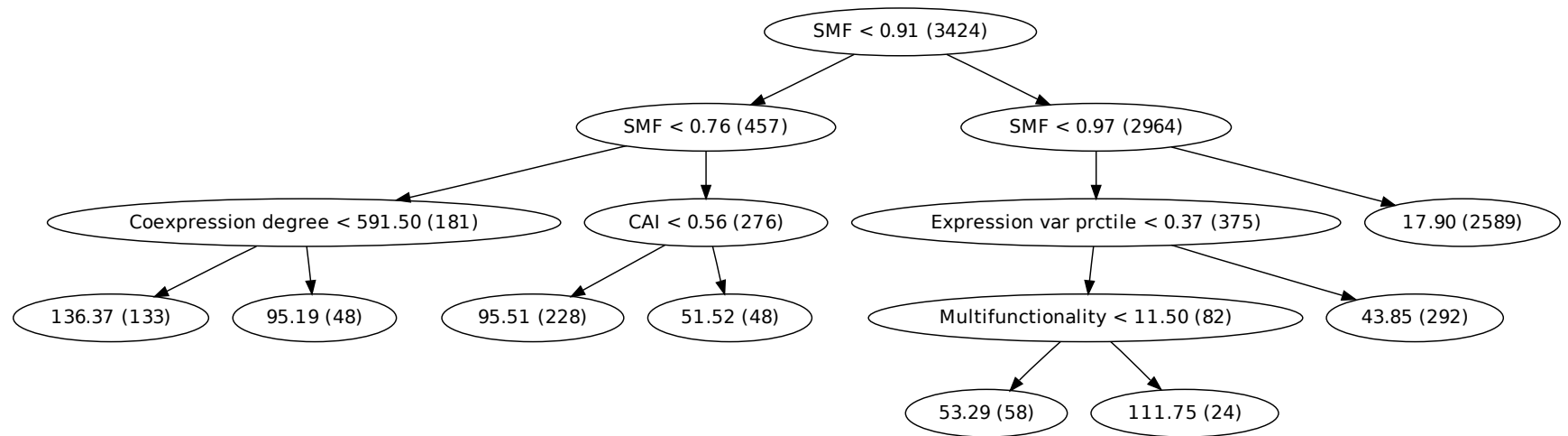

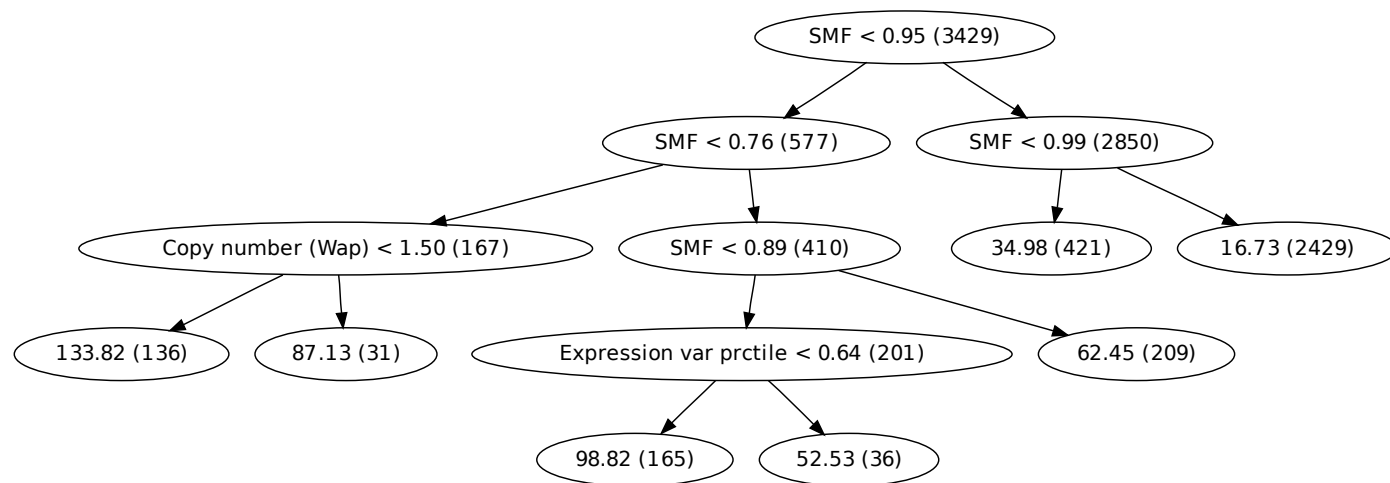

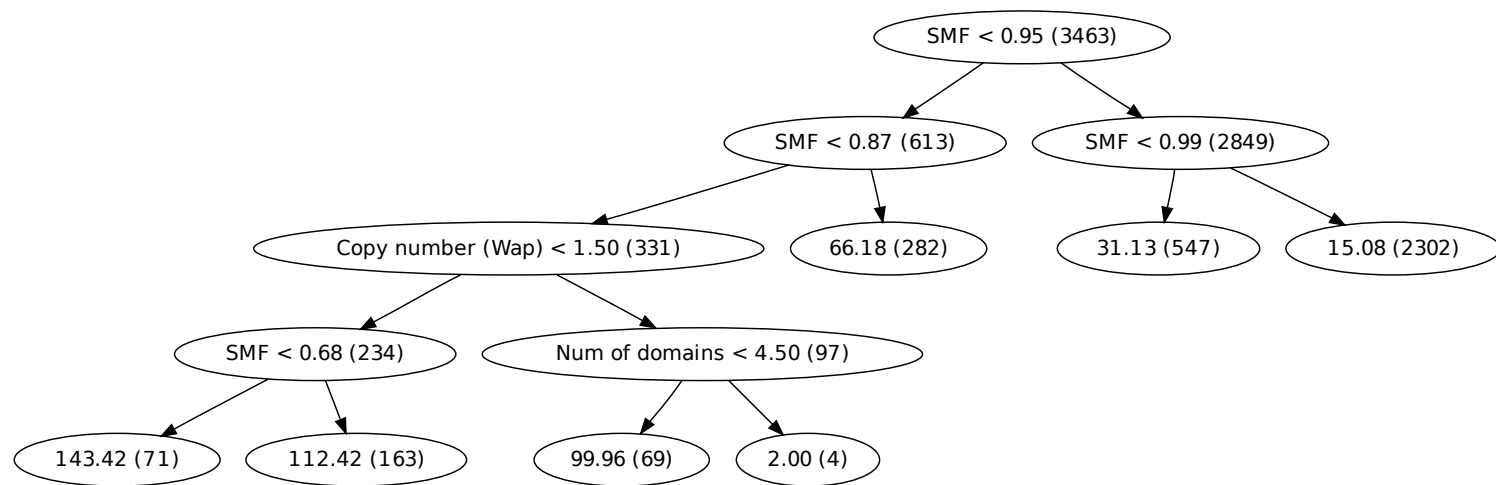

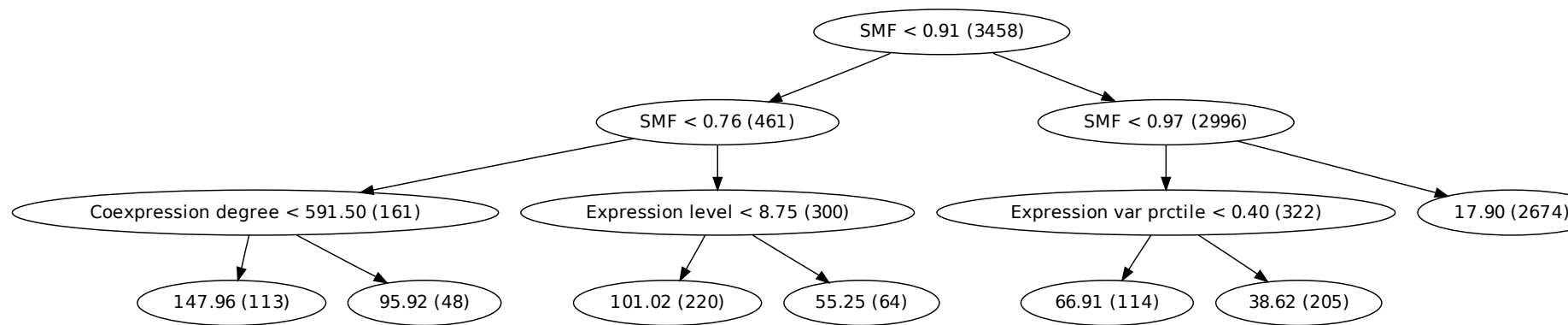

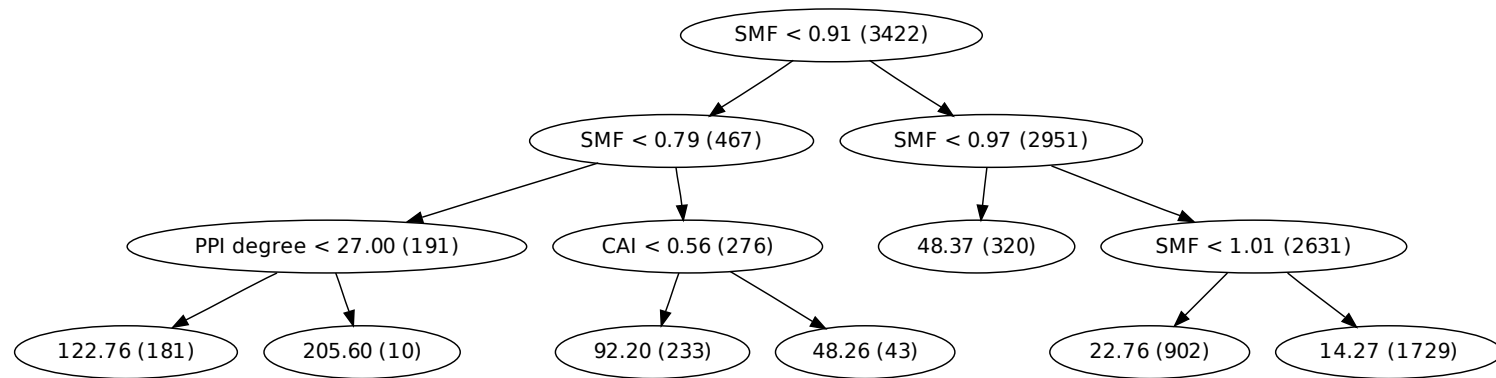

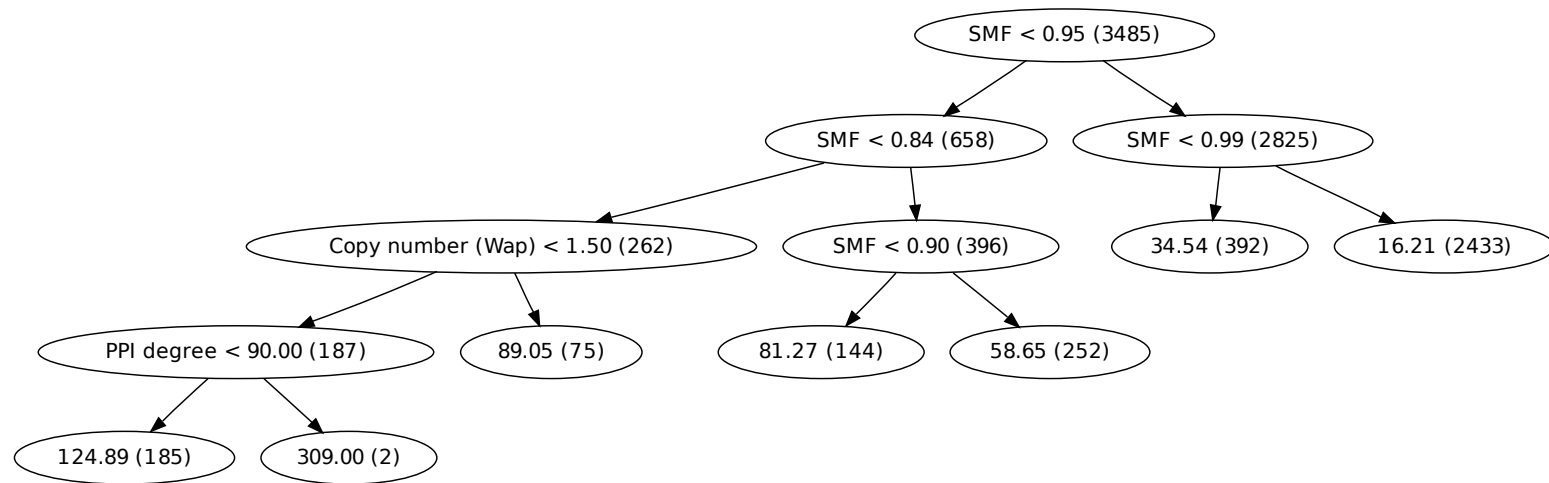

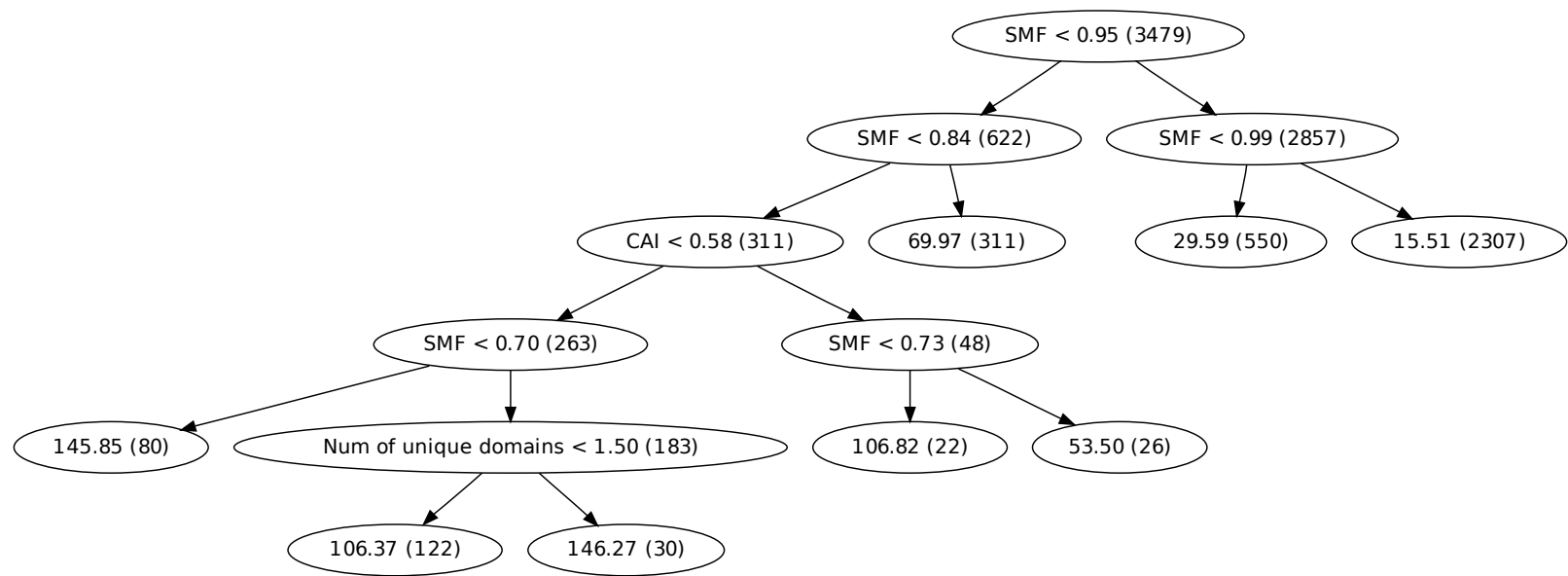

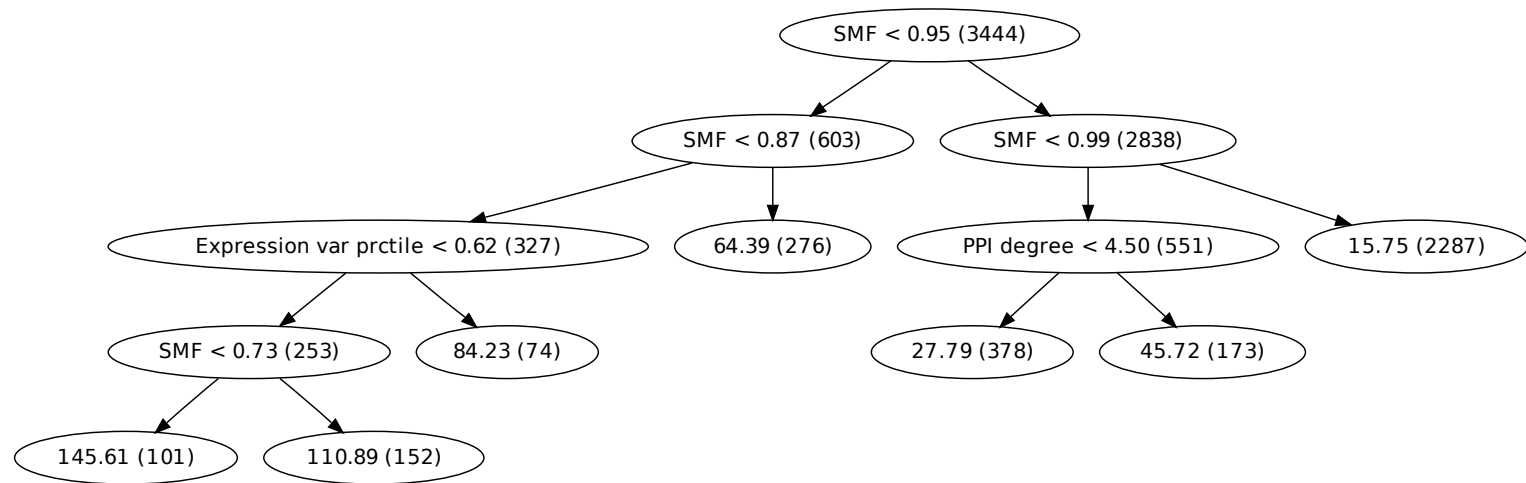

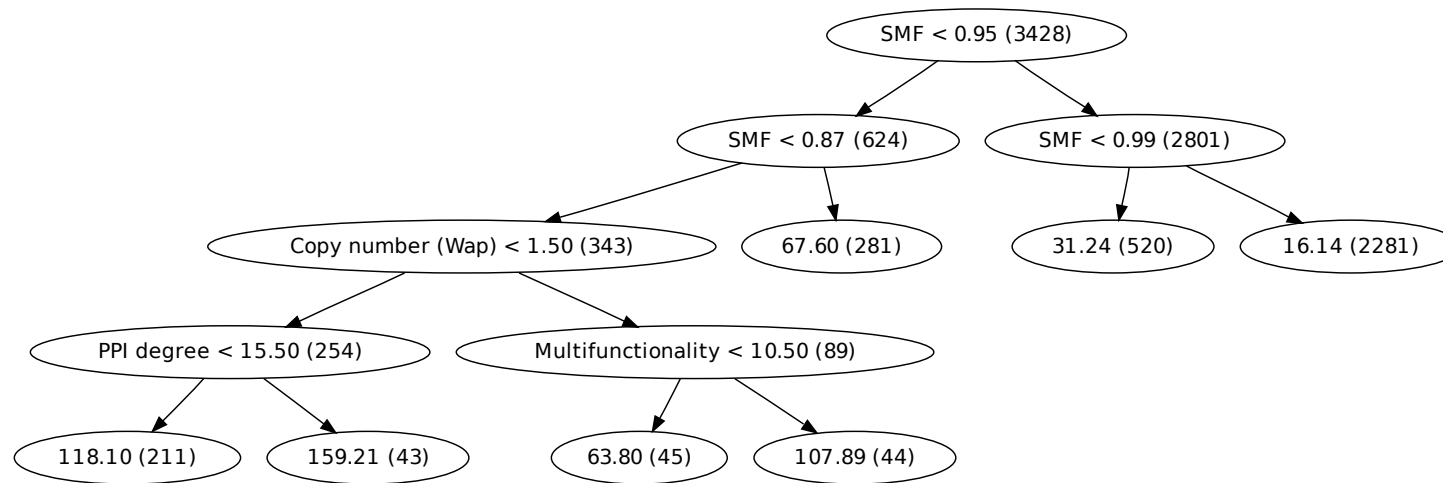

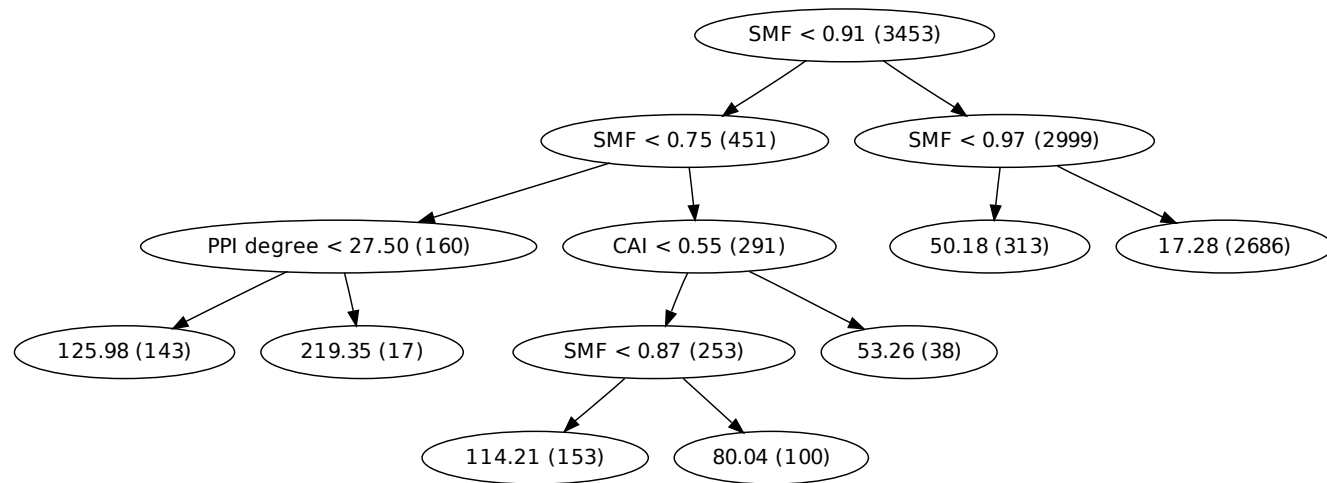

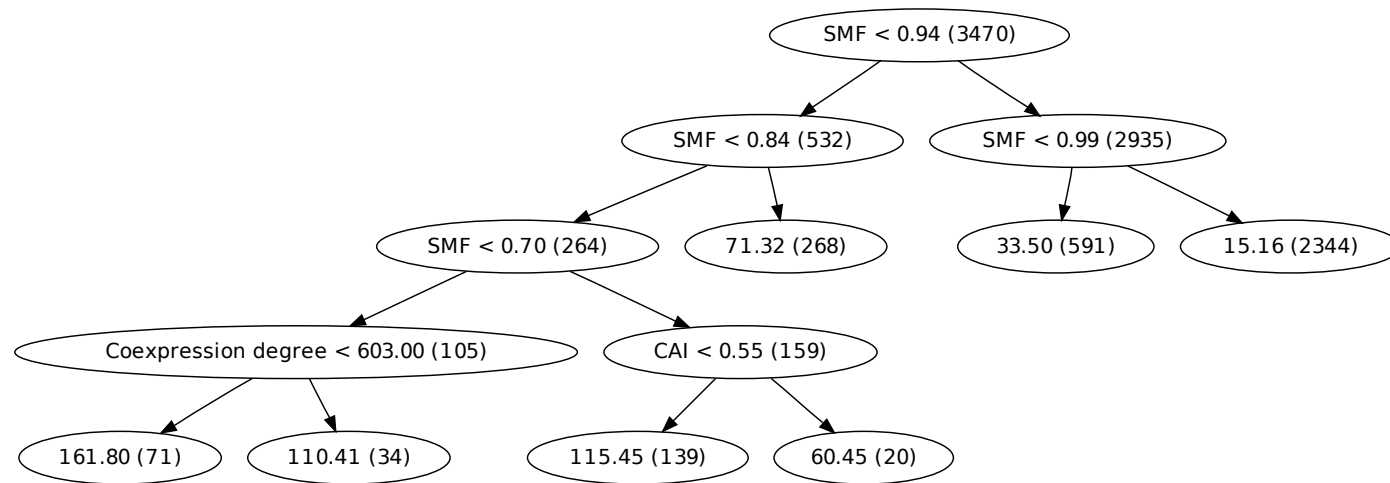

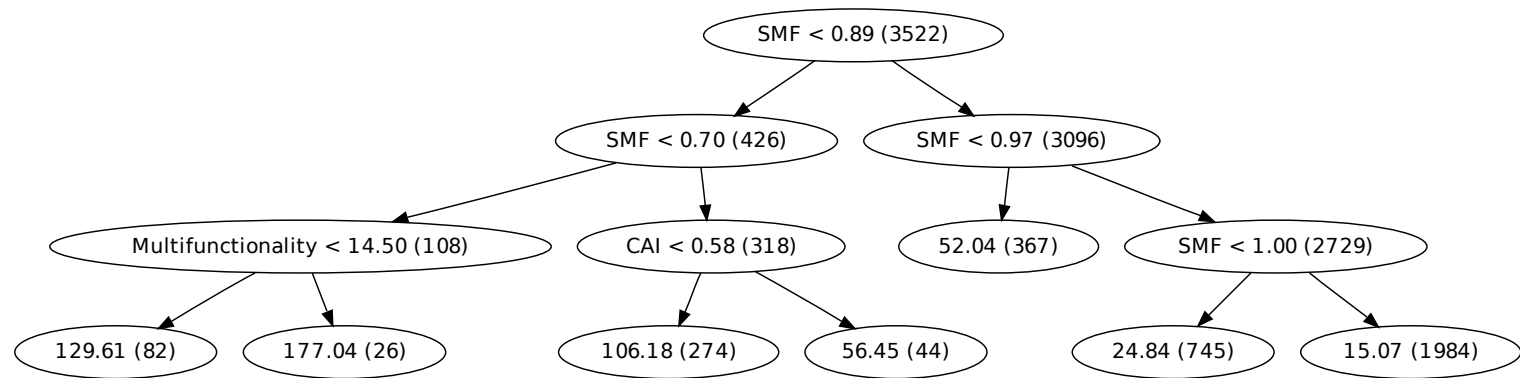

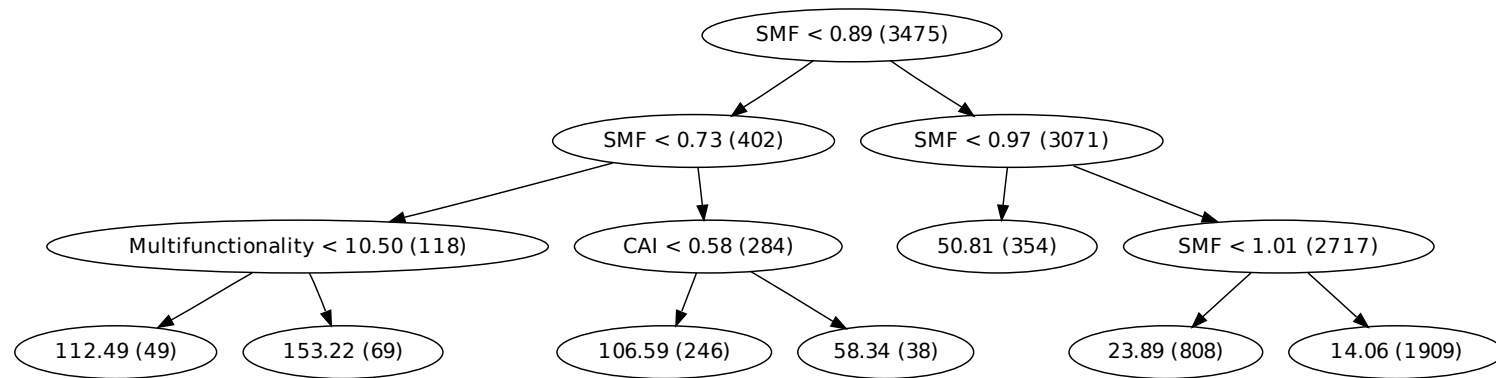

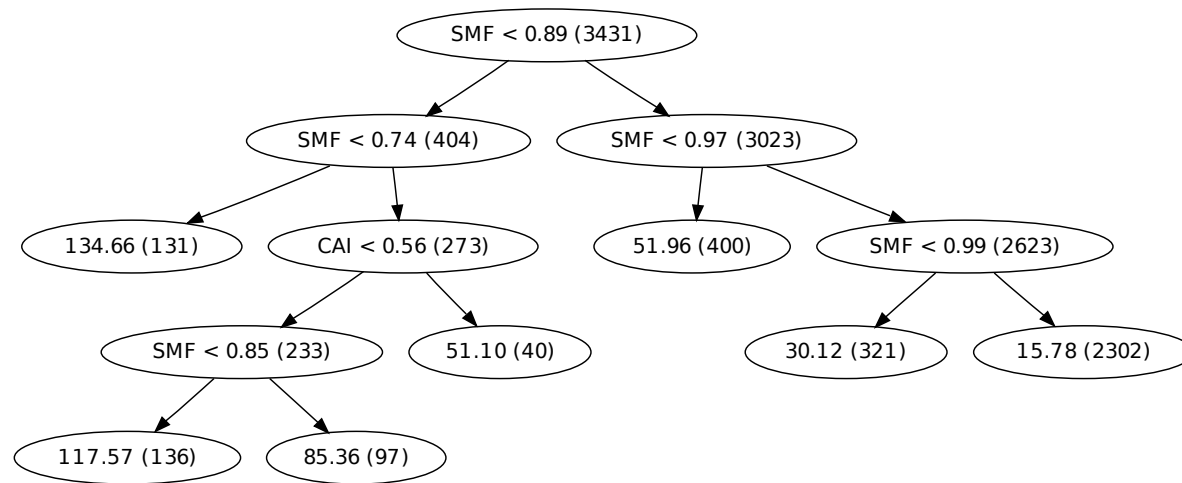

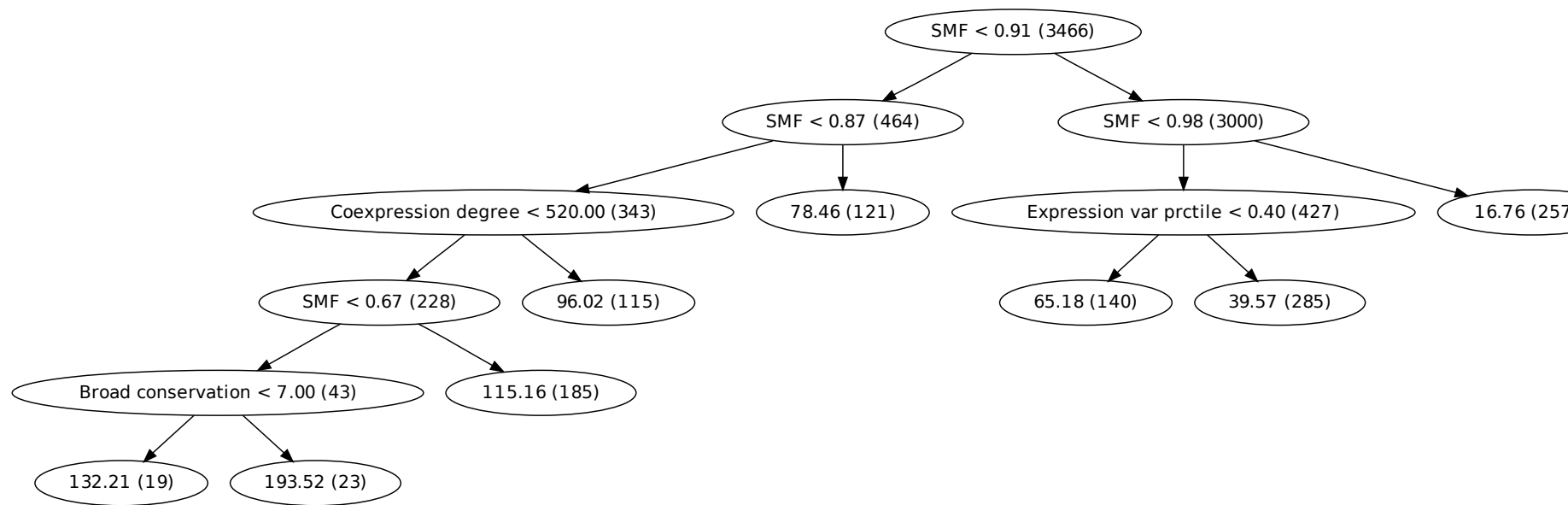

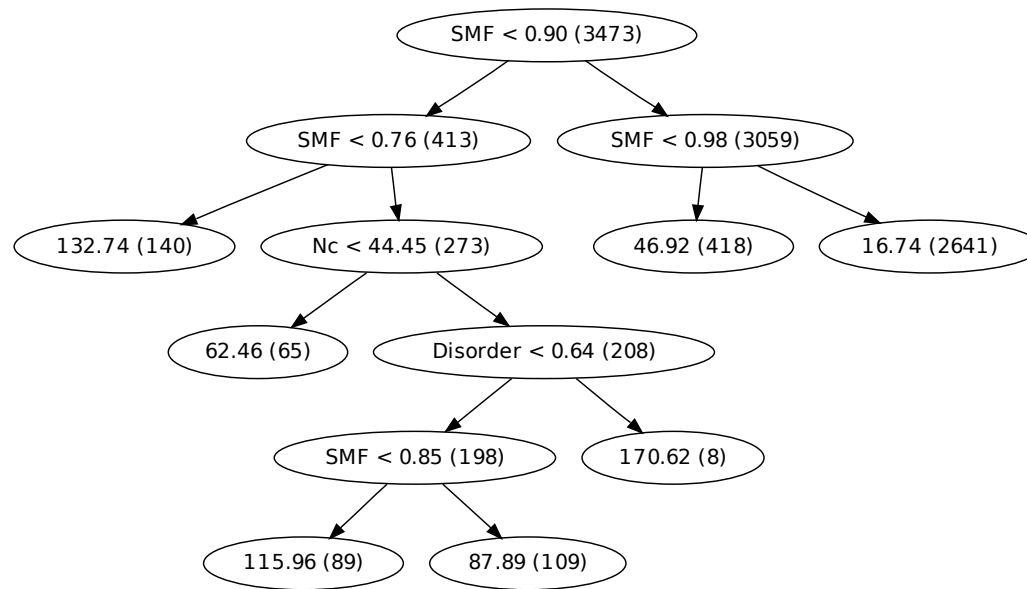

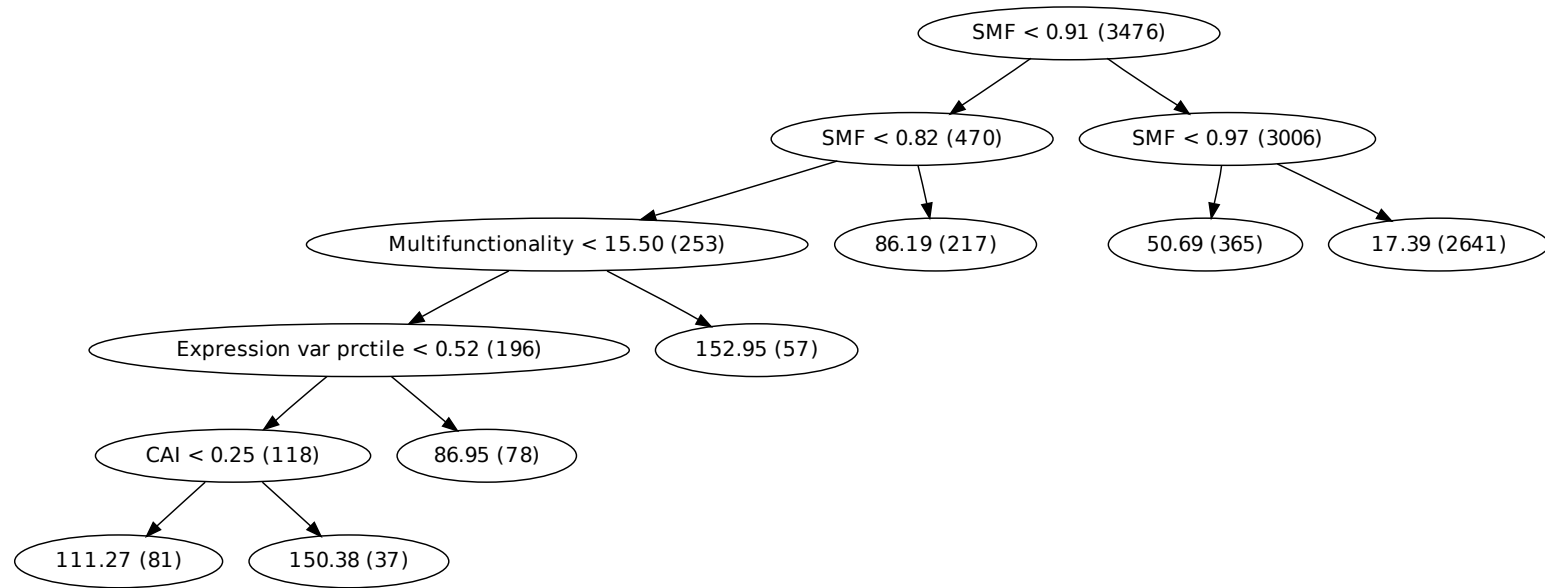

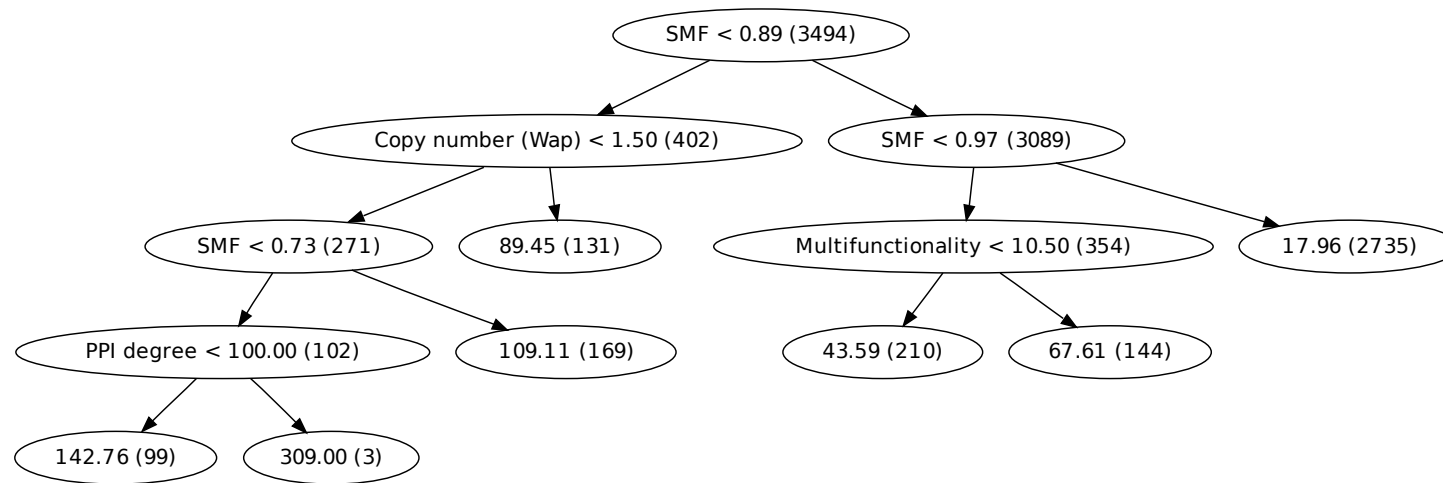

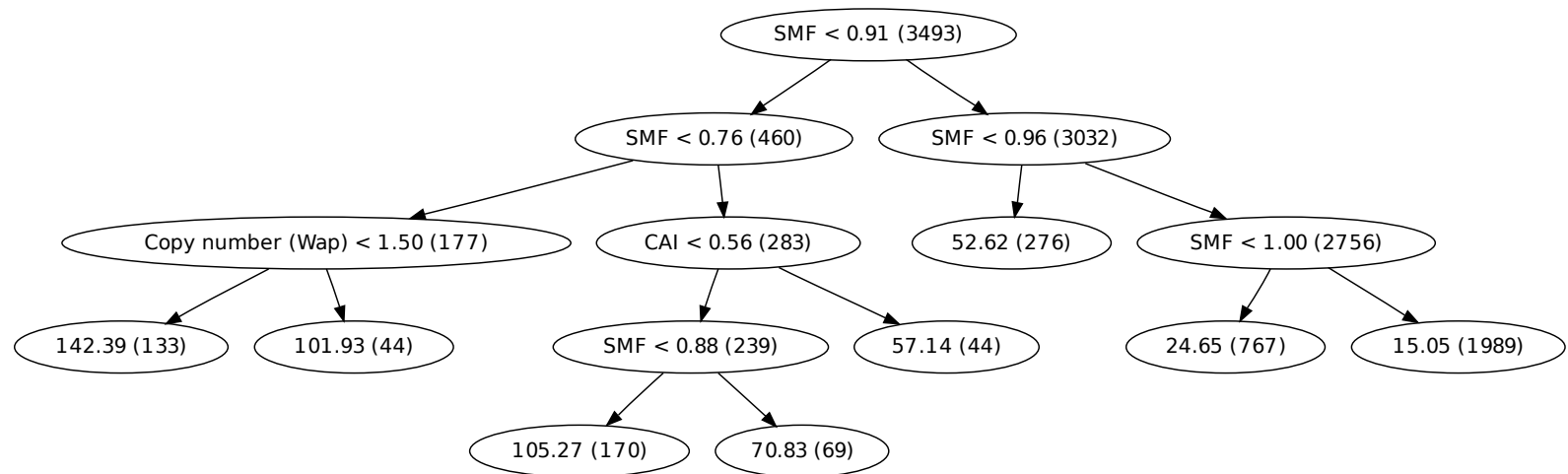

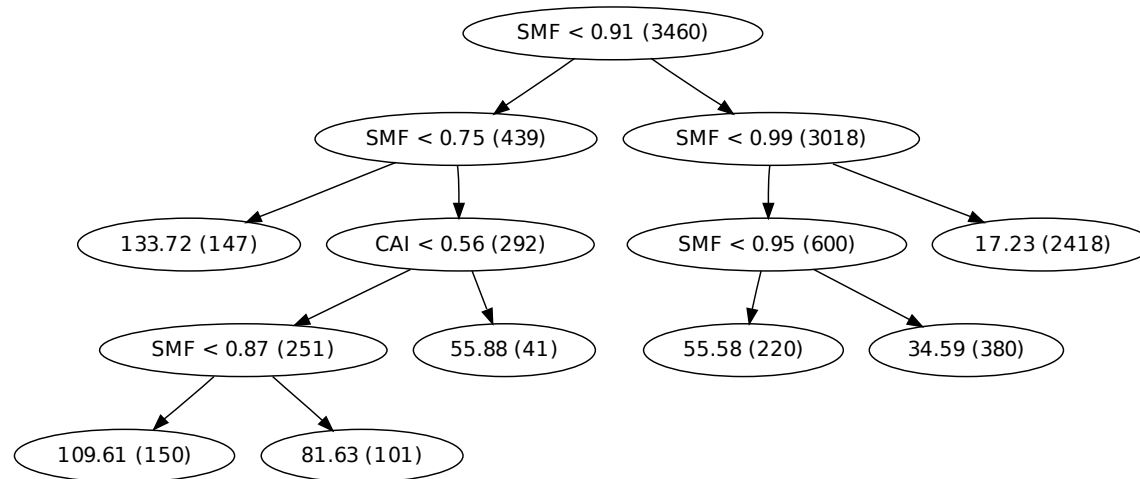

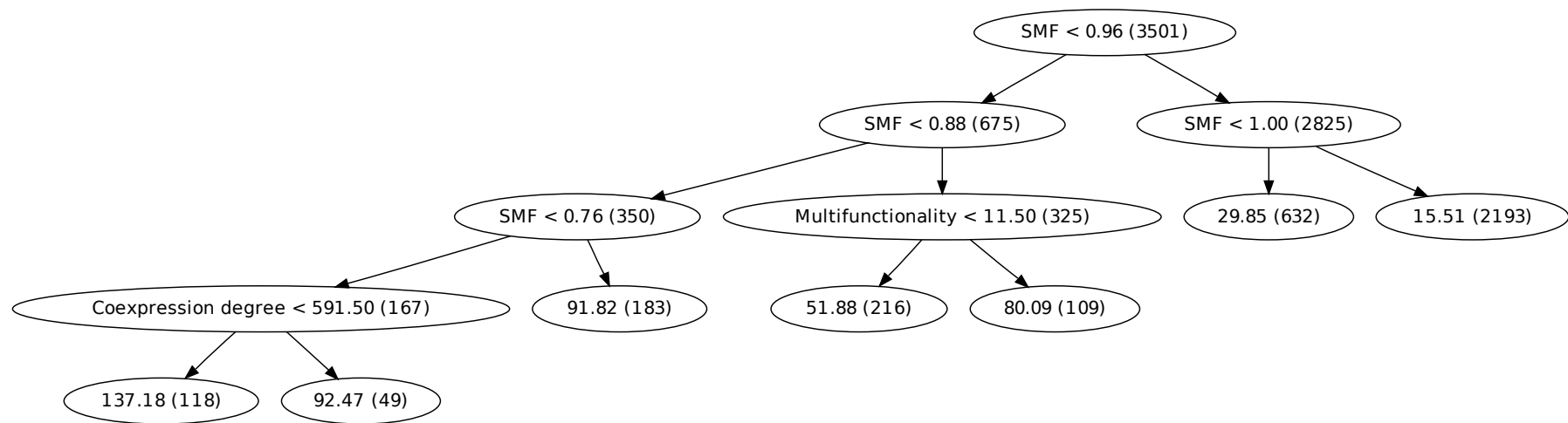

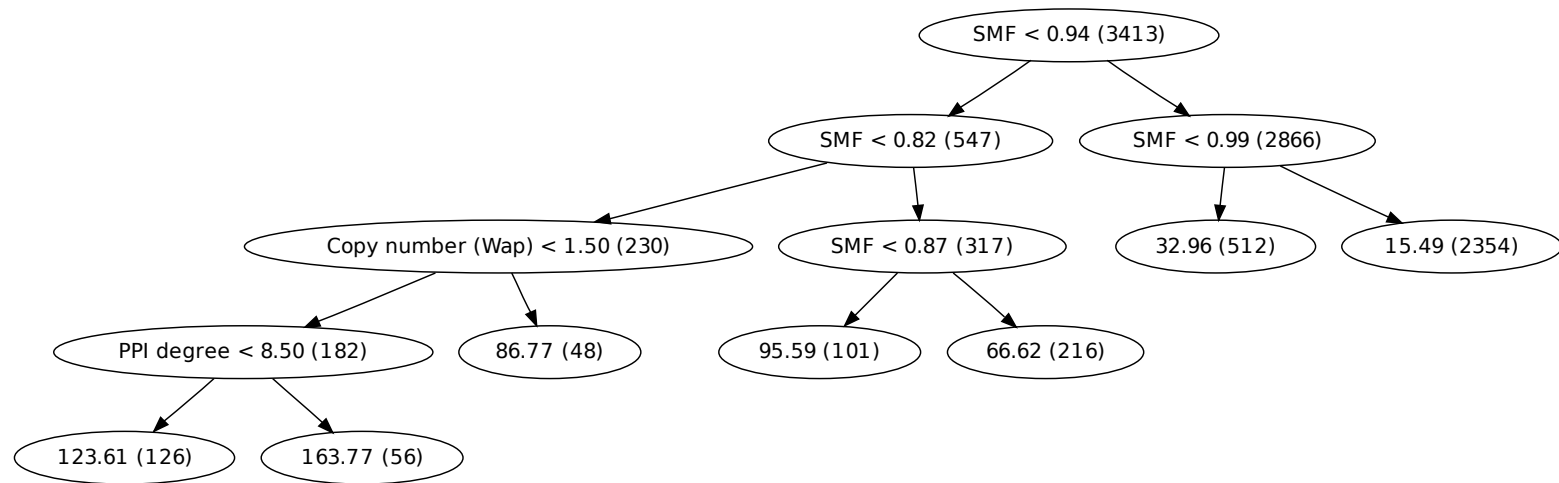

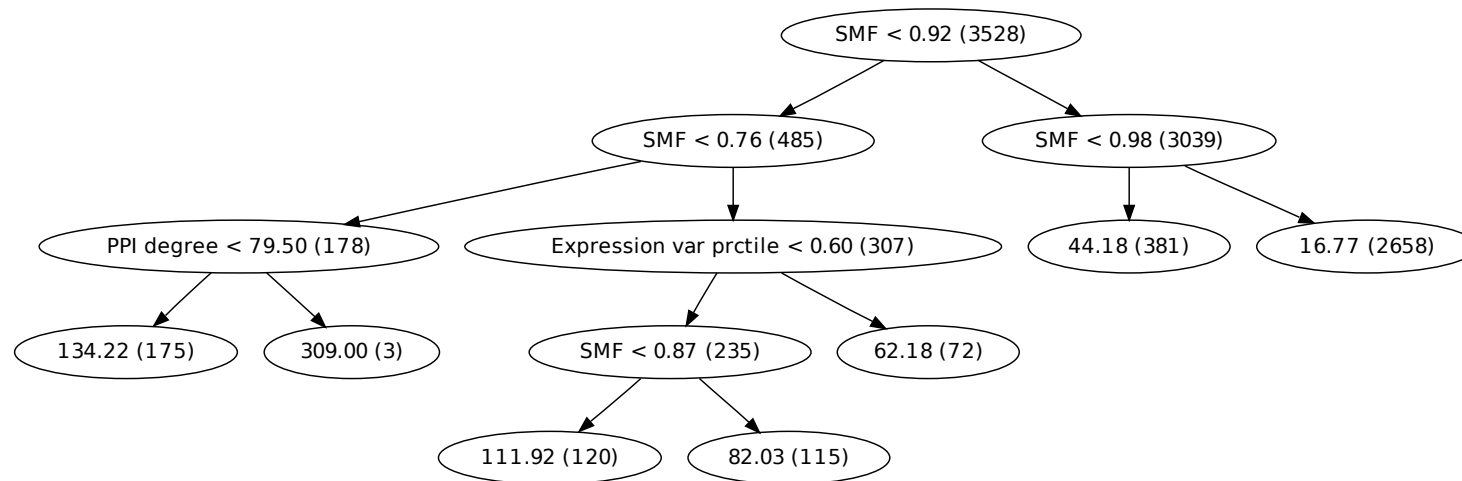

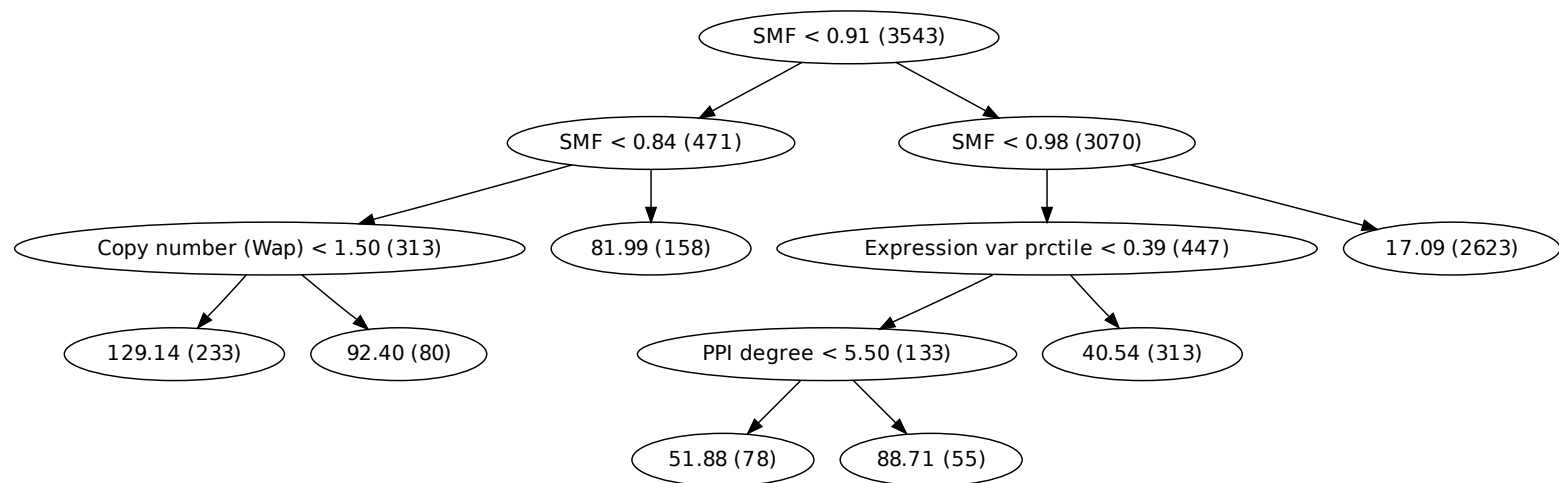

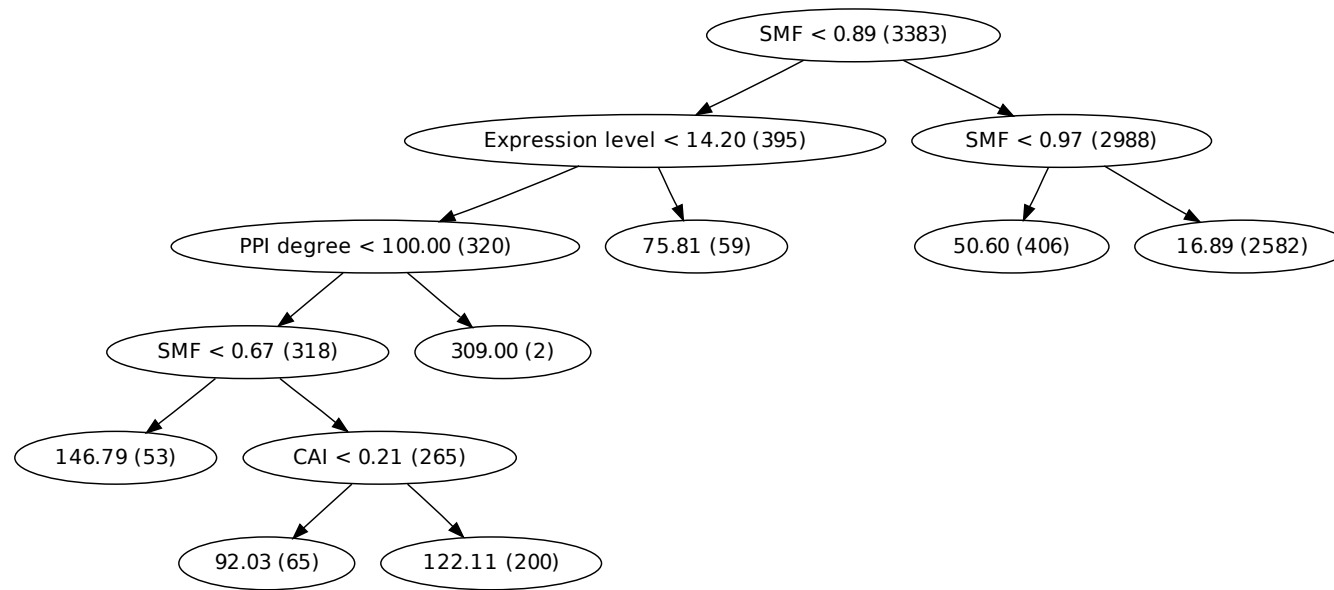

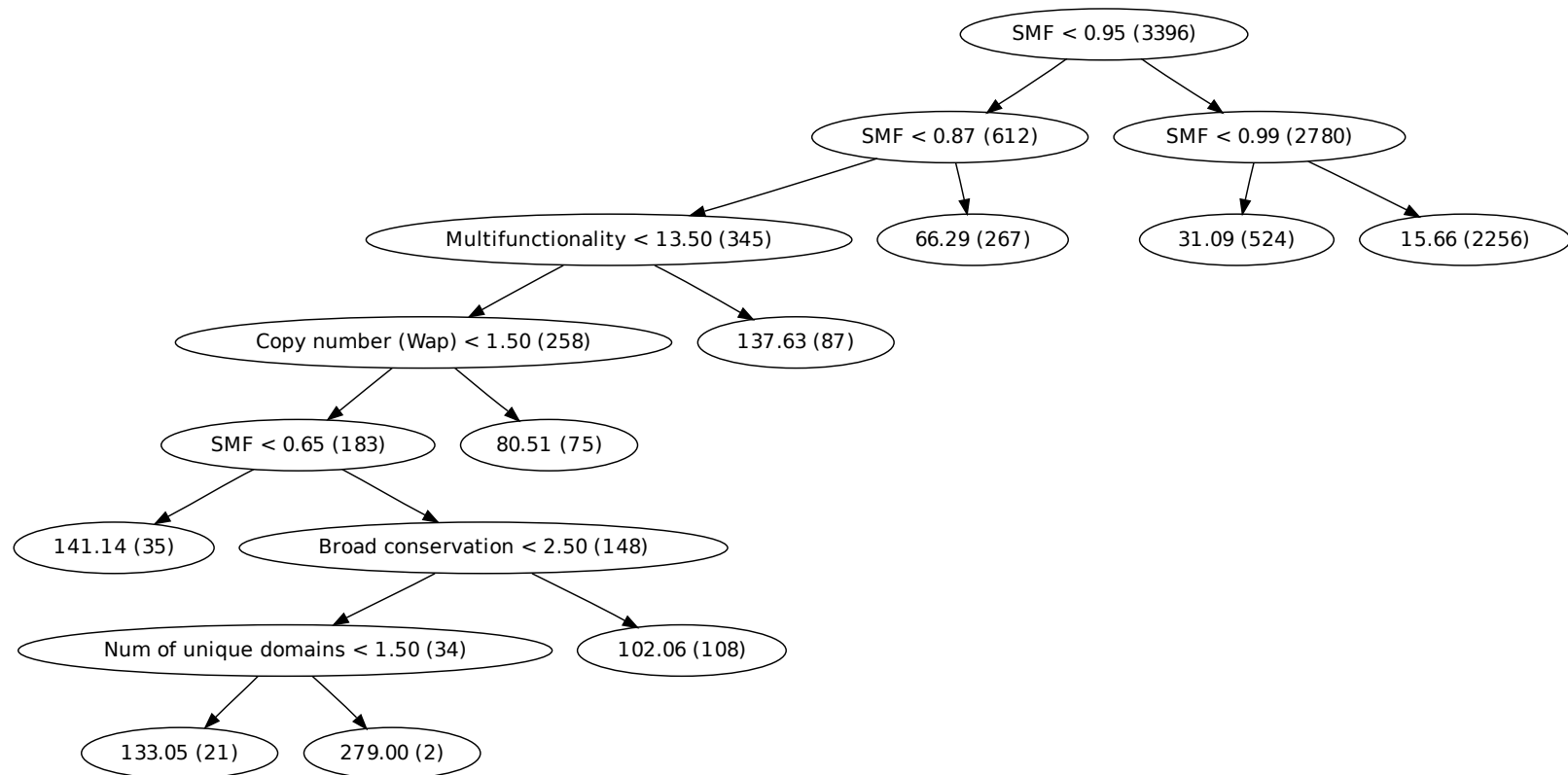

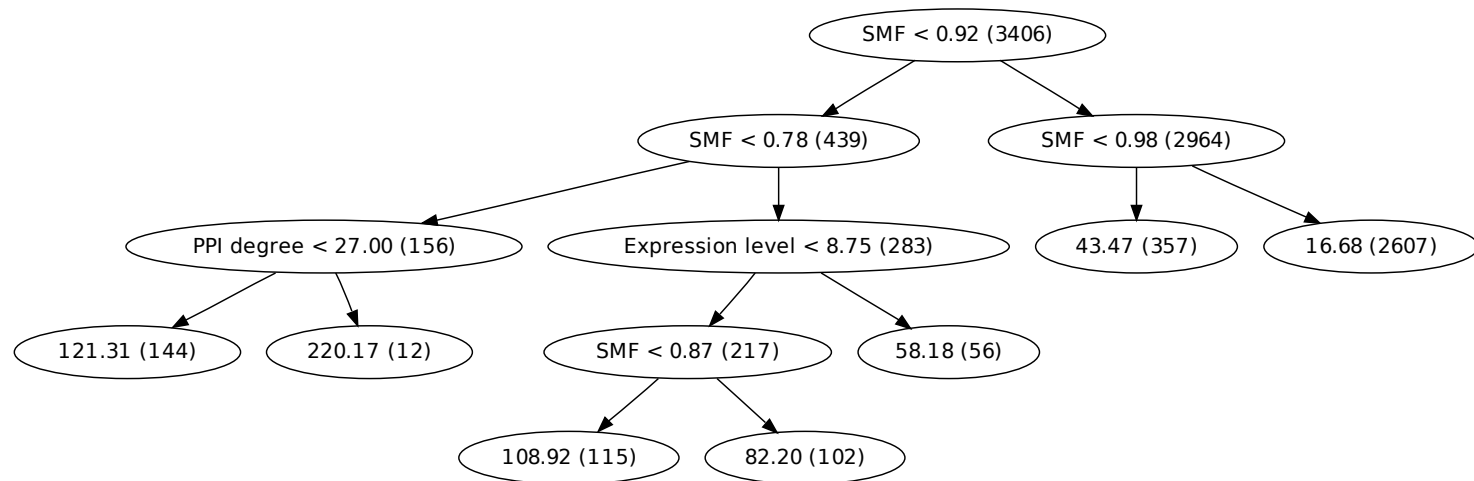

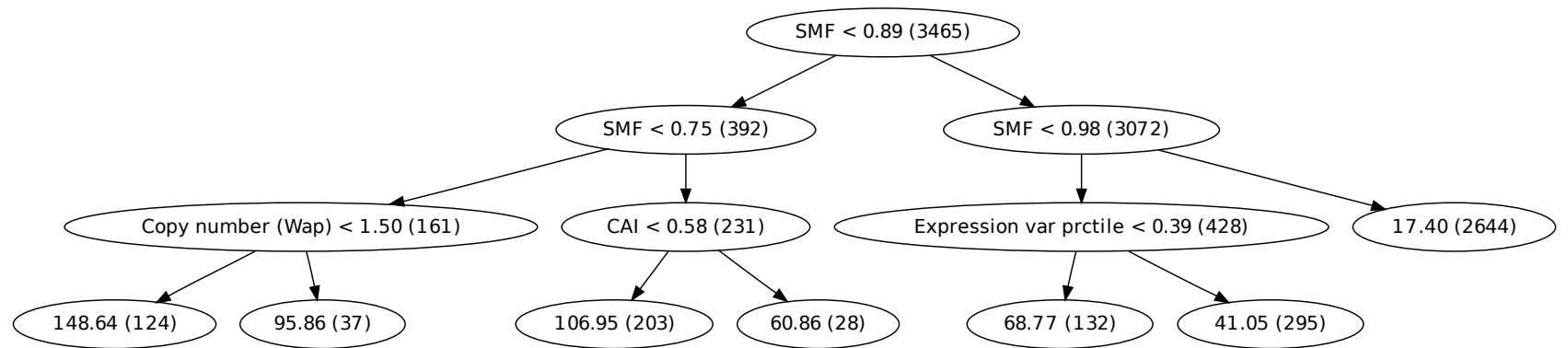

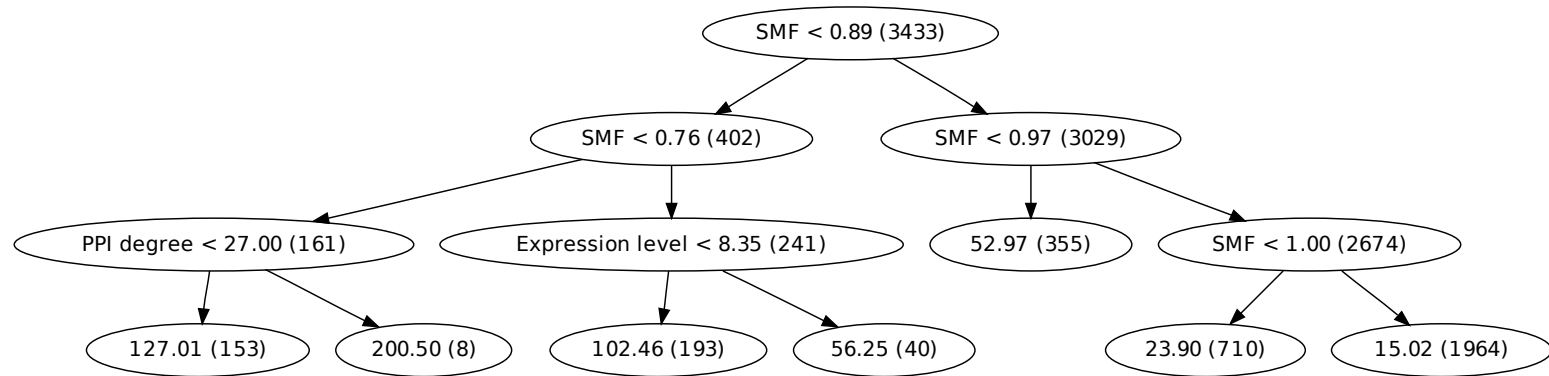

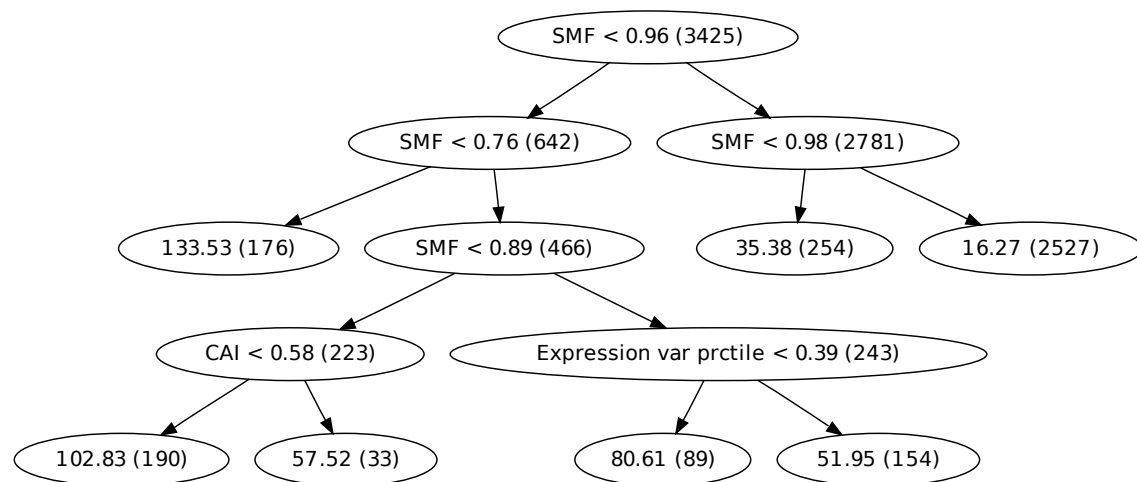

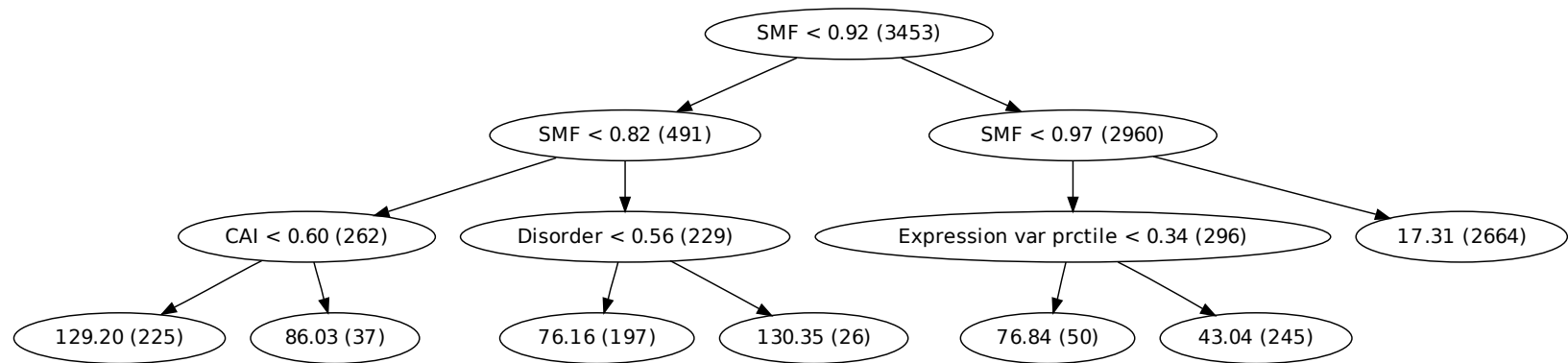

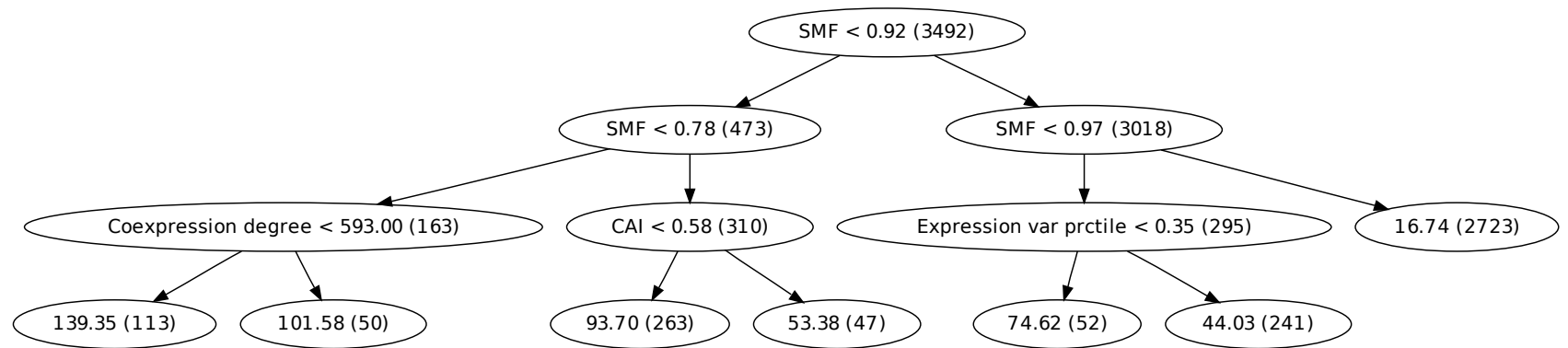

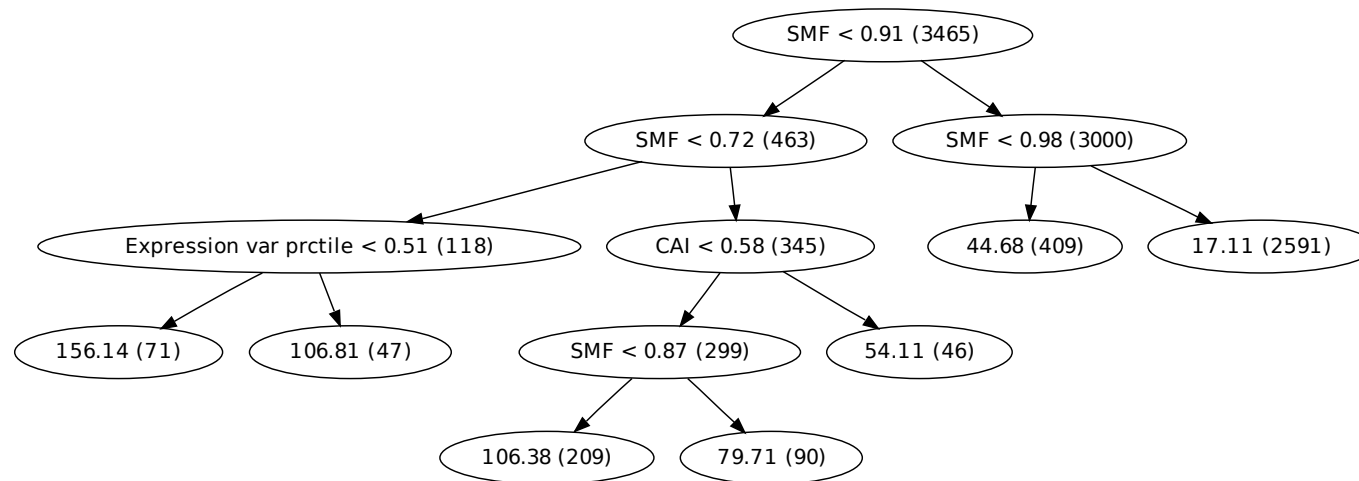

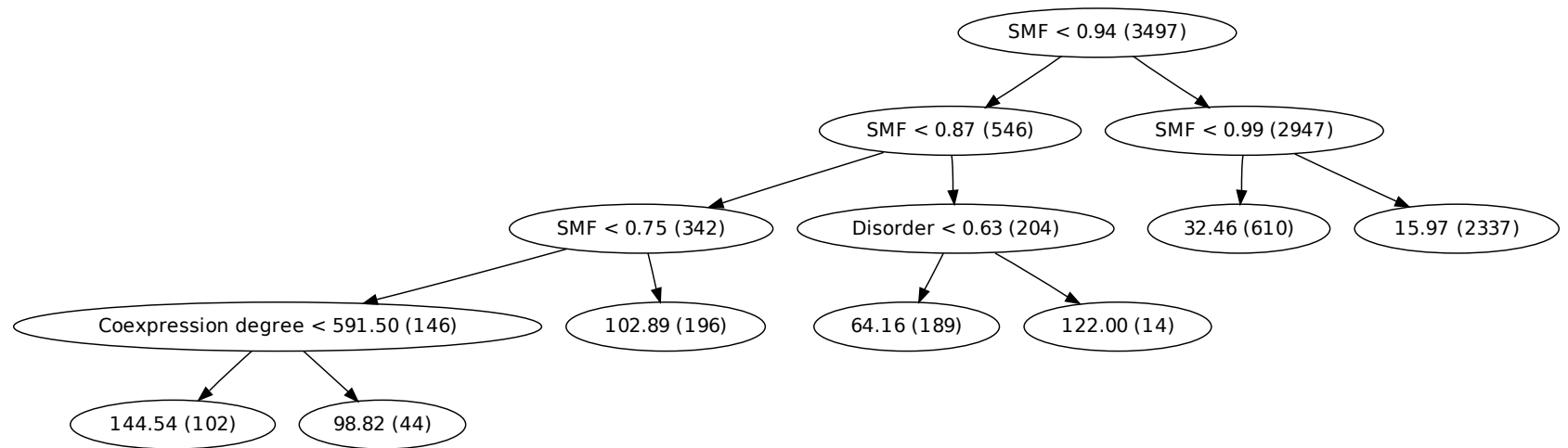

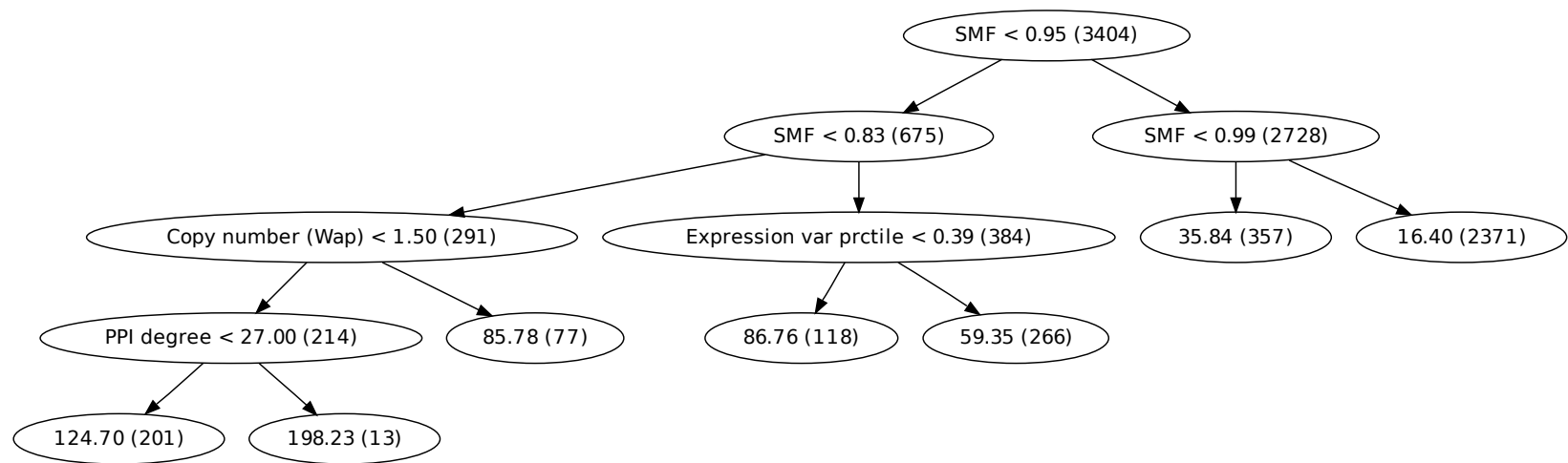

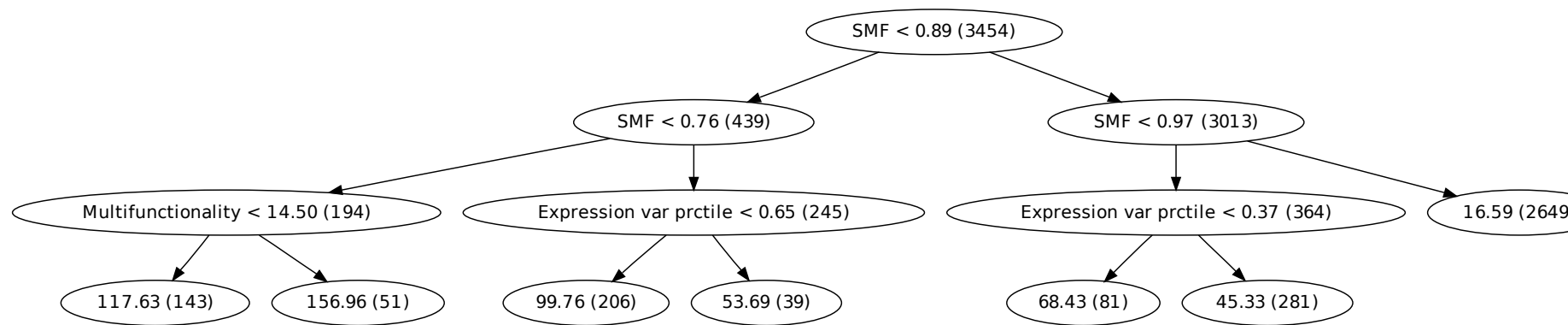

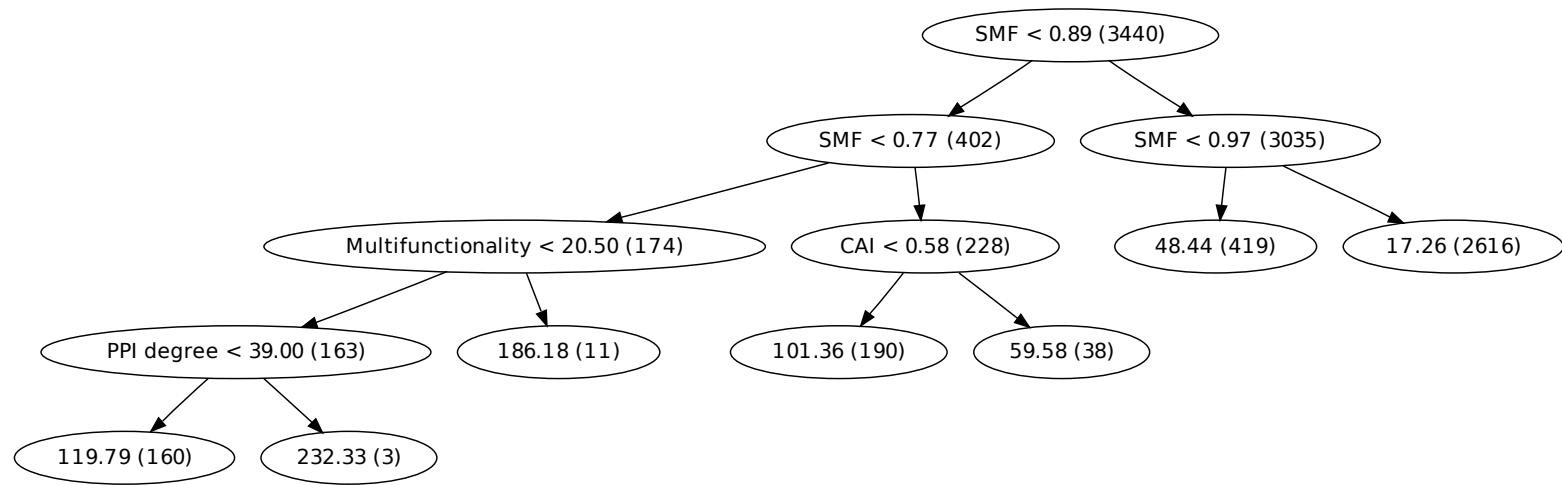

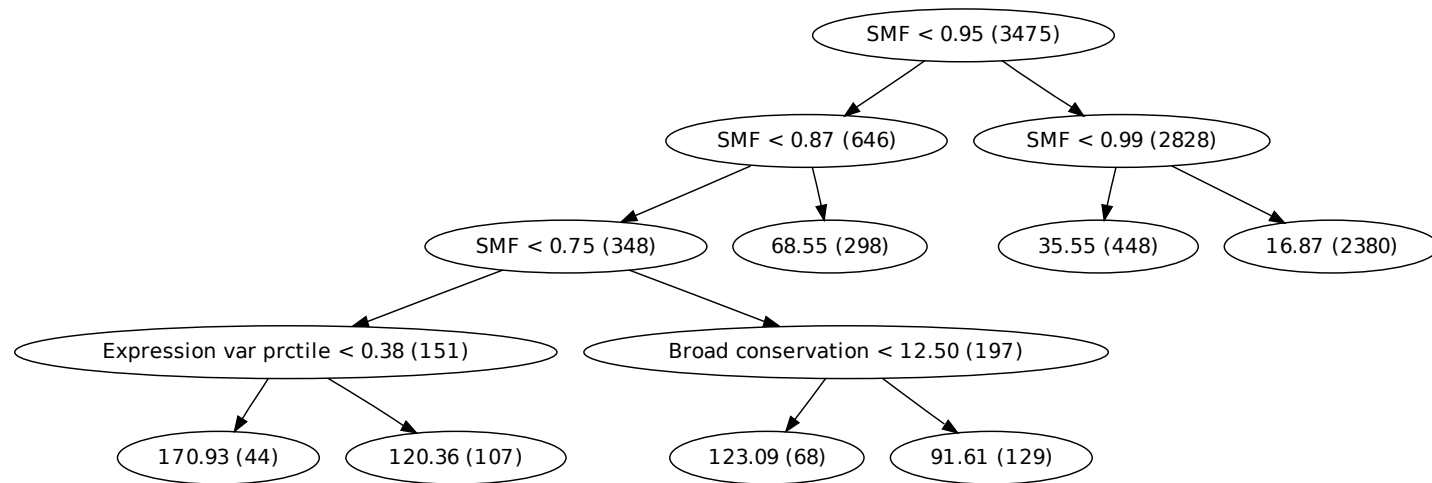

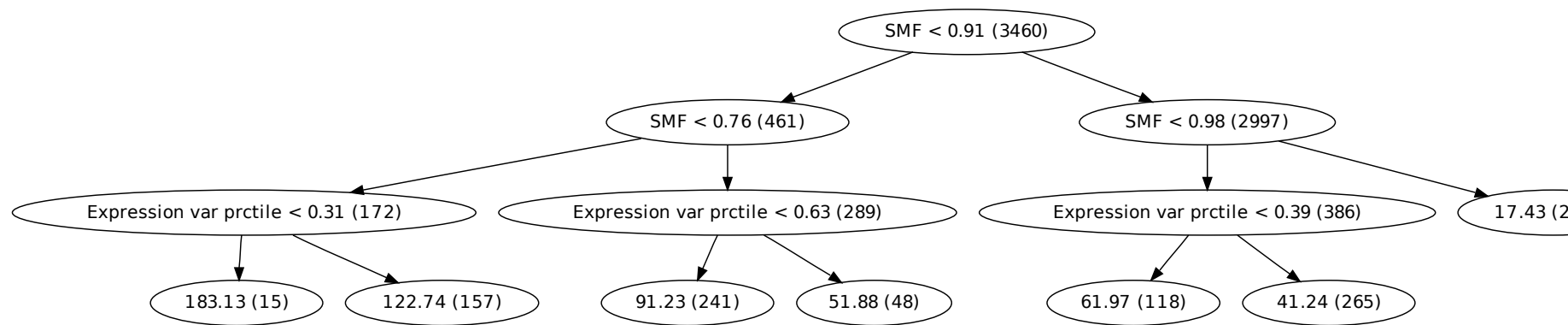

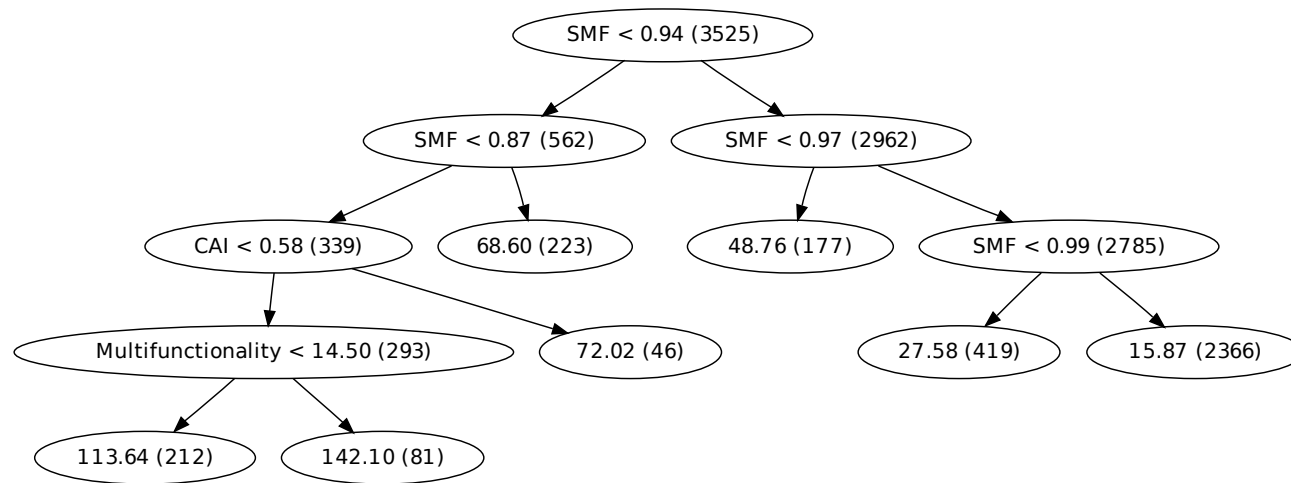

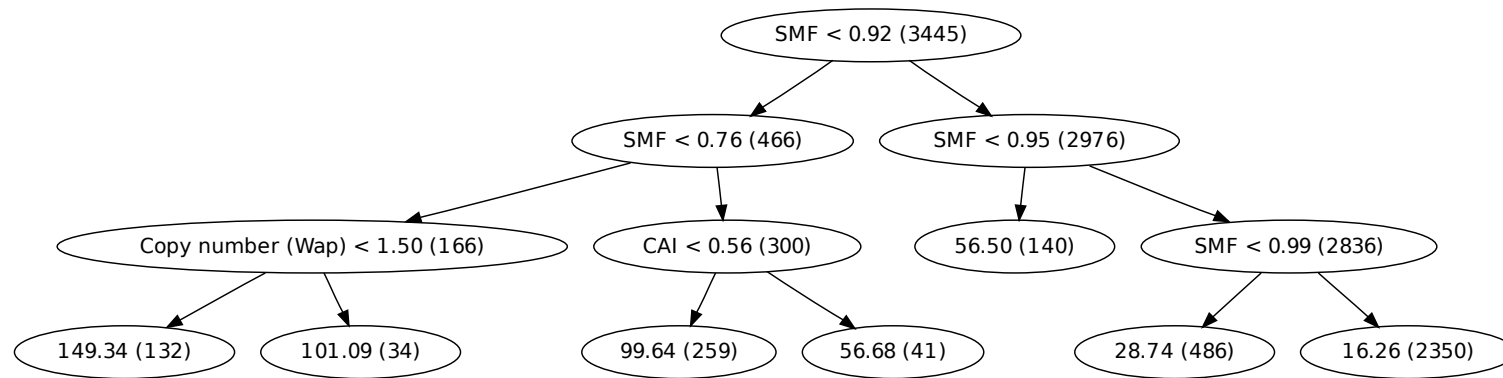

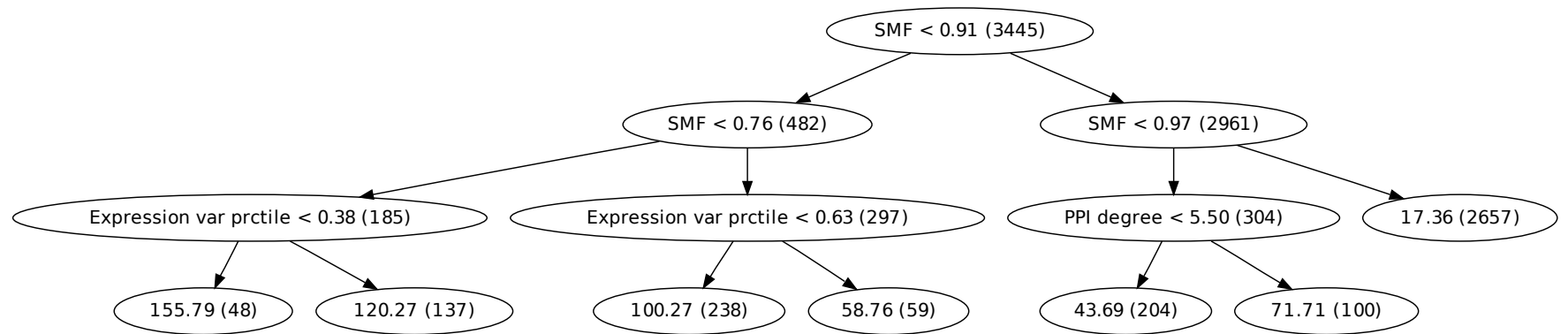

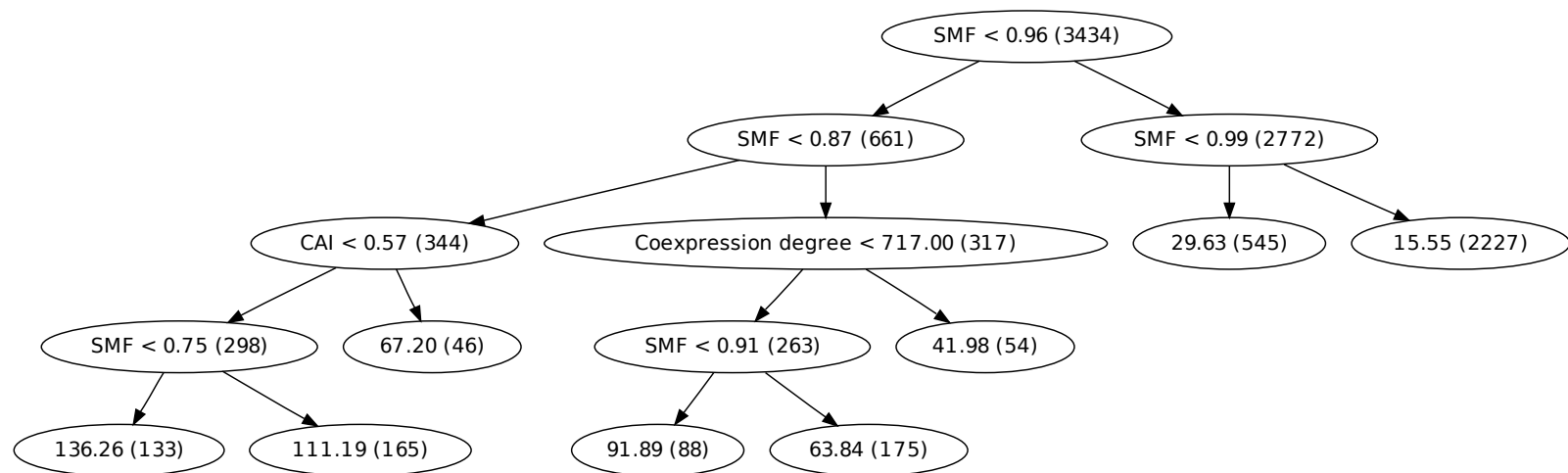

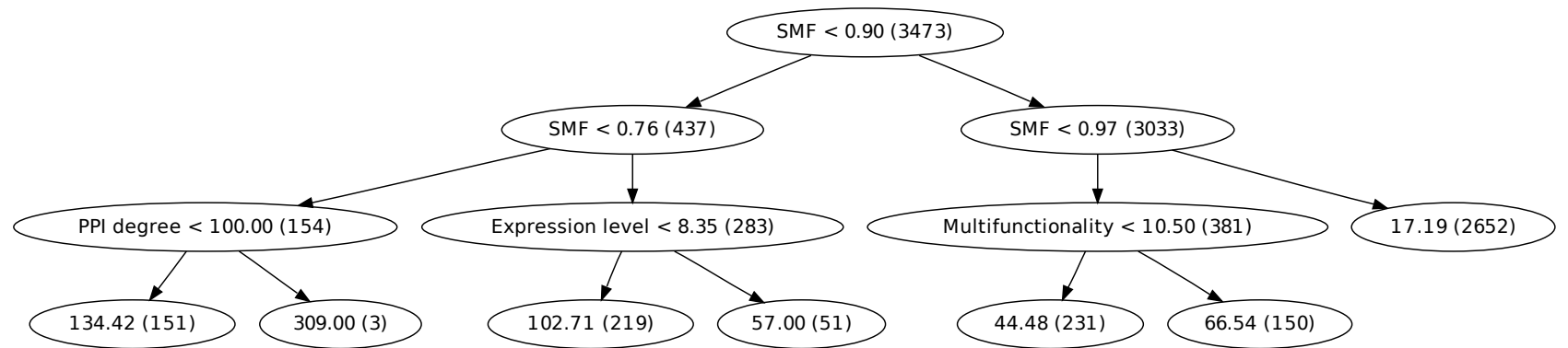

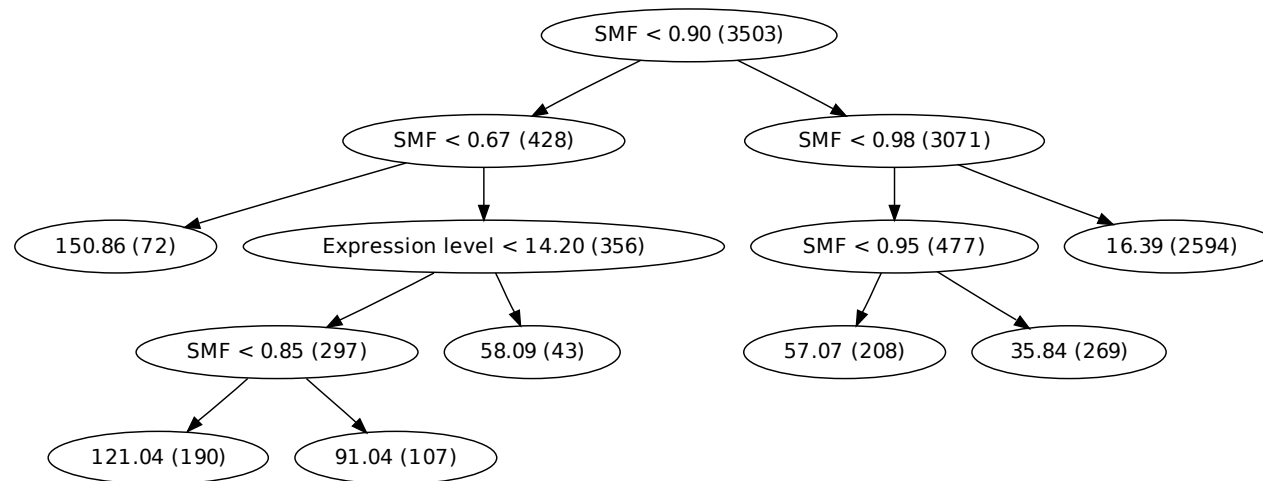

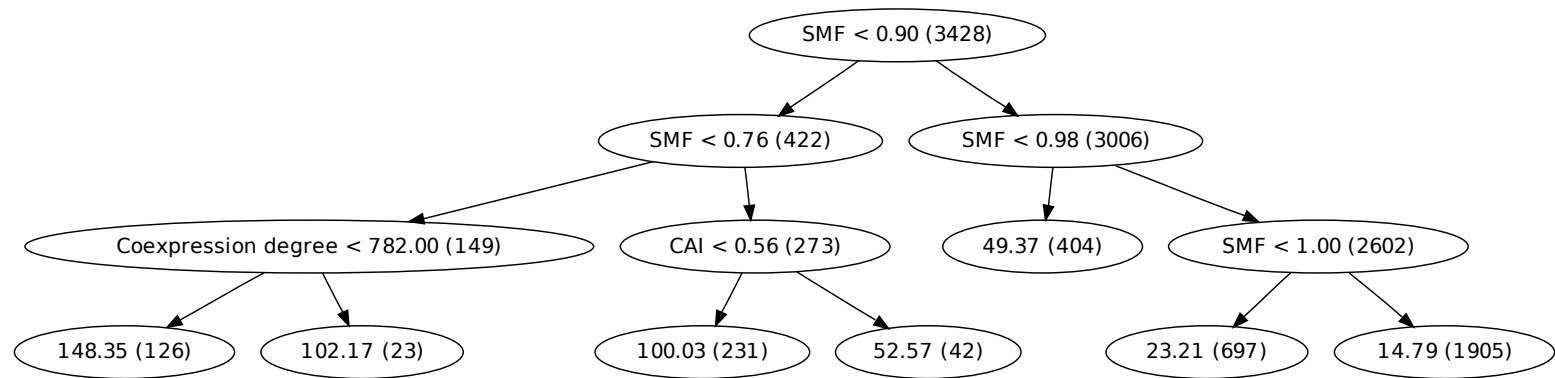

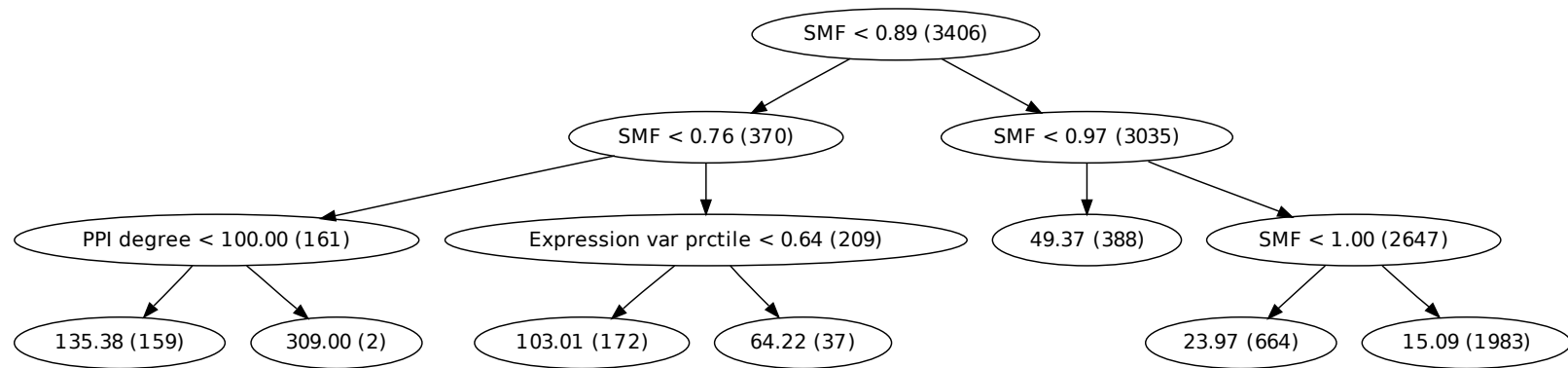

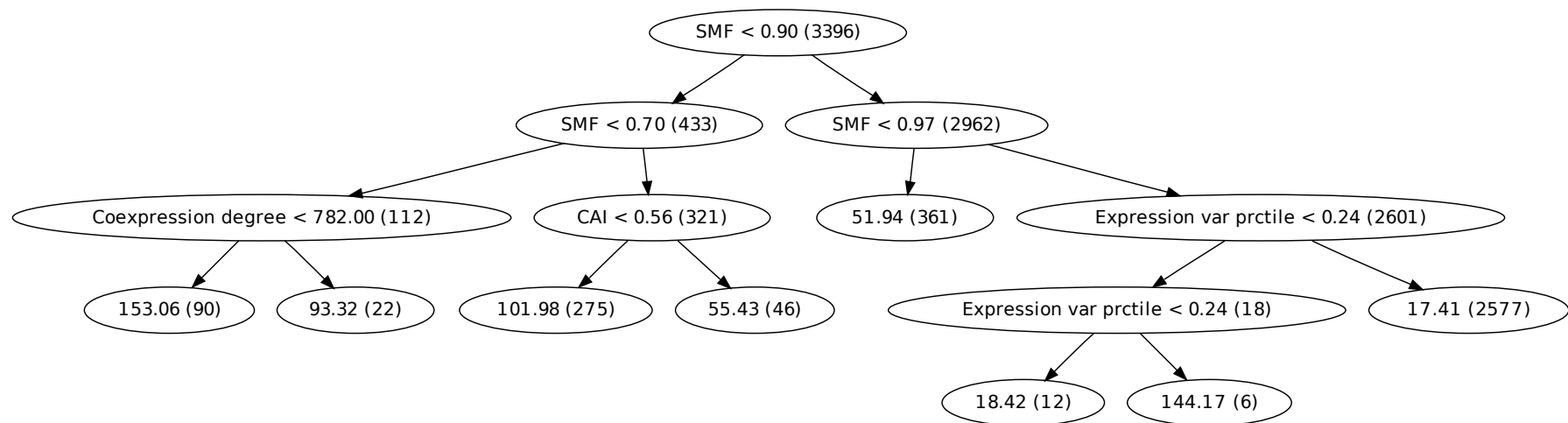

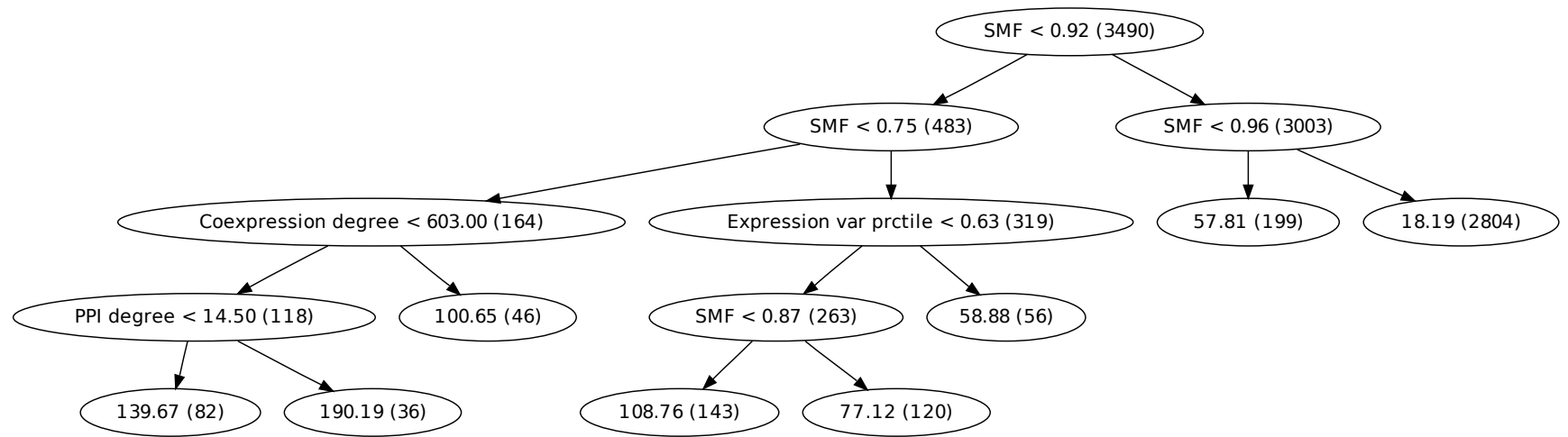

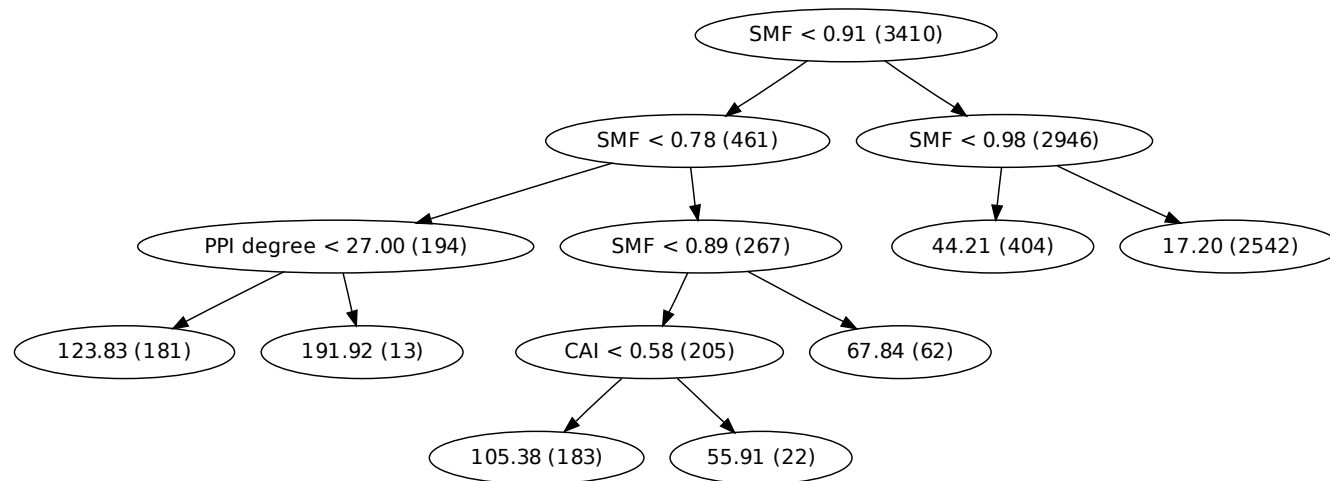

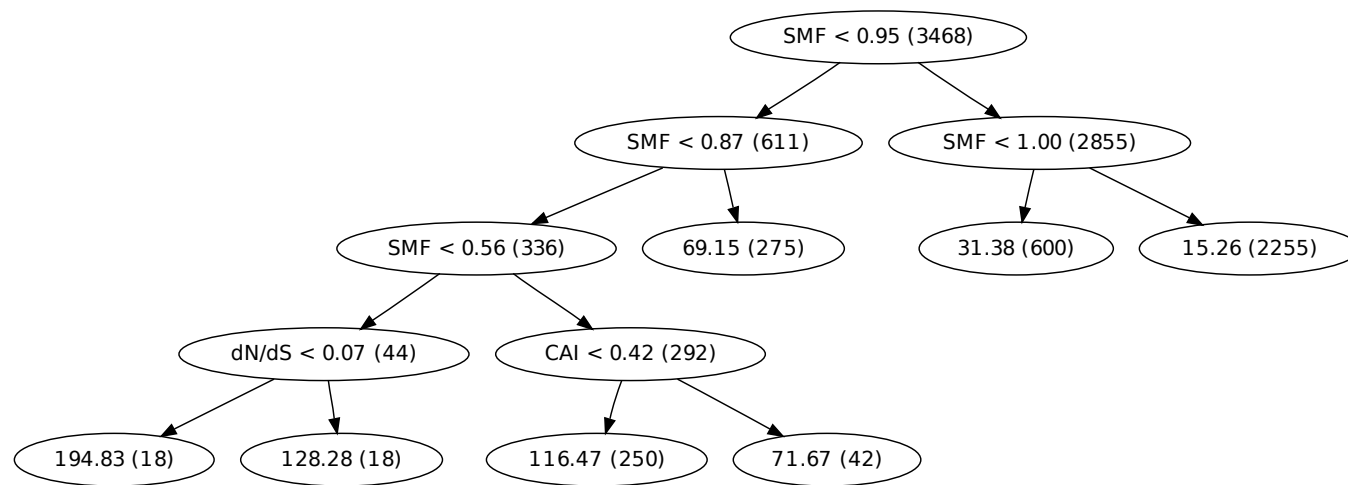

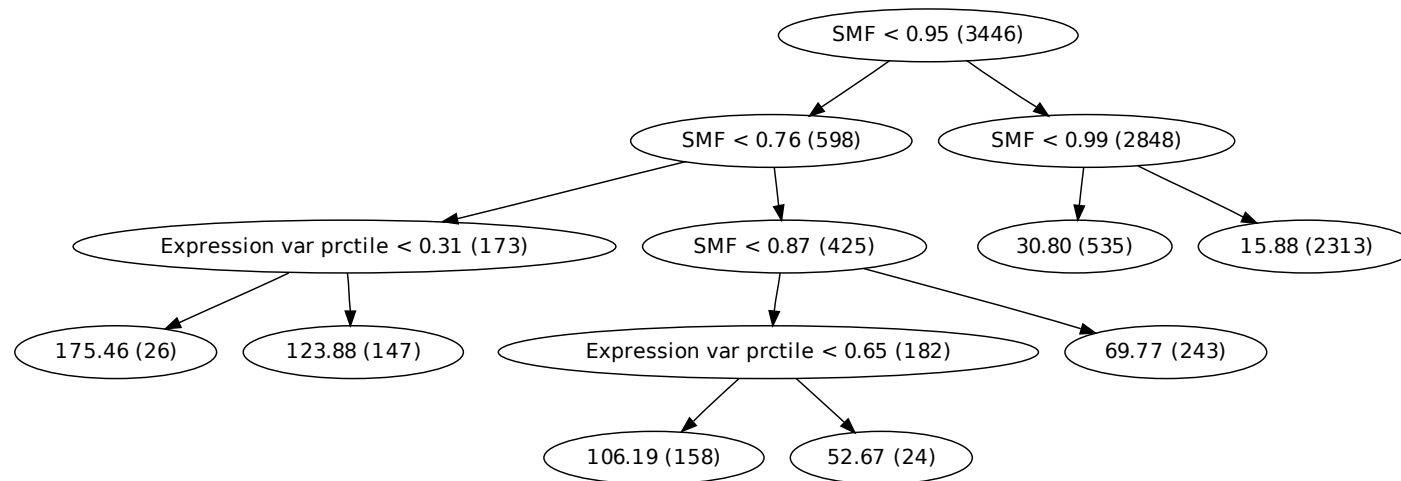

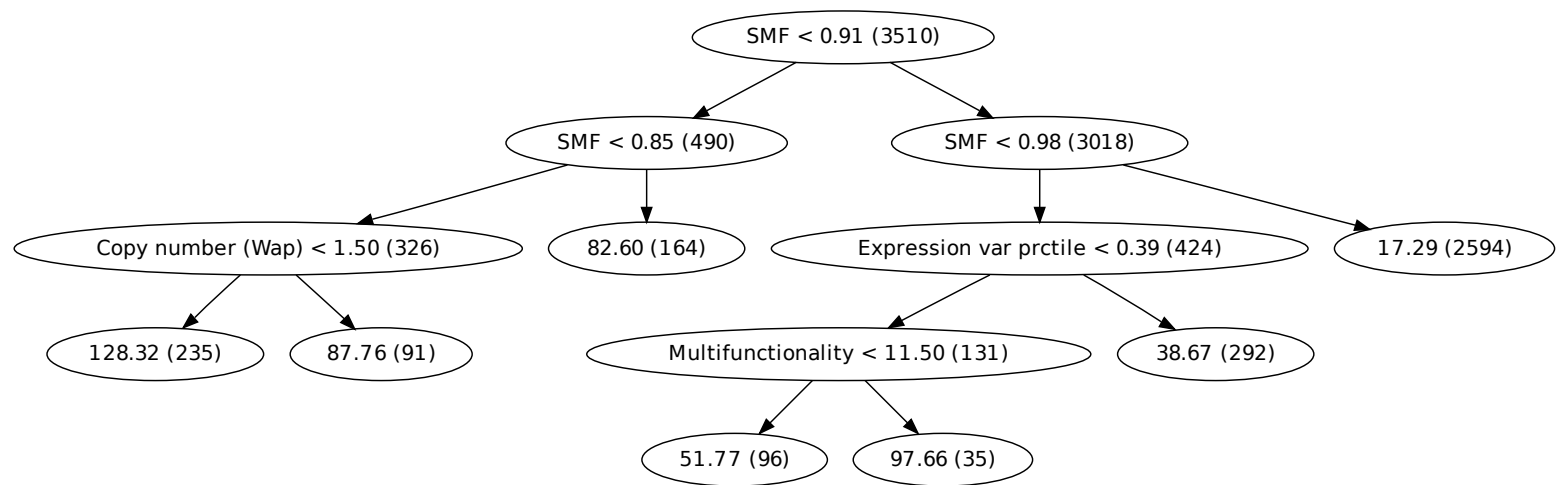

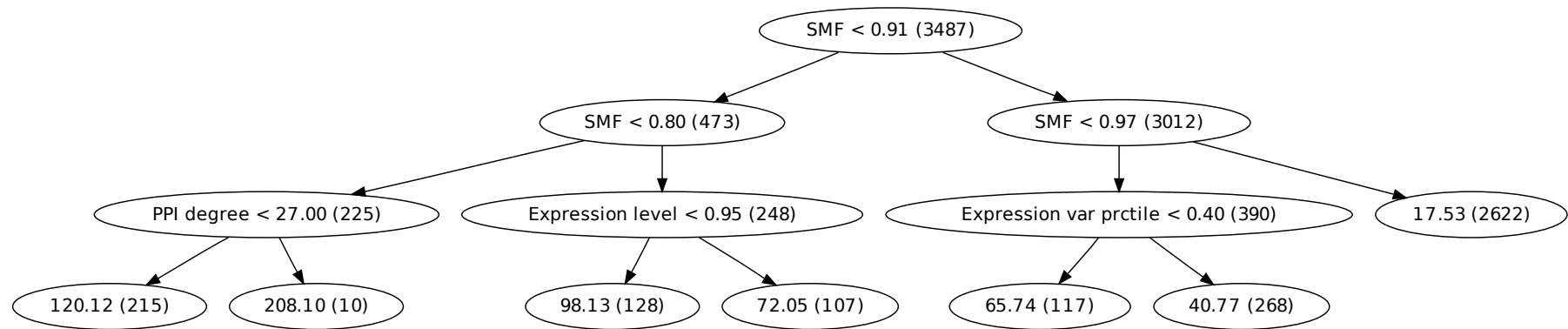

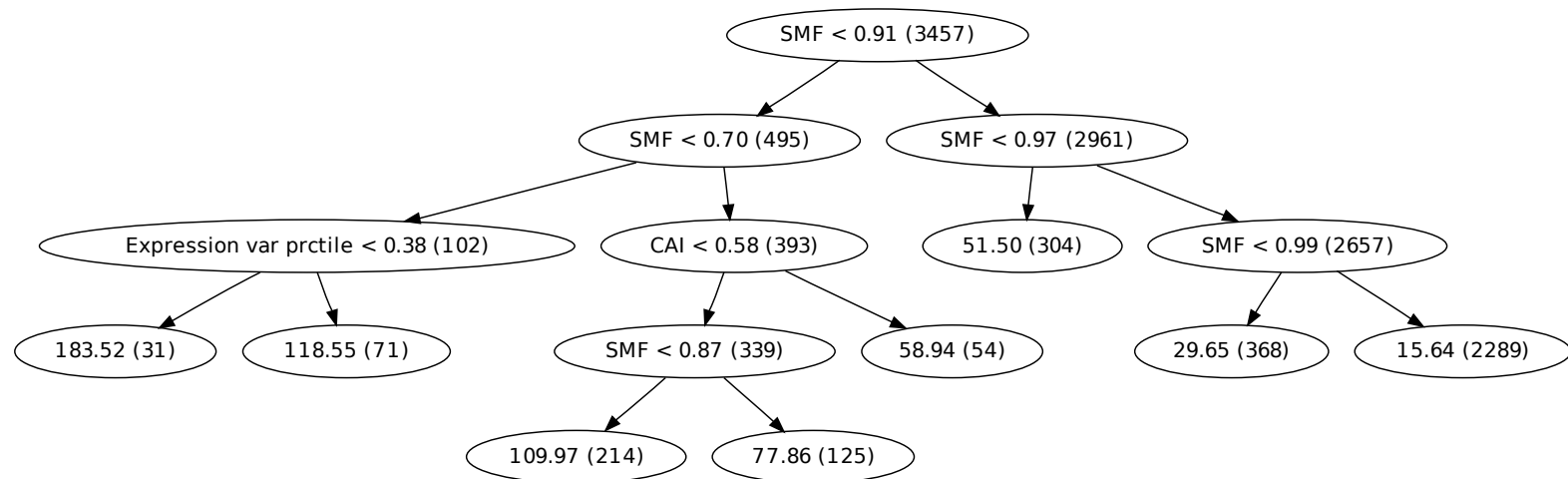

Supplement: Additional file 6 — Regression trees. Regression trees trained on bootstrap samples of S. cerevisiae gene features and negative genetic interaction degree are pictured. [file gb-2012-13-7-r57-S6.PDF]
